# Proteomic Profiling of Plasma to Uncover Novel Intervention Targets and Prognostic Biomarkers for Chronic Liver Diseases

**DOI:** 10.1111/dom.70696

**Published:** 2026-03-26

**Authors:** Xinxuan Li, Jing Sun, Jianhui Zhao, Fangyuan Jiang, Meng Zhang, Hao Wu, Shuai Yuan, Xue Li, Juan Lu, Christos S. Mantzoros

**Affiliations:** ^1^ State Key Laboratory for Diagnosis and Treatment of Infectious Diseases, National Clinical Research Center for Infectious Diseases, Collaborative Innovation Center for Diagnosis and Treatment of Infectious Diseases, the First Affiliated Hospital, College of Medicine Zhejiang University Hangzhou Zhejiang China; ^2^ Center of Clinical Big Data and Analytics of the Second Affiliated Hospital, School of Public Health Zhejiang University School of Medicine Hangzhou Zhejiang China; ^3^ Department of Gastroenterology Sir Run Run Shaw Hospital, Zhejiang University, School of Medicine Hangzhou Zhejiang China; ^4^ Department of Gastroenterology National Clinical Research Center for Digestive Diseases, Changhai Hospital; National Key Laboratory of Immunity and Inflammation, Naval Medical University Shanghai China; ^5^ Unit of Cardiovascular and Nutritional Epidemiology Institute of Environmental Medicine, Karolinska Institutet Stockholm Sweden; ^6^ Division of Endocrinology, Diabetes and Metabolism, Department of Internal Medicine Beth Israel Deaconess Medical Center, Harvard Medical School Boston Massachusetts USA; ^7^ Section of Endocrinology, Boston VA Healthcare System, Harvard Medical School Boston Massachusetts USA

**Keywords:** drug target, liver disease, prediction model, proteome‐wide Mendelian randomization

## Abstract

**Aims:**

The burden of chronic liver disease (CLD) is increasing. This study aims to identify protein markers for CLD and its progression, and develop a protein‐based risk prediction model.

**Materials and Methods:**

We used proteome‐wide Mendelian randomization (MR), Bayesian colocalization and summary‐data‐based MR with proteomic data from deCODE Genetics to identify CLD‐related proteins. Multivariable MR was used to assess independent protein effects. Protein–protein interaction, druggability and mediation analyses were conducted to prioritise therapeutic targets. We further constructed a protein risk score to predict CLD and composite hepatic event outcomes, comparing its performance with existing clinical predictors.

**Results:**

Genetically predicted levels of 16, 5 and 4 plasma proteins were associated with metabolic dysfunction‐associated steatotic liver disease (MASLD), alcoholic liver disease (ALD) and cirrhosis, respectively. IGSF3, FTCD, DCXR, ADH1B and ACY1 were associated with CLD progression. Genetically predicted five modifiable factors (body mass index, waist‐hip ratio, glycated haemoglobin, type 2 diabetes, leisure television watching) were associated with CLD‐related proteins. Proteomic‐based models showed high predictive performance for ALD (C‐index = 0.89), liver cancer (C‐index = 0.84) and liver failure (C‐index = 0.84) in a healthy population without liver diseases at baseline.

**Conclusions:**

This study identified key circulating protein markers for CLD. Protein‐based profiling demonstrated strong predictive potential for CLD and related outcomes.

**Abbreviations**
A1BG: alpha‐1B‐glycoprotein
ACY1: aminoacylase‐1
ADH1A: alcohol dehydrogenase 1A (class I)
ADH1B: alcohol dehydrogenase 1B
AKR7A3: Aldo‐Keto Reductase Family 7 Member A3
ALD: alcoholic liver disease
AUC: area under the curve
BMI: body mass index
BMP1: bone morphogenetic protein 1
C4BPA: complement component 4 binding protein alpha
CLD: chronic liver disease
ComRS: combined risk score
DCXR: dicarbonyl and L‐xylulose reductase
ERBB3: Erb‐B2 receptor Tyrosine Kinase 3
F9: coagulation Factor IX
FBLN1: fibulin 1
FTCD: formimidoyltransferase cyclodeaminase
GKRP: glucokinase regulatory protein
GWAS: genome‐wide association studies
HbA1c: glycated haemoglobin
HEIDI: heterogeneity in dependent instruments
HR: hazard ratio
HTR7: 5‐hydroxytryptamine receptor 7
ICD: International Classification of Diseases
IGDCC4: immunoglobulin superfamily DCC subclass member 4
IGSF3: immunoglobulin superfamily member 3
IL11RA: interleukin 11 receptor subunit alpha
IVW: inverse‐variance weighted
LD: linkage disequilibrium
LTW: leisure television watching
MASLD: metabolic dysfunction‐associated steatotic liver disease
MR: Mendelian randomization
MREG: melanoregulin
MVMR: multivariable Mendelian randomization
NAB1: NGFI‐A binding protein 1
NCAN: neurocan core protein
PPI: protein–protein interaction
pQTL: protein quantitative trait loci
ProRS: proteomic risk score
PWMR: proteome‐wide Mendelian randomization
ROC: receiver operating characteristic
SD: standard deviation
SMR: summary‐data‐based Mendelian randomization
SNP: single nucleotide polymorphism
T2D: type 2 diabetes
TK2: thymidine kinase 2
WC: waist circumference
WHR: waist‐hip ratio

## 1 Introduction

Chronic liver disease (CLD), a condition with high prevalence and poor clinical prognosis, includes metabolic dysfunction‐associated steatotic liver disease (MASLD), alcoholic liver disease (ALD) and advanced liver disease such as cirrhosis and liver cancer. These advanced conditions are frequently associated with serious complications, including ascites, variceal bleeding, spontaneous bacterial peritonitis (SBP), hepatic encephalopathy and liver failure [1], which further complicate the clinical management and significantly worsen the prognosis for patients. It is of great significance to explore early diagnostic biomarkers and develop therapeutic targets for CLD and its associated composite hepatic event (CHE).

The liver, as a protein‐synthesising organ, is responsible for 85%–90% of circulating proteins [2]. In recent years, the potential role of proteins in disease pathophysiology has attracted extensive attention. However, most of these studies were limited to observational studies, small coverage of proteins or insufficient sample sizes. Utilising genetic variants to proxy biological effects of relevant biomarkers, Mendelian randomization (MR) analysis can be used to explore potential causal relationships [3].

Under the framework of comprehensive MR analysis, this study elucidated the role of circulating protein biomarkers in liver disease risk by integrating human genome and plasma proteome data. To ensure independence from major lifestyle and metabolic factors, we adjusted for obesity, smoking, alcohol consumption, diet, physical activity and type 2 diabetes (T2D). We further explored the potential of these proteins as targets for both pharmacological and nonpharmacological interventions, clarified the protein‐mediated pathways between modifiable factors and CLDs, and identified proteins associated with CLD progression. Furthermore, a proteomic risk score (ProRS) was constructed and validated to predict the risk of CLDs and CHEs.

## 2 Materials and Methods

### 2.1 Study Design

We first explored CLD‐related protein biomarkers using a proteome‐wide MR analysis supplemented by colocalization analysis, summary‐data‐based MR (SMR) and heterogeneity in dependent instruments (HEIDI) test. A multivariable MR analysis was employed to isolate the independent effects of proteins on CLDs while accounting for confounders such as smoking, drinking, diet, physical activity, obesity and diabetes. Protein–protein interaction (PPI), druggability evaluation and mediation analyses were performed to prioritise the potential therapeutic targets and illustrate the protein pathway linking modifiable risk factors and risk of CLDs. Additionally, the proteins were examined for their associations with the occurrence of CLDs and CHEs and were distinguished based on their relevance to CLD progression. A protein risk score (ProRS) was constructed to predict CLDs and CHEs, and its predictive power was compared with established clinical predictors.

### 2.2 Summary‐Data‐Based Mendelian Randomization Study

#### 2.2.1 Data Sources for Proteome, Modifiable Factors and Liver Diseases

The GWAS summary statistics for 4719 circulating proteins were extracted from a large‐scale Protein Quantitative Trait Locus (pQTL) study involving 35 559 Icelanders with SOMAscan version 4 [4]. Additionally, using GWASs from European individuals, we examined genetic instruments for 10 obesity‐related factors, 14 lifestyle factors and 21 dietary factors (Table S1).

In the discovery stage, we employed summary statistics from the FinnGen study Round 12 [5], which included 3769 cases and 485 213 controls for ALD and 947 cases and 378 749 controls for liver cancer. Similarly, we used GWAS data from Ghodsian et al. [6] for MASLD, which included 8434 cases and 770 180 controls, and from Wong et al. [7] for cirrhosis, which included 5770 cases and 487 780 controls. In the validation stage, summary‐level statistics were extracted from the UK Biobank [8] comprising 1044 cases and 419 487 controls for ALD, 539 cases and 419 992 controls for liver cancer. Additionally, we included GWAS data from Anstee et al. [9] with 1483 cases and 17 781 controls for MASLD, and FinnGen GWAS Round 12 data with 5545 cases and 494 803 controls for cirrhosis.

#### 2.2.2 Proteome‐Wide Mendelian Randomization (PWMR) Analysis

We first applied a genome‐wide *p*‐value threshold (*p* < 5 × 10^−8^) and then excluded SNPs in the major histocompatibility complex region (chr 6: 25.5–34.0 Mb). Besides, LD clumping was performed to identify independent pQTLs for each protein (*r* ^2^ < 0.001 and clump windows = 1000 kb). The F statistic was calculated to assess the strength of the instrumental variables. Finally, a total of 3896 proteins with *cis*+*trans* SNPs and 1112 proteins with only *cis*‐SNPs were included.

In the MR analysis, *cis*‐pQTLs and *cis*+*trans* pQTLs were used as instruments to explore the associations between genetically predicted protein levels and CLDs. When proteins had more than one instrument, we used the inverse‐variance weighted (IVW) method with fixed effects as the main method if there was no heterogeneity; otherwise, the IVW method with random effects was applied. The Wald‐ratio method was performed when only one SNP was available. We also estimated heterogeneity by calculating Cochran's *Q* value and tested for pleiotropy using the MR‐Egger intercept [10]. Bonferroni correction was applied with *p*‐value < 4.50 × 10^−5^ (0.05/1112) and *p*‐value < 1.28 × 10^−5^ (0.05/3896) as thresholds for *cis*‐pQTLs and all pQTLs (*cis*+*trans*), respectively. All statistical analyses were two‐sided and performed in R 4.2.0 software.

#### 2.2.3 Colocalization Analysis and Summary‐Data‐Based MR (SMR) Analysis

The colocalization and summary‐data‐based MR (SMR) analyses were conducted using GWAS summary statistics from the discovery cohort, leveraging its larger sample size to enhance statistical power for detecting shared genetic signals and validating causal associations. SNPs within ±500 kb of the pQTL for each protein were included to perform Bayesian colocalization analysis and a posterior probability (PPH4) ≥ 0.8 was considered as strong evidence. An SMR test with default settings was employed as a sensitivity analysis to verify the causal associations between protein levels and liver diseases. For the SMR analysis, the Bonferroni‐corrected *p*‐value threshold was set to 0.05 divided by the number of proteins passing the original PWMR. The HEIDI test was used to distinguish pleiotropy from linkage disequilibrium, and a *p*‐value < 0.05 was considered to indicate the existence of linkage disequilibrium. The SMR and HEIDI tests were performed using SMR software (version 1.3.1).

#### 2.2.4 Multivariable Mendelian Randomization

To account for potential confounding by lifestyle and metabolic factors, multivariable MR (MVMR) analyses were conducted for the identified proteins using discovery datasets, adjusting for each covariate (body mass index [BMI], waist‐hip ratio [WHR], cigarettes per day, drinks per week, dietary pattern, physical activity and T2D) individually. Multicollinearity was assessed using pairwise correlation coefficients, with a threshold of 0.8. Covariates with correlations exceeding 0.8 were removed to ensure model stability. A comprehensive multivariate adjustment was then performed, incorporating all remaining covariates simultaneously. An IVW model was used to obtain the direct causal effect of each circulating protein on disease risk.

#### 2.2.5 Protein–Protein Interaction, Druggability Evaluation and Pathway Enrichment Analysis

We searched STRING, which contains 9.64 million proteins and 13.8 million protein interactions, to investigate the PPIs. The interactions between these proteins and drugs were further evaluated to explore whether the identified proteins could serve as potential therapeutic targets. DGIdb [11], ChEMBL [12] and DrugBank [13] databases were screened to obtain information on drug names and approved indications. To evaluate the liver targeting and therapeutic feasibility of key proteins, we analysed the tissue expression characteristics of key proteins based on the Human Protein Atlas and the Genotype‐Tissue Expression (GTEx) database. We incorporated single‐cell RNA sequencing data from the Human Protein Atlas to characterise the cell‐type specificity of the key proteins in the liver. For each protein of interest, we accessed the UniProt [14] database and retrieved information on biological functions. To further explore the biological relevance of the 19 *cis* and *trans* pQTL‐associated proteins, pathway enrichment analysis was performed using the Metascape platform [15]. The proteins were annotated against multiple functional databases, including Gene Ontology (GO), Reactome Gene Sets, KEGG Pathway, Immunologic Signatures and Chemical and Genetic Perturbations. Terms with a *p* value < 0.05, a minimum count of 3, and an enrichment factor > 1.5 were retained. Statistical significance was determined using the cumulative hypergeometric distribution, with the Benjamini‐Hochberg procedure applied to correct for multiple comparisons.

#### 2.2.6 Mediation Analyses

To evaluate the associations of 45 modifiable factors with CLDs and CLD‐associated proteins, we first adopted univariate MR analysis with the IVW method as the main analysis. The MR‐Egger, weighted median, simple mode and weighted mode methods were employed as supplementary analyses. Subsequently, MVMR analyses were performed to explore the potential mediation of identified CLD‐associated proteins in the association between each modifiable factor and CLDs. The Bonferroni correction with *p*‐value threshold < 5.29 × 10^−5^ (0.05/(45 factors × 21 proteins)) was used.

### 2.3 Prospective Cohort Study and Prediction Model Development

#### 2.3.1 Study Population

For the protein score prediction analysis, we obtained proteomic measurements from 50 000 participants in the UK Biobank study [16]. After excluding non‐European participants, those with diagnoses of viral hepatitis or other liver diseases at baseline or at any point during follow‐up, those with liver cancer at baseline, those with liver surgery, and those with incomplete laboratory variables, 34 924 individuals remained. We stratified the population into patients with early‐stage CLD (Subgroup 1) and individuals without early‐stage CLD (Subgroup 2). We additionally stratified the population into patients with cirrhosis (Subgroup 3) and individuals without cirrhosis (Subgroup 4). Lastly, patients with baseline MASLD, ALD, cirrhosis, ascites, variceal bleeding, SBP, hepatic encephalopathy or liver failure were excluded, and a total of 34 778 participants were included. The flowchart is presented in Figure S1.

#### 2.3.2 Ascertainment of Plasma Protein Variables and Liver Diseases

We investigated 10 available plasma proteins (i.e., A1BG, ACY1, DCXR, ERBB3, F9, FTCD, IGDCC4, IGSF3, NCAN and ADH1B) from the UKB Pharma Plasma Proteome cohort, which used the Olink Explore 3072 platform to perform proteomic analysis of plasma samples from 54 219 individuals, measuring 2941 protein analytes out of 2923 unique proteins.

Study endpoints were four CLDs, including MASLD, ALD, cirrhosis and liver cancer, and five CHEs, including ascites, variceal bleeding, SBP, hepatic encephalopathy and liver failure. The diagnosis of CLDs and CHEs was ascertained using the Read version 2 and Clinical Terms Version 3 clinical codes and the International Classification of Diseases (ICD) codes. MASLD and ALD were defined as mild stages of liver disease and did not include liver fibrosis or cirrhosis. The definition of MASLD further excluded excess alcohol consumption (males with > 30 g/day and females with > 20 g/day ethanol intake) and viral hepatitis infection. The full list of ICD codes used in this study is presented in Table S2.

#### 2.3.3 Identification of Plasma Proteins in CLDs, CHEs and Disease Progression

To identify plasma proteins associated with CLDs, CHEs and disease progression, we conducted a Cox regression analysis of circulating proteins with CLD and CHE outcomes. Subsequently, we explored the role of these proteins in CLD progression within Subgroups 1 and 2. Additionally, we examined proteins associated with liver cancer development in cirrhotic and non‐cirrhotic livers within Subgroups 3 and 4. To explore synergistic or antagonistic interactions among the 10 ProRS component proteins, pairwise PPI terms were incorporated into the Cox proportional hazards regression model for CLD. Hazard ratios (HRs) and 95% confidence intervals (CIs) were assessed using Cox proportional hazards models. The proportional hazards assumption was tested using Schoenfeld residuals. An FDR‐adjusted *p* value < 0.05 was considered statistically significant after multiple testing correction. Several confounders were incorporated into the models, namely age, sex, Townsend deprivation index, education level, smoking status, alcohol consumption, BMI, physical activity, healthy diet, waist circumference, hyperlipidemia and hypertension. For missing values of covariates, we used imputation with sex‐stratified median for continuous variables and mode for categorical variables. The missing rate of all covariates was less than 5% and the missing pattern was completely random. Detailed information on missing data of each covariate is provided in Table S3.

#### 2.3.4 Construction of Risk Score and Development of Prediction Model

We conducted a Cox proportional hazards regression for the available proteins selected by MR analysis based on *cis*+*trans* and *cis*‐only pQTLs to compute the ProRS. A total of 10 proteins (i.e., A1BG, ACY1, DCXR, ERBB3, F9, FTCD, IGDCC4, IGSF3, NCAN and ADH1B) had available data and were used to calculate a weighted ProRS. In addition to these proteins, age and sex were incorporated into the model as key components. The ProRS was calculated using the following formula: risk score = h0(t)*exp. (*β*_1_ *X*_1_ + *β*_2_ *X*_2_ + ··· + *β*_10_ *X*_10_ + *β*_age_ × _age_ + *β*_sex_ × _sex_), where *X*_1_–*X*_10_ represented the level of protein and *β*_1_ to *β*_10_ were the coefficients of proteins associated with disease risk derived from a Cox model, *β*_age_ and *β*_sex_ were the coefficients of age and sex on disease risk, and h0(t) was the baseline hazard. We then built a prediction model to explore whether these selected proteins could achieve good predictive performance for CLD and CHE. Next, we combined the ProRS with the LiverRisk score [17] to calculate the combined risk score (ComRS) and explore its additive predictive value for predicting the risk of CLD and CHE onset. The LiverRisk score was composed of eight variables, including age, sex, fasting glucose, cholesterol, aspartate aminotransferase, alanine aminotransferase, gamma‐glutamyl transferase and platelet count. The fibrosis‐4 (FIB‐4) index was further calculated using available data from the UK Biobank, including age, aspartate aminotransferase, alanine aminotransferase and platelet count. Participants from the UK Biobank (*n* = 34 778) were randomly stratified into training and validation samples with a 7:3 ratio for internal validation. Ten‐fold cross‐validation was applied in the training set.

#### 2.3.5 Model Assessment and Risk Stratification Analysis

The ProRS model was compared with models of baseline characteristics (sex, age, smoking, physical activity, diet), alcohol consumption, LiverRisk score [17], FIB‐4 index and ComRS. The discrimination of these models was assessed using the C‐statistic with the bootstrap method. We further performed subgroup analyses stratified by age (< 50 vs. ≥ 50 years), sex (male vs. female) and obesity status (BMI ≤ 24 kg/m^2^ vs. BMI > 24 kg/m^2^) to evaluate the predictive stability of ProRS across populations. Net reclassification improvement (NRI) and integrated discrimination improvement (IDI) were used to quantify the additive predictive value of the ProRS when added to the LiverRisk score. Decision curve analysis (DCA) was used to evaluate the clinical utility of the ProRS. Additionally, we investigated the predictive value of ProRS under different incident time windows at 5, 10 and 15 years of follow‐up and reported the area under the Receiver Operating Characteristic (ROC) curve (AUC). Calibration of the risk model was conducted using the Hosmer‐Lemeshow test by comparing the predicted versus the observed incident liver disease [18]. Based on the quantiles of ProRS, participants were classified into three risk groups (low‐risk, moderate‐risk and high‐risk). We then calculated the cumulative incidence of CLDs and CHEs for the three different risk categories using Kaplan–Meier curves and performed log‐rank tests. To validate the clinical relevance of these risk cutoffs, we further estimated the 10‐year absolute risk of CLDs and CHEs using a Cox proportional hazards model and assessed whether the high‐risk group exceeded a clinically meaningful threshold of 5%.

## 3 Results

### 3.1 Selection of Genetic Instruments

We retained 9512 pQTLs for 3896 proteins as instruments in MR analysis (Table S4), including 1112 *cis*‐pQTLs for 1112 unique proteins and 8400 *trans*‐pQTLs for 3608 proteins. Of 3896 proteins with instruments, 824 were instrumented by both *cis*‐ and *trans*‐pQTLs, 288 by only *cis* instruments and 2784 by only *trans* instruments. The *F* statistics for all used genetic instruments were greater than 10, suggesting no substantial weak instrument bias. The number of instrumental variables for modifiable risk factors is shown in Table S1.

### 3.2 Identification of Plasma Proteins Associated With CLDs Using *Cis*‐Only pQTLs

**FIGURE 1:**
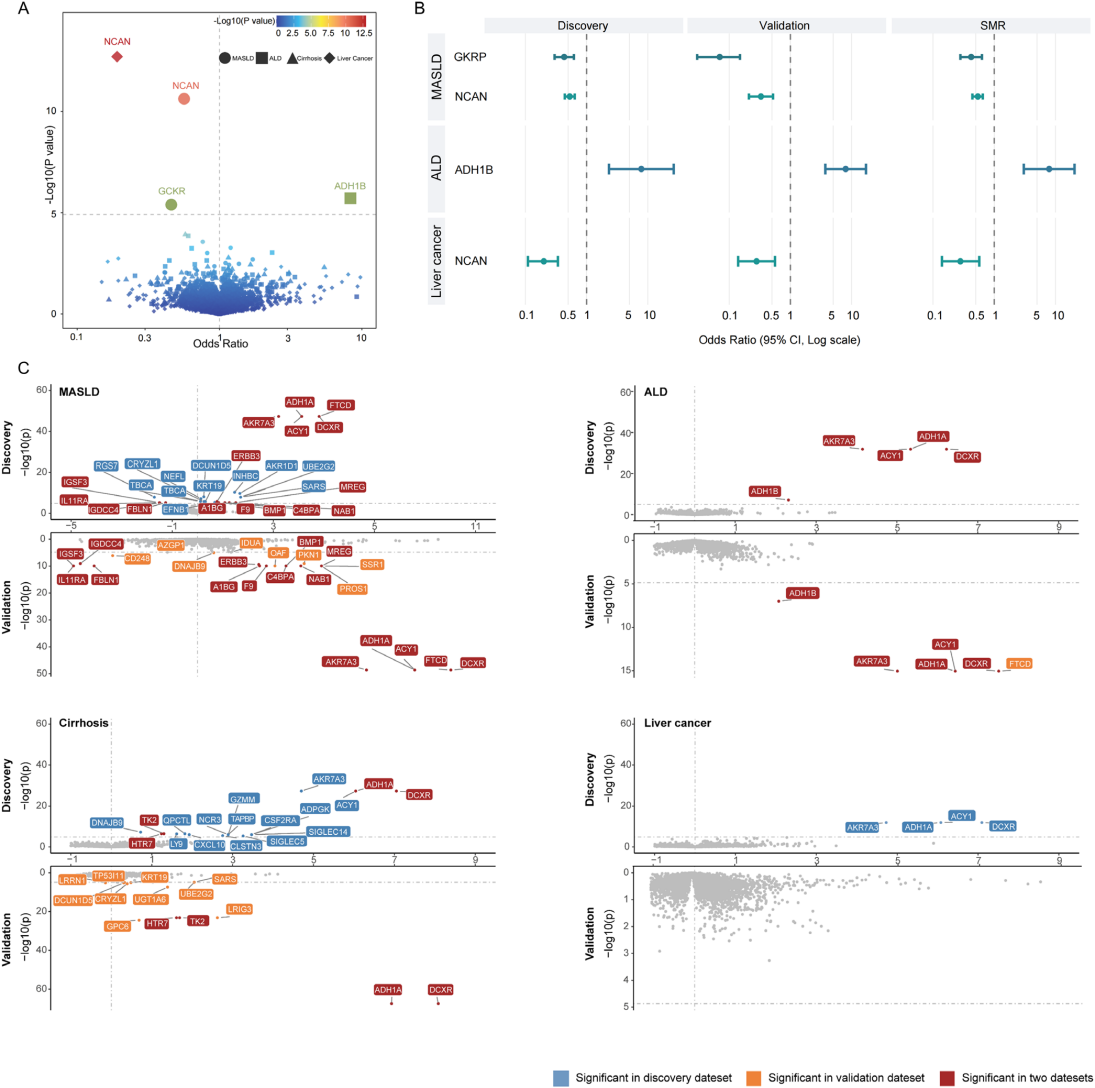
Identification of plasma proteins associated with chronic liver disease. (A) Volcano plots of proteome‐wide Mendelian randomization using *cis*‐pQTLs. (B) Summary results from discovery and validation Mendelian randomization and summary‐data‐based Mendelian randomization for three identified proteins using *cis*‐pQTLs. (C) Volcano plots of proteome‐wide Mendelian randomization using all pQTLs. The *x* axis is the beta value of causal association between protein and liver disease calculated by inverse variance weighted. The *y* axis is the −log_10_ (*p*) of the MR estimate in discovery (upper) and validation (bottom) datasets. ALD, alcoholic liver disease; CI, confidence interval; MASLD, metabolic dysfunction‐associated steatotic liver disease; SMR, summary‐data‐based Mendelian randomization.

Using *cis*‐pQTLs, a total of three proteins were significantly associated with CLD risk after correction (*p* < 4.50 × 10^−5^) in both the discovery and validation sets. As shown in Figure 1A,B and Table S5, in discovery MR, genetically predicted higher levels of NCAN and GKRP were associated with a decreased risk of MASLD. The odds ratio (OR) (95% CI) of MASLD per SD increase in genetically predicted levels of protein was 0.53 (0.44–0.64) for NCAN and 0.43 (0.30–0.62) for GKRP. We also observed that genetically predicted higher level of ADH1B was associated with an increased risk of ALD (OR = 7.83; 95% CI: 2.32–26.47, *p* = 3.44 × 10^−6^). There was also a suggestive association between genetically predicted higher level of NCAN and a decreased risk of liver cancer (OR = 0.20; 95% CI: 0.11–0.34, *p* = 9.98 × 10^−9^). These associations were consistent across the discovery and validation sets.

### 3.3 Identification of Plasma Proteins Associated With CLDs Using Both *Cis*‐ and *Trans*‐ pQTLs

We used all (*cis*+*trans*) pQTLs as instruments to extend our MR analyses. All results of the proteome‐wide MR are shown in Figure 1C and Table S6. After correction (*p* < 1.28 × 10^−5^), 19 circulating proteins had significant associations with CLDs risk. Out of those proteins, genetically predicted higher levels of 5 proteins (i.e., ADH1A, DCXR, AKR7A3, ACY1, ADH1B) and 4 proteins (i.e., ADH1A, DCXR, HTR7, TK2) showed positive associations with ALD and cirrhosis, respectively. Furthermore, genetically predicted higher levels of 12 proteins were associated with an increased MASLD risk, including ADH1A, DCXR, AKR7A3, A1BG, ACY1, BMP1, C4BPA, ERBB3, F9, FTCD, MREG and NAB1. In contrast, genetically predicted higher levels of FBLN1, IGDCC4, IGSF3 and IL11RA were associated with a decreased risk of MASLD. However, the positive associations were not reproduced for AKR7A3, ADH1A, ACY1 and DCXR with liver cancer.

### 3.4 Plasma Proteins With Strong Evidence From Colocalization, SMR and HEIDI Tests

For the *cis*‐MR prioritised associations, GKRP‐MASLD, ADH1B‐ALD and NCAN‐liver cancer pairs showed strong evidence of colocalization (Figure S2). For the *cis+trans*‐MR prioritised associations, 23 out of the 25 associations had strong evidence of colocalization (Figures S3–S5). The SMR analysis confirmed the significant associations for NCAN with MASLD and liver cancer, GKRP with MASLD and ADH1B with ALD (Table S5). Additionally, associations of ADH1B with ALD and NCAN with liver cancer passed the HEIDI test (*p* > 0.05). For the 19 proteins identified using all (*cis*+*trans*) pQTLs, genetically predicted levels of the four inversely correlated proteins (FBLN1, IGDCC4, IGSF3 and IL11RA) for MASLD failed to pass the SMR analyses (*p* > 0.003). Moreover, the HEIDI test suggested that all observed associations were not due to linkage disequilibrium (Table S6).

### 3.5 Independent Associations of Plasma Proteins With CLDs

**FIGURE 2:**
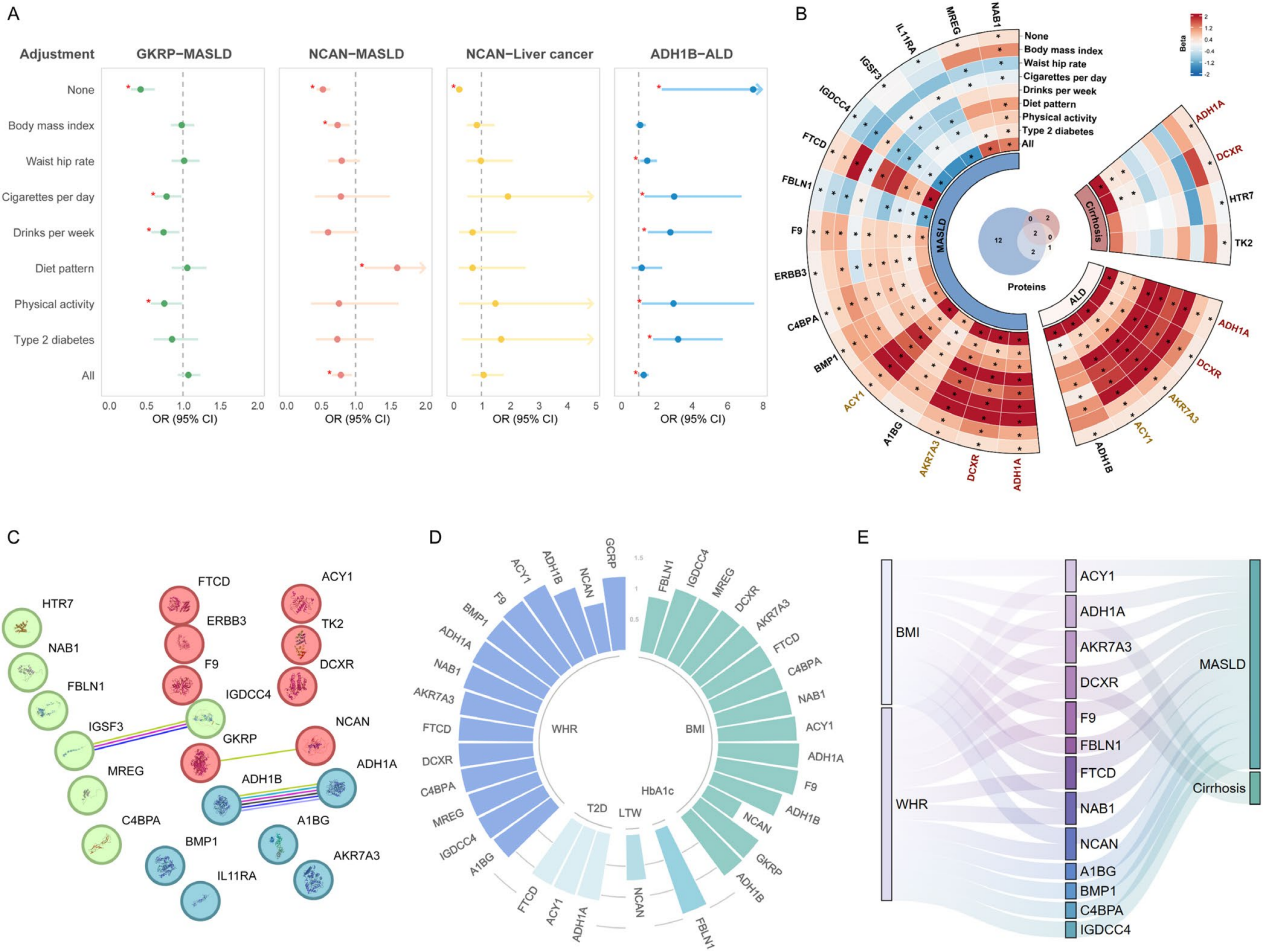
The results of multivariable Mendelian randomization, Protein–protein interaction and mediation analyses. (A) The multivariable Mendelian randomization results of four associations after adjusting covariates using *cis*‐pQTLs. *p < 0.05. (B) The multivariable Mendelian randomization results of associations after adjusting covariates using all pQTLs. *p < 0.05. (C) The Protein–protein interaction network of proteins identified by proteome‐wide Mendelian randomization. Lines represent interactions between proteins. Green line indicates interaction evidence from text mining; Cyan line indicates known interaction from curated databases; Fuchsia line indicates known interaction that is experimentally determined. Black line indicates co‐expression. Blue line indicates predicted interactions from gene co‐occurrence. Light purple line indicates protein homology. (D) The causal associations between five factors and identified proteins. The height of the column represents the odds ratios between factors and proteins. (E) The overview of potential causal mediating network. A1BG, alpha‐1B‐glycoprotein; ACY1, aminoacylase‐1; ADH1A, alcohol dehydrogenase 1A (class I); ADH1B, alcohol dehydrogenase 1B; AKR7A3, Aldo‐Keto Reductase Family 7 Member A3; ALD, alcoholic liver diseases; BMI, body mass index; BMP1, Bone Morphogenetic Protein 1; C4BPA, complement component 4 binding protein alpha; CI, confidence interval; DCXR, dicarbonyl and L‐xylulose reductase; ERBB3, Erb‐B2 Receptor Tyrosine Kinase 3; F9, coagulation Factor IX; FBLN1, Fibulin 1; FTCD, formimidoyltransferase cyclodeaminase; GKRP, glucokinase regulatory protein; HTR7, 5‐hydroxytryptamine receptor 7; IGDCC4, immunoglobulin superfamily DCC subclass member 4; IGSF3, Immunoglobulin superfamily member 3; IL11RA, Interleukin 11 receptor subunit alpha; MASLD, metabolic dysfunction‐associated steatotic liver disease; MREG, melanoregulin; NAB1, NGFI‐A binding protein 1; NCAN, neurocan core protein; OR, odds ratio; TK2, thymidine kinase 2; WHR, waist‐hip ratio.

After adjusting for all covariates with no high correlation (Figure S6), NCAN was robustly associated with MASLD (OR = 0.79; 95% CI: 0.66–0.95, *p* = 0.01), whereas ADH1B was robustly associated with ALD (OR = 1.29; 95% CI: 1.06–1.58, *p* = 0.01) (Figure 2A and Table S7). For the *cis*+*trans*‐MR prioritised associations, genetically predicted higher levels of 10 proteins, including ADH1A, DCXR, AKR7A3, A1BG, ACY1, BMP1, F9, FTCD, MREG and NAB1, were found to be associated with an increased risk of MASLD. Conversely, genetically predicted higher levels of FBLN1, IGDCC4 and IL11RA were associated with a decreased risk of MASLD. Additionally, genetically predicted higher levels of 4 proteins (ADH1A, AKR7A3, ACY1 and ADH1B) were associated with an increased risk of ALD. Notably, ADH1A also showed a positive association with cirrhosis (Figure 2B and Table S8).

### 3.6 PPI and Druggability Evaluation on the Potential of Therapeutic Targets

The interactions between ADH1A and ADH1B, IGSF3 and IGDCC4, GKRP and NCAN were identified among these proteins (Figure 2C). Among these interactions, those between ADH1A and ADH1B, IGSF3 and IGDCC4 were supported by experimental evidence. In the druggability evaluation (Table S9), we found that 12 of these proteins (A1BG, ACY1, ADH1A, C4BPA, ERBB3, F9, FTCD, IL11RA, HTR7, TK2, NCAN, ADH1B) have been targeted for drug development. For instance, the drug Fomepizole, which targets ADH1A and ADH1B, is indicated as an antidote for ethylene glycol or methanol poisoning. Drugs targeting ERBB3 have been investigated for the treatment of cancer, including hepatocellular carcinoma. Glutamic acid, which targets FTCD, has been used for alcoholism control. Tissue expression analyses based on Human Protein Atlas and GTEx database showed that A1BG, ADH1A, ADH1B, C4BPA, F9 and FTCD, were mainly highly expressed in the liver. Single‐cell expression analysis confirmed that ADH1A, DCXR, A1BG, ACY1, F9, C4BPA, FTCD, MREG and ADH1B were predominantly expressed in hepatocytes, with minimal expression in other liver cell types. These results support the liver‐specific biological relevance of the identified proteins (Figure S7). After searching the functions of these proteins, we found that ADH1A, ADH1B, FBLN1 and IL11RA have clear associations with liver diseases, such as hepatocellular carcinoma and ALD. Although the precise roles of several identified proteins (e.g., NCAN, GKRP, DCXR) in liver disease pathogenesis have not been fully elucidated, their annotated biological functions suggest plausible mechanistic links to CLD development. Specifically, GKRP is a key regulator of glucokinase activity and hepatic glucose metabolism, DCXR participates in glucose metabolism and cellular osmoregulation, while NCAN is involved in cell adhesion and neural development. Additionally, other proteins exhibit liver‐relevant functions: AKR7A3 protects against the hepatocarcinogen aflatoxin B1, BMP1 modulates extracellular matrix formation and tissue repair, C4BPA regulates complement activation and F9 is critical for blood coagulation. These diverse functions in metabolic homeostasis, immune response, extracellular matrix remodelling and detoxification collectively support the biological relevance of these proteins as candidate biomarkers for liver diseases (Table S10). Pathway enrichment analysis revealed several key biological functions and disease associations closely linked to CLD pathophysiology (Figure S8). The most significant enrichment was alcohol dehydrogenase [NAD(P)+] activity, which explained the role of ADH1A and ADH1B in alcohol metabolism, a core mechanism in ALD. We also identified enrichment in liver cancer recurrence (M9911) and hepatocellular carcinoma (M34031), confirming the proteins' relevance to liver carcinogenesis. Additionally, enrichment of wound healing and immune response‐related gene sets (M5391, M6212) highlighted the proteins' involvement in liver fibrosis and hepatic inflammation, respectively.

### 3.7 Sixteen Proteins as Interventional Targets for Modifiable Factors

Table S11 presents associations between genetically predicted modifiable risk factors and CLDs. Higher genetically predicted BMI (OR = 1.69, 95% CI: 1.51–1.88) and WHR (OR = 1.81, 95% CI: 1.57–2.10) were significantly associated with an increased risk of MASLD. For ALD, significant positive associations were observed for genetically predicted lifetime smoking index (OR = 2.48, 95% CI: 1.65–3.74), problematic alcohol use (OR = 5.00, 95% CI: 2.78–9.01), and drinks per week (OR = 5.93, 95% CI: 2.71–12.99). Higher genetically predicted BMI (OR = 1.49, 95% CI: 1.28–1.75), WHR (OR = 1.72, 95% CI: 1.41–2.09) and fasting insulin (OR = 4.48, 95% CI: 2.39–8.39) were also linked to an increased risk of cirrhosis.

Table S12 presents the MR estimates for the causal effects of modifiable risk factors on plasma protein levels. Genetically predicted higher BMI and WHR were associated with increased levels of ACY1, ADH1A, AKR7A3, C4BPA, DCXR, F9, FTCD, IGDCC4, MREG, NAB1, GKRP and ADH1B (Figure 2D). The strongest positive association with BMI was observed for F9 (OR = 1.32, 95% CI: 1.25–1.40), while the association with WHR was most pronounced for ACY1 (OR = 1.38, 95% CI: 1.27–1.49). WHR also showed independent positive associations with A1BG (OR = 1.15, 95% CI: 1.08–1.22) and BMP1 (OR = 1.31, 95% CI: 1.21–1.42). In contrast, genetically predicted BMI (OR = 0.63, 95% CI: 0.59–0.66), WHR (OR = 0.79, 95% CI: 0.73–0.85) and leisure television watching (OR = 0.73, 95% CI: 0.62–0.85) were inversely associated with NCAN levels.

In MVMR analyses, the associations of BMI and WHR with MASLD risk were attenuated after adjusting for genetically predicted levels of 9 and 12 proteins, respectively (Figure 2E and Table S13). Similarly, the associations of BMI and WHR with cirrhosis risk were attenuated after adjusting for genetically predicted levels of ADH1A and DCXR. Mediation analysis revealed that a significant proportion of the BMI‐MASLD association was partially indirectly mediated through AKR7A3 (22.21%) and ADH1A (12.04%). Other proteins, including ACY1, DCXR, F9, FBLN1, FTCD, NAB1 and NCAN, each mediated less than 10% of this association. The WHR‐MASLD association was partially indirectly mediated through A1BG (7.99%), ACY1 (45.43%), ADH1A (34.12%), AKR7A3 (31.61%), BMP1 (13.67%), C4BPA (3.31%), DCXR (23.82%), F9 (15.69%), FTCD (22.78%), IGDCC4 (5.79%), NAB1 (9.99%) and NCAN (11.62%). Additionally, the positive associations of BMI and WHR with cirrhosis were partially indirectly mediated through ADH1A (9.05% for BMI; 7.44% for WHR) and DCXR (9.90% for BMI; 1.24% for WHR) (Table S13).

### 3.8 Baseline Characteristics of Prospective Cohort

The basic characteristics of the study populations are presented in Table S14. In Subgroups 1–4, the incident cases of outcomes (total participants) were 101 (608), 151 (34 316), 2 (254) and 18 (34 670), respectively. Among the 34 778 general participants, the mean (SD) age was 57.14 (8.10) years, with 45.7% (15 896) being male. During a median follow‐up of 15.2 years, there were 491 incident cases of MASLD, 65 of ALD, 211 of cirrhosis, 17 of liver cancer, 69 of ascites, 4 of variceal bleeding, 170 of SBP, 97 of hepatic encephalopathy and 37 of liver failure (Figure S1). In the training set (*n* = 24 345), there were 469 incident cases of CLD and 155 of CHE. In the validation set (*n* = 10 433), there were 207 incident cases of CLD and 69 of CHE.

### 3.9 Five Plasma Proteins Associated With CLD Progression

**FIGURE 3:**
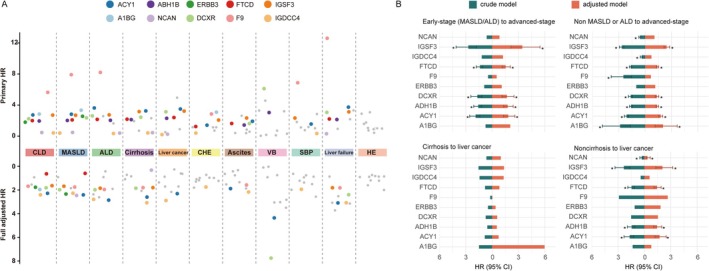
Associations between plasma proteins and chronic liver diseases, composite hepatic events and disease progression. (A) Associations between proteins and chronic liver disease and composite hepatic events. (B) Associations between proteins and progression of liver diseases. Full adjusted model was adjusted for gender, age, Townsend deprivation index, education, smoking status, alcohol consumption, body mass index, physical activity, healthy diet, waist circumference, hyperlipidemia and hypertension. A1BG, Alpha‐1B‐glycoprotein; ACY1, Aminoacylase‐1; ADH1B, Alcohol dehydrogenase 1B; ALD, alcoholic liver diseases; CHE, composite hepatic events; CI, confidence interval; CLD, chronic liver disease; DCXR, Dicarbonyl and L‐xylulose reductase; ERBB3, Erb‐B2 Receptor Tyrosine Kinase 3; F9, Coagulation Factor IX; FTCD, formimidoyltransferase cyclodeaminase; HE, hepatic encephalopathy; HR, hazard ratio; IGDCC4, immunoglobulin superfamily DCC subclass member 4; IGSF3, immunoglobulin superfamily member 3; MASLD, metabolic dysfunction‐associated steatotic liver disease; NCAN, Neurocan core protein; SBP, spontaneous bacterial peritonitis; VB, variceal bleeding.

Figure 3A and Table S15 show that these 10 proteins were all associated with at least one CLD or CHE. In the fully adjusted model, most proteins, including ACY1, DCXR, FTCD, IGSF3 and ADH1B, were strongly and positively associated with CLD, MASLD, ALD, cirrhosis and liver cancer (all *p* < 0.05). Notably, higher plasma levels of ACY1, DCXR, IGSF3 and ADH1B were consistently associated with an increased risk of major liver‐related outcomes, whereas IGDCC4 showed inverse associations with multiple liver disease endpoints.

Figure 3B and Table S16 illustrate the independent associations of these proteins with CLD progression. After adjustment for potential covariates, higher levels of ACY1 (HR = 1.83, 95% CI: 1.29–2.59), DCXR (HR = 1.68, 95% CI: 1.13–2.49), FTCD (HR = 1.54, 95% CI: 1.15–2.07), IGSF3 (HR = 3.43, 95% CI: 2.15–5.45) and ADH1B (HR = 1.77, 95% CI: 1.31–2.39) were positively associated with an increased risk of early‐stage CLD (MASLD or ALD) progressing to advanced liver disease (cirrhosis or liver cancer). Similarly, the levels of ACY1, FTCD, IGSF3 and ADH1B were associated with liver cancer progression in individuals without cirrhosis, with HRs of 1.73 (95% CI: 1.20–2.48) for ACY1, 1.44 (95% CI: 1.09–1.90) for FTCD, 2.05 (95% CI: 1.30–3.23) for IGSF3 and 1.44 (95% CI: 1.05–1.97) for ADH1B. However, no significant association was observed between these proteins and liver cancer progression in cirrhosis patients. The interaction between ACY1 and IGSF3 exerted a significant synergistic promotional effect on CLD risk (HR = 2.05, 95% CI: 1.13–3.73), whereas the interaction of ERBB3 and ADH1B (HR = 0.16, 95% CI: 0.04–0.71), FTCD and ADH1B (HR = 0.66, 95% CI: 0.44–0.97), as well as IGSF3 and NCAN (HR = 0.63, 95% CI: 0.29–0.96) all showed significant antagonistic effects on CLD risk (Table S17).

### 3.10 The Predictive Value of Identified Plasma Proteins for CLDs and CHEs

**FIGURE 4:**
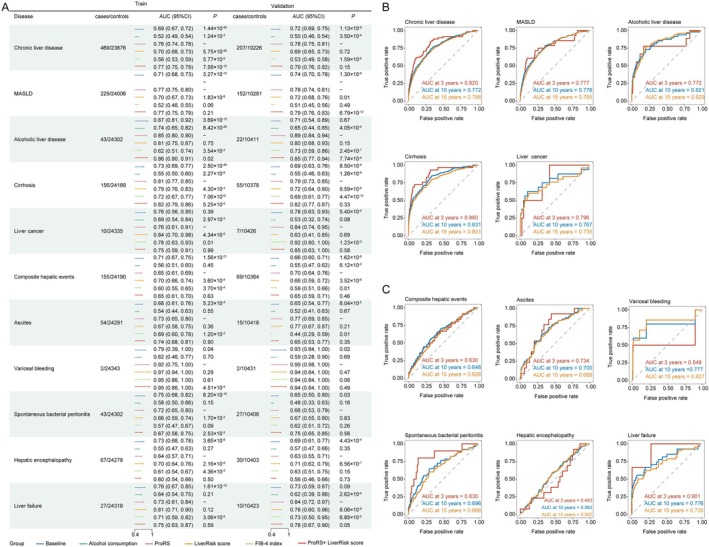
Predictive value of the ProRS and other clinical predictor sets. (A) Performance of the baseline characteristics, alcohol consumption, ProRS, LiverRisk score, FIB‐4 index and the combined model in the prediction of liver diseases. (B) Time ROC curves of ProRS for chronic liver diseases. (C) Time ROC curves of ProRS for composite hepatic events. AUC, area under the curve; CI, confidence interval; FIB‐4 index, fibrosis‐4 index; MASLD, metabolic dysfunction‐associated steatotic liver disease; ProRS, proteomic risk score.

We evaluated the predictive performance of baseline characteristics, alcohol consumption, ProRS, LiverRisk score and FIB‐4 index for various liver disease outcomes. As shown in Figure 4A, the ProRS model demonstrated the best performance for predicting CLD (C‐index = 0.78; 95% CI: 0.75–0.81), MASLD (C‐index = 0.78; 95% CI: 0.74–0.81), ALD (C‐index = 0.89; 95% CI: 0.84–0.94), cirrhosis (C‐index = 0.79; 95% CI: 0.73–0.85), CHE (C‐index = 0.70; 95% CI: 0.64–0.76) and liver failure (C‐index = 0.84; 95% CI: 0.72–0.97). However, the predictive performance of ProRS was either inferior to or comparable with that of the LiverRisk score for ascites, SBP and hepatic encephalopathy. Notably, the FIB‐4 index achieved the best performance for liver cancer prediction (C‐index = 0.92; 95% CI: 0.80–1.00).

When adding ProRS into the LiverRisk score models, an enhancement in predictive capability was observed across CLD (C‐index = 0.79; 95% CI: 0.76–0.82 for ComRS and C‐index = 0.69; 95% CI: 0.65–0.73 for LiverRisk score), MASLD (C‐index = 0.79; 95% CI: 0.76–0.83 for ComRS and C‐index = 0.72; 95% CI: 0.68–0.76 for LiverRisk score), ALD (C‐index = 0.85; 95% CI: 0.77–0.94 for ComRS and C‐index = 0.80; 95% CI: 0.68–0.93 for LiverRisk score), cirrhosis (C‐index = 0.82; 95% CI: 0.77–0.87 for ComRS and C‐index = 0.72; 95% CI: 0.64–0.80 for LiverRisk score), liver cancer (C‐index = 0.85; 95% CI: 0.63–1.00 for ComRS and C‐index = 0.63; 95% CI: 0.41–0.85 for LiverRisk score) and SBP (C‐index = 0.75; 95% CI: 0.65–0.85 for ComRS and C‐index = 0.67; 95% CI: 0.55–0.80 for LiverRisk score) compared to the LiverRisk score alone. However, the combined model did not significantly outperform ProRS alone. Adding ProRS to the LiverRisk score significantly improved risk stratification for multiple liver‐related outcomes at 3 years. In the validation cohort, notable improvements were observed for CLD (NRI = 0.3569, *p* < 0.0001; IDI = 0.0046, *p* < 0.0001), ALD (NRI = 0.5169, *p* < 0.0001; IDI = 0.0074, *p* < 0.0001), cirrhosis (NRI = 0.9222, *p* < 0.0001; IDI = 0.0092, *p* < 0.0001) and CHEs (NRI = 0.3431, *p* = 0.012; IDI = 0.0003, *p* < 0.0001) (Table S18). Moreover, ProRS and ComRS achieved similar and superior net benefit compared with other models in the training set. In the validation set, ProRS performed better in CLD, MASLD and cirrhosis, while ComRS was superior in ALD. (Figure S9). Subgroup analyses were conducted according to sex, age and BMI. ProRS demonstrated stable and comparable predictive performance across these subgroups in both training and validation datasets, with no evidence of significant heterogeneity (Table S19).

We investigated the predictive abilities of ProRS across different time windows. For most endpoints (Figure 4B,C), ProRS achieved the highest AUC when forecasting outcomes occurring within 3 years (C‐index = 0.820 for CLD, 0.777 for MASLD, 0.880 for cirrhosis, 0.796 for liver cancer, 0.734 for ascites, 0.830 for SBP and 0.901 for liver failure), suggesting the pivotal role of plasma proteomics in detecting near‐term risks. For ALD, the over 10‐year model showed the highest AUC with C‐index of 0.829. However, for hepatic encephalopathy, ProRS showed low AUC across different time windows. Calibration curves indicated good concordance between the observed and predicted rates (Figure S10). The Kaplan–Meier cumulative incidence curves showed distinctive paths between the tertiles stratified by ProRS. We found a strong association between ProRS groups and the probability of developing CLD, MASLD, ALD, cirrhosis and liver cancer, CHE, ascites, SBP, liver failure. Participants in moderate‐risk groups and high‐risk groups had a progressively higher probability of developing CLDs or CHEs at 15.2 years of follow‐up compared with those in the low‐risk group (Log‐rank *p* < 0.001) (Figures S11 and S12). Furthermore, the ProRS high‐risk group corresponded to median 10‐year absolute risks of 1.01% for CLDs and 0.44% for CHEs, respectively. Despite the low absolute risk, the high‐risk ProRS group consistently exhibited a modest but meaningful elevation in long‐term absolute risk compared with the low‐risk group for all liver‐related outcomes (Table S20).

## 4 Discussion

Our study applied an integrative approach to evaluate the causal impact of thousands of plasma proteins on four major CLDs. Using *cis*‐pQTLs and all pQTLs, we identified 3 and 19 unique proteins, respectively. These identified proteins could be modulated by pharmacological intervention or modifiable factors such as BMI, WHR, glycated haemoglobin, T2D and leisure television watching. Further analysis using proteomic profiling revealed 5 proteins including IGSF3, FTCD, DCXR, ADH1B and ACY1, all of which were involved in the progression from early‐stage CLD to cirrhosis or liver cancer. Additionally, we developed a prediction model based on these proteins, which effectively identified individuals at risk for CLDs and CHEs.

Our research discovered key proteins related to the progression of liver diseases, including IGSF3, FTCD, DCXR, ADH1B and ACY1. ADH1B and FTCD were specifically expressed in the liver. These proteins were mainly located in the cytoplasm (IGSF3, DCXR, ADH1B), nucleoplasm (FTCD) and vesicles (ACY1). These proteins offered insights into the underlying mechanisms and potential therapeutic targets for CLD. Mechanistically, IGSF3 has been shown to significantly enhance cell adhesion to fibronectin [19], a function linked to its regulation of cytoskeletal dynamics [20]. Notably, lymphoblasts deficient in IGSF3 exhibit increased survival and cell adhesion capacity, which may amplify hepatic inflammatory responses [21]. Therefore, IGSF3 may play a key role in maintaining the structural and functional integrity of liver tissue. FTCD is a candidate suppressor gene for hepatocellular carcinoma [22, 23], and glutamic acid targeting FTCD has been used for alcoholism control. DCXR, previously recognised as an oncogene in breast cancer that modulates tumour progression, exerts its pro‐pathogenic effects through the regulation of glycolysis, cell cycle progression and proliferation [24]. Additionally, DCXR generates reactive oxygen species (ROS) via redox reactions [25], reduces intracellular oxygen concentrations, and depletes nicotinamide adenine dinucleotide and triphosphopyridine nucleotide pools [26], ultimately contributing to hepatic tissue damage. For ADH1B, a case–control study demonstrated that carriers of the rs1229984 variant have a lower propensity for habitual alcohol consumption [27], though this variant is rare in European populations [28]; conversely, individuals with the wild‐type *ADH1B* rs1229984 genotype show a higher inclination towards alcohol intake. As a member of the ADH gene cluster, ADH1A shares analogous functions and mechanisms with ADH1B [29]. ACY1 levels are elevated in fully differentiated adipocytes [30], and its activity is important for the intracellular transport of long‐chain fatty acids and is involved in hepatic triglyceride formation [30], contributing to MASLD formation.

Beyond the above CLD progression‐related proteins, our MVMR analysis further revealed that ACY1, DCXR, FTCD, along with AKR7A3, ADH1A, BMP1 and F9, exert partial indirect mediating effects on the associations between obesity and CLD. These mediating roles are strongly supported by the proteins' well‐characterised biological functions and growing evidence in liver disease pathogenesis. ADH1A, C4BPA, F9 and FTCD were specifically expressed in the liver. Specifically, AKR7A3 is significantly correlated with plasma levels of low‐density lipoprotein and total cholesterol [31]. AKR7A3 along with AKR7B3 was consistently increased at both mRNA and protein levels from early to late tumour stages in the rat experimental hepatocellular carcinoma [32]. Previous observational studies reported the involvement of AKR1 and AKR7 series in the occurrence of breast, lung, liver and bowel cancers. The beneficial effects of lower aldoketo reductase in reducing lipid peroxides and proinflammatory cytokines may contribute to the improvement of liver fibrosis. In addition, its protective effect against oxidative stress is reduced due to increased aldo‐keto reductase activity and increased advanced glycation end products [33, 34]. For BMP1, Grgurevic et al. [35] reported that this protein accelerates the cleavage of type I procollagen, driving collagen deposition in cirrhotic livers; additionally, BMP1‐3 has been shown to promote renal fibrosis by enhancing extracellular matrix accumulation in a rat model of chronic kidney disease [36], highlighting its conserved role in fibrotic pathogenesis across tissues. F9 (FIX), a vitamin K‐dependent coagulation protein exclusively synthesised in the liver, is associated with T2D [37], obesity [38] and elevated circulating IL‐6 concentrations [39]. These conditions are common in patients with MASLD [39, 40, 41, 42]. Kotronen et al. demonstrated that plasma activities of F9 are elevated in MASLD patients and correlate with insulin resistance; in obese co‐twins, increased levels of fibrinogen, F9 and plasminogen activator inhibitor‐1 are strongly associated with adiposity, inflammation and insulin resistance [43].

Pathway enrichment analysis further strengthened the biological plausibility of these proteins to core CLD mechanisms. The significant enrichment of alcohol dehydrogenase [NAD(P)+] activity supported the role of ADH1A and ADH1B in alcohol metabolism. The enrichment of liver cancer‐related gene sets and immune response signatures supported the proteins' involvement in liver carcinogenesis and inflammatory progression, complementing our PPI network analysis. These findings highlighted the multi‐factor nature of CLD progression and underscored the potential of these proteins as biomarkers and therapeutic targets. Understanding their roles in regulating the impact of lifestyle factors on the risk of liver diseases can inform targeted interventions and improve clinical management strategies.

We also discovered five proteins associated with at least one kind of CLD or CHE, including NCAN, IGDCC4, ERBB3, F9, A1BG. NCAN is primarily expressed in the liver and nervous system, where it participates in cell migration and adhesion [44]. The liver is highly innervated by both sympathetic and parasympathetic nerves [45]. Bruinstroop et al. found significantly higher serum triglyceride levels after meals in parasympathetic or sympathetic denervated rats compared to sham‐operated animals [46]. NCAN may increase liver very low‐density lipoprotein triglyceride secretion through vagal action [44]. IGDCC4 is associated with tissue fibrosis and inflammatory cell infiltration, leading to damage of the bile duct and liver parenchyma, and subsequently causes liver cirrhosis [47]. ERBB3 is involved in liver fibrotic diseases, and its downregulation can also inhibit the proliferation of liver cancer cells and induce apoptosis [48]. F9 (FIX) has been associated with obesity [38] and T2D [37]. Kotronen et al. [42] found that FIX activity increased in MASLD patients and was associated with insulin resistance. This is consistent with our findings. A1BG is mainly expressed in the liver and is differentially expressed in liver cancer cell lines [49].

Our study evaluated the predictive performance of baseline characteristics, alcohol consumption, ProRS and the LiverRisk score for various liver disease outcomes. ProRS emerged as a promising tool for early risk stratification and prediction of CLDs and CHEs. Our findings indicated that ProRS demonstrated superior predictive performance for all types of CLDs when compared to established clinical indicators, with C‐index values consistently above 0.78. Furthermore, this model exhibited strong predictive accuracy for CHE, particularly for ascites, SBP and liver failure. Notably, ProRS achieved ideal performance for most endpoints when used alone, with predictive capabilities that were comparable or nearly equivalent to models that combine ProRS with the LiverRisk score. This highlighted the efficiency of ProRS in risk stratification, potentially reducing the need for more complex, multidimensional data collection. The model's simplicity, incorporating only age, sex and levels of 10 proteins, alleviated the burden of data collection. The superior predictive performance of ProRS across various liver disease outcomes highlighted its potential as a valuable tool for early risk detection and stratification. The ability to predict multiple disease outcomes from a single proteomic profile suggested that ProRS could serve as a comprehensive risk assessment tool in clinical practice.

From a translational perspective, the present findings advanced clinical practice and drug development in several meaningful ways. First, the ProRS complemented conventional liver function tests such as ALT and AST by capturing pre‐clinical, subclinical proteomic changes that preceded overt liver enzyme elevation, thereby enabling earlier risk detection and intervention before irreversible liver damage occurred. Second, the 12 druggable proteins identified in this study, including ERBB3, ADH1A, DCXR and ACY1, addressed critical gaps in current CLD therapeutics, particularly the lack of targeted agents for MASLD progression and liver fibrosis. These proteins represented novel, actionable molecular targets for pharmacological modulation, offering new avenues for disease‐modifying therapy. Third, our MR and mediation results supported that modifiable factors including obesity and alcohol consumption might act through the identified proteins to influence CLD risk, highlighting the potential value of lifestyle interventions for liver disease prevention. Collectively, these results strengthened the clinical translatability of proteomic biomarkers and supported their integration into routine risk assessment and precision intervention strategies.

Future research can focus on validating these findings in multi‐center cohorts to confirm the applicability of ProRS across diverse populations. Delving into the intricate pathways by which specific proteins impact the progression and outcomes of liver diseases can provide a deeper understanding of the disease pathogenesis. Moreover, combining proteomic information with data from other omics fields, such as genomics and metabolomics, has the potential to significantly boost the precision of risk prediction models and provide a more comprehensive and integrated perspective on liver disease susceptibility.

The strengths of this study are that we systematically examined the associations between plasma protein biomarkers and CLD risk with the advantages of rich proteome coverage, large sample sizes and avoidance of interference from reverse causality. Second, the consistency of the results enhances the reliability of the research results. Third, evidence from PPI and druggability evaluations provided important new ideas for precise targeted treatment strategies and comprehensive clinical management strategies of potential CLD pathways. Moreover, European individuals' analysis reduced demographic stratification bias. This ensures the model's feasibility and acceptability. There are several limitations that should be recognised. We focused on plasma proteins, whose levels may differ from those in liver tissues or hepatocytes. Second, there are some proteins without available data on measured content, which may affect the prediction ability of our model. Third, we distinguished multiple types of CLDs and CHEs, resulting in a small number of cases. The ProRS demonstrated a high C‐index of 0.99 for predicting ascites in the validation set. However, this result should be interpreted with caution due to the very small number of cases (*n* = 2) in the validation set. The limited sample size may introduce substantial random variability and potential overfitting, thus questioning the reliability of this high C‐index. Further validation with a larger sample size is warranted to confirm the predictive performance of ProRS for ascites. Fourth, although the ProRS demonstrated good discrimination, the absolute risk differences between risk groups were small in this general population cohort. Future research should evaluate ProRS performance in high‐risk populations to assess its clinical utility where absolute risk stratification is more pronounced. In terms of model validation, we only conducted internal verification within the UK Biobank cohort, which limits the external validity of the ProRS model. Additionally, the entire derivation and validation samples are confined to individuals of European ancestry, so the generalisability of the study results to other populations may be restricted. To address these limitations, we plan to validate the model in multi‐center, cross‐ethnic cohorts in future research. This will help comprehensively evaluate the external applicability and cross‐ethnic robustness of the ProRS model and further refine the model to enhance its utility for global clinical practice.

## 5 Conclusions

In conclusion, this study identified many circulating proteins causally associated with liver‐related diseases. The constructed risk prediction model showed a good discriminatory ability for CLD and CHE, which provided a new strategy for early risk screening and accurate diagnosis and treatment.

## Author Contributions

Christos S. Mantzoros, Xue Li, Juan Lu, Shuai Yuan and Xinxuan Li contributed to the study conceptualisation and design. Material preparation, data curation and formal analysis were performed by Xinxuan Li and Jing Sun. Validation was performed by Jianhui Zhao and Fangyuan Jiang. Visualisation was performed by Meng Zhang and Hao Wu. The original draft of the manuscript was written by Xinxuan Li and all authors commented on previous versions of the manuscript. All authors read and approved the final manuscript. The authorship order among the co‐first authors was determined to reflect their substantial contribution to the study, while acknowledging varying degrees of involvement in the collaborative work.

## Funding

This work was supported by the National Natural Science Foundation of China (grant no. 82204019) and the Natural Science Fund for Distinguished Young Scholars of Zhejiang Province (LR22H260001) to X.L.

## Ethics Statement

These studies included in the original genome‐wide association studies (GWASs) had obtained the necessary ethical approvals from the relevant committees.

## Consent

Written informed consent was obtained from all individuals involved in these studies.

## Conflicts of Interest

The authors declare no conflicts of interest.

## Supporting information


**Table S1:** GWAS resources for modifiable risk factors.
**Table S2:** ICD coding for liver‐related diseases.
**Table S3:** Detailed missing status of covariates.
**Table S4:** Genetic instruments for plasma proteome.
**Table S5:** Summary results from Mendelian randomization (MR), colocalization and SMR for 3 proteome‐wide MR identified proteins using *cis*‐pQTLs.
**Table S6:** Summary results from Mendelian randomization (MR), colocalization and SMR for 19 proteome‐wide MR identified proteins using all the pQTLs.
**Table S7:** *Cis*‐only Mendelian randomization results after covariate adjustment.
**Table S8:** *Cis*+*trans* Mendelian randomization results after covariate adjustment.
**Table S9:** Druggability of proteins potentially causally associated with liver diseases.
**Table S10:** Functions of identified proteins.
**Table S11:** Results of Mendelian randomization analysis from main analysis and alternative methods between modifiable risk factors and CLDs.
**Table S12:** Results of Mendelian randomization analysis from main analysis and alternative methods between modifiable risk factors and identified proteins.
**Table S13:** Associations of body mass index and waist‐hip ratio with CLDs after adjusting for proteins and proportion mediated.
**Table S14:** Baseline characteristics of participants.
**Table S15:** Associations between 10 proteins and liver diseases.
**Table S16:** Associations between proteins and progression of chronic liver disease.
**Table S17:** Protein–protein interaction analysis of 10 proteins in chronic liver disease risk.
**Table S18:** Results of net reclassification improvement and integrated discrimination improvement.
**Table S19:** Results of subgroup analyses.
**Table S20:** 10‐year absolute risk of liver‐related outcomes across ProRS risk groups.

**Figure S1:** Flowchart for the selection of study participants.
**Figure S2:** Regional association plot for colocalization analysis of proteins identified by *cis*‐MR with CLDs risk.
**Figure S3:** Regional association plot for colocalization analysis of proteins identified by all‐MR with MASLD risk.
**Figure S4:** Regional association plot for colocalization analysis of proteins identified by all‐MR with ALD risk.
**Figure S5:** Regional association plot for colocalization analysis of proteins identified by all‐MR with cirrhosis risk.
**Figure S6:** The correlation heat map of covariates.
**Figure S7:** Results of single‐cell expression analysis.
**Figure S8:** Results of pathway enrichment analysis.
**Figure S9:** Decision curve analysis in the (A) training and (B) validation datasets.
**Figure S10:** Calibration plots of proteomic risk score for chronic liver disease and composite hepatic event.
**Figure S11:** The cumulative incidence curves for chronic liver disease stratified by proteomic risk score tertiles.
**Figure S12:** The cumulative incidence curves for composite hepatic event stratified by proteomic risk score tertiles.

## Data Availability

All data used in this study are in the public domain. The results of this study are included in this published article and its Supporting Information.
