# Supplementary material for: Proteomic Profiling of Plasma to Uncover Novel Intervention Targets and Prognostic Biomarkers for Chronic Liver Diseases
Source: Diabetes Obes Metab. 2026 Mar 26;28(6):5029–42. doi: 10.1111/dom.70696 (PMC13146168; doi:10.1111/dom.70696)
Supplement: Supplementary file 1 — Table S1: GWAS resources for modifiable risk factors. Table S2: ICD coding for liver‐related diseases. Table S3: Detailed missing status of covariates. Table S4: Genetic instruments for plasma proteome. Table S5: Summary results from Mendelian randomization (MR), colocalization and SMR for 3 proteome‐wide MR identified proteins using cis‐pQTLs. Table S6: Summary results from Mendelian randomization (MR), colocalization and SMR for 19 proteome‐wide MR identified proteins using all the pQTLs. Table S7: Cis‐only Mendelian randomization results after covariate adjustment. Table S8: Cis+trans Mendelian randomization results after covariate adjustment. Table S9: Druggability of proteins potentially causally associated with liver diseases. Table S10: Functions of identified proteins. Table S11: Results of Mendelian randomization analysis from main analysis and alternative methods between modifiable risk factors and CLDs. Table S12: Results of Mendelian randomization analysis from main analysis and alternative methods between modifiable risk factors and identified proteins. Table S13: Associations of body mass index and waist‐hip ratio with CLDs after adjusting for proteins and proportion mediated. Table S14: Baseline characteristics of participants. Table S15: Associations between 10 proteins and liver diseases. Table S16: Associations between proteins and progression of chronic liver disease. Table S17: Protein–protein interaction analysis of 10 proteins in chronic liver disease risk. Table S18: Results of net reclassification improvement and integrated discrimination improvement. Table S19: Results of subgroup analyses. Table S20: 10‐year absolute risk of liver‐related outcomes across ProRS risk groups. [file DOM-28-5029-s001.docx]

**Supplementary Tables**

**Supplementary Table 1. GWAS resources for modifiable risk factors.**

**Supplementary Table 2. ICD coding for liver-related diseases.**

**Supplementary Table 3. Detailed missing status of covariates.**

**Supplementary Table 4. Genetic instruments for plasma proteome.**

**Supplementary Table 5. Summary results from Mendelian randomization (MR), Colocalization, and SMR for 3 proteome-wide MR identified proteins using *cis*-pQTLs.**

**Supplementary Table 6. Summary results from Mendelian randomization (MR), Colocalization, and SMR for 19 proteome-wide MR identified proteins using all the pQTLs.**

**Supplementary Table 7. *Cis*-only Mendelian Randomization results after covariate adjustment.**

**Supplementary Table 8. *Cis*+*trans* Mendelian Randomization results after covariate adjustment.**

**Supplementary Table 9. Druggability of proteins potentially causally associated with liver diseases.**

**Supplementary Table 10. Functions of identified proteins.**

**Supplementary Table 11. Results of Mendelian Randomization analysis from main analysis and alternative methods between modifiable risk factors and CLDs.**

**Supplementary Table 12. Results of Mendelian Randomization analysis from main analysis and alternative methods between modifiable risk factors and identified proteins.**

**Supplementary Table 13. Associations of body mass index and waist-hip ratio with CLDs after adjusting for proteins and proportion mediated.**

**Supplementary Table 14. Baseline characteristics of participants.**

**Supplementary Table 15. Associations between 10 proteins and liver diseases.**

**Supplementary Table 16. Associations between proteins and progression of chronic liver disease.**

**Supplementary Table 17. Protein-protein interaction analysis of 10 proteins in chronic liver disease risk.**

**Supplementary Table 18. Results of net reclassification improvement and integrated discrimination improvement.**

**Supplementary Table 19. Results of subgroup analyses.**

**Supplementary Table 20. 10-year absolute risk of liver-related outcomes across ProRS risk groups.**

**Supplementary Table 1. GWAS resources for modifiable risk factors.**

| **Modifiable risk factors** | **Class** | **PMID** | **Year** | **IVs** | **N** | **Ancestry** |
| --- | --- | --- | --- | --- | --- | --- |
| Body mass index | Obesity | 30239722 | 2018 | 538 | 694,649 | European |
| Body fat percentage | Obesity | 26833246 | 2016 | 9 | 23,469 | European |
| Waist-hip ratio | Obesity | 30239722 | 2018 | 356 | 697,734 | European |
| Waist circumference | Obesity | 34017140 | 2021 | 87 | 407,661 | European |
| Glycated hemoglobin | Obesity | 34059833 | 2021 | 58 | 146,806 | European |
| Type 2 diabetes | Obesity | 22885922 | 2012 | 39 | 69,033 | European |
| Fasting glucose | Obesity | 34059833 | 2021 | 57 | 200,622 | European |
| Fasting insulin | Obesity | 34059833 | 2021 | 33 | 151,013 | European |
| Proinsulin | Obesity | 36693378 | 2023 | 24 | 45,861 | European |
| Two-hour glucose | Obesity | 34059833 | 2021 | 12 | 63,396 | European |
| Age at initiation of smoking | Lifestyle | 30643251 | 2019 | 9 | 341,427 | European |
| Cigarettes per Day | Lifestyle | 30643251 | 2019 | 35 | 377,334 | European |
| Lifetime smoking index | Lifestyle | 31689377 | 2019 | 120 | 462,690 | European |
| Smoking cessation | Lifestyle | 30643251 | 2019 | 15 | 547,219 | European |
| Smoking initiation | Lifestyle | 30643251 | 2019 | 193 | 547,219 | European |
| Coffee consumption | Lifestyle | 31046077 | 2019 | 29 | 39,924 | European |
| Drinks per week | Lifestyle | 32451486 | 2020 | 71 | 972,915 | European |
| Problematic alcohol use | Lifestyle | 32451486 | 2020 | 58 | 972,915 | European |
| Tea consumption | Lifestyle | 31046077 | 2019 | 12 | 336,898 | European |
| Leisure computer use | Lifestyle | 32317632 | 2020 | 21 | 408,815 | European |
| Leisure television watching | Lifestyle | 32317632 | 2020 | 86 | 408,815 | European |
| Spent driving | Lifestyle | 32317632 | 2020 | 4 | 408,815 | European |
| Insomnia | Lifestyle | 30804565 | 2019 | 153 | 1,331,010 | European |
| Sleep duration | Lifestyle | 30846698 | 2019 | 58 | 446,118 | European |
| Moderate-to-vigorous intensity physical activity | Lifestyle | 36071172 | 2022 | 19 | 608,595 | European |
| Dietary pattern | Dietary | 32193382 | 2020 | 99 | 449,210 | European |
| Bowls of cereal per week | Dietary | 32193382 | 2020 | 13 | 373,443 | European |
| Glasses of water per day | Dietary | 32193382 | 2020 | 20 | 445,965 | European |
| Glasses of milk intake per week | Dietary | 29537719 | 2018 | 1 | 73,715 | European |
| Overall beef intake | Dietary | 32193382 | 2020 | 2 | 447,441 | European |
| Overall cheese intake | Dietary | 32193382 | 2020 | 24 | 438,453 | European |
| Overall lamb/mutton intake | Dietary | 32193382 | 2020 | 8 | 446,443 | European |
| Overall non-oily fish intake | Dietary | 32193382 | 2020 | 2 | 447,289 | European |
| Overall oily fish intake | Dietary | 32193382 | 2020 | 22 | 446,854 | European |
| Overall pork intake | Dietary | 32193382 | 2020 | 5 | 446,607 | European |
| Overall poultry intake | Dietary | 32193382 | 2020 | 3 | 448,210 | European |
| Overall processed meat intake | Dietary | 32193382 | 2020 | 6 | 448,303 | European |
| Relative carbohydrate intake | Dietary | 34426670 | 2021 | 5 | 282,271 | European |
| Relative fat intake | Dietary | 34426670 | 2021 | 7 | 282,271 | European |
| Relative protein intake | Dietary | 34426670 | 2021 | 7 | 282,271 | European |
| Pieces of dried fruit per day | Dietary | 32193382 | 2020 | 9 | 444,741 | European |
| Pieces of fresh fruit per day | Dietary | 32193382 | 2020 | 37 | 447,401 | European |
| Processed meat consumption | Dietary | 32066663 | 2020 | 4 | 335,576 | European |
| Slices of bread per week | Dietary | 32193382 | 2020 | 10 | 444,230 | European |
| Sweet beverage consumption | Dietary | 31046077 | 2019 | 3 | 85,852 | European |
| Tablespoons of cooked vegetables per day | Dietary | 32193382 | 2020 | 7 | 444,190 | European |
| Tablespoons of raw vegetables per day | Dietary | 32193382 | 2020 | 7 | 443,633 | European |
| Abbreviations: PMID, PubMed unique identifier; IVs, instrumental variables. | | | |  |  |  |

**Supplementary Table 2. ICD coding for liver-related diseases.**

| **Diseases** | **ICD code** | | **Description** | **Detail** |
| --- | --- | --- | --- | --- |
| Mild MASLD | ICD-9 | 571.8 | Other chronic nonalcoholic liver disease | Plus one of the following conditions:  1.type 2 diabetes mellitus,  2.overweight/obesity (body mass index  ≥ 25 kg/m2),or 3.metabolic abnormality including any of the two:  insulin resistance (not collected in the UK Biobank study),  prediabetes (glucose ≥ 100 mg/dl or glycated hemoglobin ≥ 5.7%),  low high-density lipoprotein cholesterol (< 1.03 mmol/L for males; < 1.29 mmol/L for females),  hypertriglyceridemia (≥ 1.7 mmol/L),  hypertension (≥ 130/85 mmHg or use of antihypertensive medication), and  increased waist circumference (≥ 102 cm for males; ≥ 88 cm for females). The definition of MASLD further excluded excess alcohol consumption (male with >30 g/day ethanol intake and female with >20 g/day ethanol intake) and viral hepatitis infection. |
|  |  | 571.9 | Unspecified chronic liver disease without mention of alcohol |  |
|  | ICD-10 | K75.8 | Nonalcoholic steatohepatitis |  |
|  |  | K76.0 | Fatty (change of) liver, not elsewhere classified |  |
| Mild alcoholic liver disease | ICD-9 | 571.0 | Alcoholic fatty liver |  |
|  |  | 571.1 | Alcoholic hepatitis |  |
|  |  | 571.3 | Alcohol liver damage, unspecified |  |
|  | ICD-10 | K70.0 | Alcoholic fatty liver |  |
|  |  | K70.1 | Alcoholic hepatitis |  |
|  |  | K70.9 | Alcoholic liver disease, unspecified |  |
| Cirrhosis | ICD-9 | 571.5 | Cirrhosis of liver without mention of alcohol |  |
|  |  | 571.6 | Biliary cirrhosis |  |
|  |  | 571.2 | Alcohol cirrhosis liver |  |
|  | ICD-10 | K70.2 | Alcoholic fibrosis and sclerosis of the liver |  |
|  |  | K70.3 | Alcoholic cirrhosis of the liver |  |
|  |  | K70.4 | Alcoholic hepatic failure |  |
|  |  | K74.0 | Hepatic fibrosis |  |
|  |  | K74.1 | Hepatic sclerosis |  |
|  |  | K74.2 | Hepatic fibrosis with hepatic sclerosis |  |
|  |  | K74.3 | Primary biliary cirrhosis |  |
|  |  | K74.4 | Secondary biliary cirrhosis |  |
|  |  | K74.5 | Biliary cirrhosis, unspecified |  |
|  |  | K74.6 | Other and unspecified cirrhosis of liver |  |
|  |  | K76.6 | Portal hypertension |  |
|  |  | I85 | Oesophageal varices |  |
| Liver cancer | ICD-9 | 155.1 | Malignant neoplasm of liver, primary |  |
|  | ICD-10 | C22.0 | Liver cell carcinoma |  |
|  |  | C22.1 | Intrahepatic bile duct carcinoma |  |
|  |  | C22.2 | Hepatoblastoma |  |
|  |  | C22.3 | Angiosarcoma of liver |  |
|  |  | C22.4 | Other sarcomas of liver |  |
|  |  | C22.7 | Other specified carcinomas of liver |  |
|  |  | C22.8 | Malignant neoplasm of liver, primary, unspecified as to type |  |
|  |  | C22.9 | Malignant neoplasm of liver, not specified as primary or secondary | |
| Viral hepatitis | ICD-9 | 5714 | Chronic hepatitis |  |
|  |  | 0701 | Viral hepatitis a without mention of hepatic coma |  |
|  |  | 0703 | Viral hepatitis b without mention of hepatic coma |  |
|  |  | 0705 | Other specified viral hepatitis without mention of hepatic coma |  |
|  |  | 0709 | Unspecified viral hepatitis without mention of hepatic coma |  |
|  | ICD-10 | B15.0 | Hepatitis A with hepatic coma |  |
|  |  | B15.9 | Hepatitis A without hepatic coma |  |
|  |  | B16.0 | Acute hepatitis B with delta-agent with hepatic coma |  |
|  |  | B16.1 | Acute hepatitis B with delta-agent without hepatic coma |  |
|  |  | B16.2 | Acute hepatitis B without delta-agent with hepatic coma |  |
|  |  | B16.9 | Acute hepatitis B without delta-agent and without hepatic coma |  |
|  |  | B17.0 | Acute delta-(super) infection of hepatitis B carrier |  |
|  |  | B17.1 | Acute hepatitis C |  |
|  |  | B17.2 | Acute hepatitis E |  |
|  |  | B17.8 | Other specified acute viral hepatitis |  |
|  |  | B17.9 | Acute viral hepatitis, unspecified |  |
|  |  | B18.0 | Chronic viral hepatitis B with delta-agent |  |
|  |  | B18.2 | Chronic viral hepatitis C |  |
|  |  | B18.8 | Other chronic viral hepatitis |  |
|  |  | B18.9 | Chronic viral hepatitis, unspecified |  |
|  |  | B19.0 | Unspecified viral hepatitis with hepatic coma |  |
|  |  | B19.1 | Unspecified viral hepatitis B |  |
|  |  | B19.2 | Unspecified viral hepatitis C |  |
|  |  | K73.0 | Chronic persistent hepatitis, not elsewhere classified |  |
|  |  | K73.1 | Chronic lobular hepatitis, not elsewhere classified |  |
|  |  | K73.8 | Other chronic hepatitis, not elsewhere classified |  |
|  |  | K73.9 | Chronic hepatitis, unspecified |  |
| Other liver diseases | ICD-10 | K71.0 | Toxic liver disease with cholestasis |  |
|  |  | K71.1 | Toxic liver disease with hepatic necrosis |  |
|  |  | K71.2 | Toxic liver disease with acute hepatitis |  |
|  |  | K71.3 | Toxic liver disease with chronic persistent hepatitis |  |
|  |  | K71.4 | Toxic liver disease with chronic lobular hepatitis |  |
|  |  | K71.5 | Toxic liver disease with chronic active hepatitis |  |
|  |  | K71.6 | Toxic liver disease with hepatitis, not elsewhere classified |  |
|  |  | K71.7 | Toxic liver disease with fibrosis and cirrhosis of liver |  |
|  |  | K71.8 | Toxic liver disease with other disorders of liver |  |
|  |  | K71.9 | To K72 Hepatic failure, not elsewhere classified |  |
|  |  | K72.0 | Acute and subacute hepatic failure |  |
|  |  | K72.1 | Chronic hepatic failure |  |
|  |  | K72.9 | Hepatic failure, unspecified |  |
|  |  | K75.0 | Abscess of liver |  |
|  |  | K75.1 | Phlebitis of portal vein |  |
|  |  | K75.2 | Nonspecific reactive hepatitis |  |
|  |  | K75.3 | Granulomatous hepatitis, not elsewhere classified |  |
|  |  | K75.4 | Autoimmune hepatitis |  |
|  |  | K75.8 | Other specified inflammatory liver diseases |  |
|  |  | K75.9 | Inflammatory liver disease, unspecified |  |
|  |  | K76.1 | Chronic passive congestion of liver |  |
|  |  | K76.2 | Central hemorrhagic necrosis of liver |  |
|  |  | K76.3 | Infarction of liver |  |
|  |  | K76.4 | Peliosis hepatis |  |
|  |  | K76.5 | Hepatic veno -occlusive disease |  |
|  |  | K76.7 | Hepatorenal syndrome |  |
|  |  | K76.8 | Other specified diseases of liver |  |
|  |  | K76.9 | Liver disease, unspecified |  |
|  |  | K77 | Liver disorders in diseases classified elsewhere |  |
| Ascites | ICD-9 | 789.5 | Ascites |  |
|  | ICD-10 | R18 | Ascites |  |
| Variceal Bleeding | ICD-9 | 4560 | Oesophageal varices with bleeding |  |
|  | ICD-10 | I85.0 | Oesophageal varices with bleeding |  |
| SBP | ICD-9 | 567 | Peritonitis |  |
|  | ICD-10 | K65 | Peritonitis |  |
| Hepatic encephalopathy | ICD-9 | 5722 | Hepatic encephalopathy |  |
|  | ICD-10 | K76.8 | Hepatic encephalopathy |  |
| Hepatic failure | ICD-10 | K72.0 | Acute and subacute hepatic failure |  |
|  |  | K72.1 | Chronic hepatic failure |  |
|  |  | K72.9 | Hepatic failure, unspecified |  |
| Abbreviations: MASLD, metabolic dysfunction-associated steatotic liver disease; SBP, Spontaneous Bacterial Peritonitis. | | | | |

**Supplementary Table 3. Detailed missing status of covariates.**

| **Covariates** | **Missing n (%)** |
| --- | --- |
| Age | 0 |
| Sex | 0 |
| Townsend deprivation index | 0 |
| Education level | 0 |
| Smoking status | 0 |
| Alcohol consumption | 59 (0.17) |
| Body mass index | 0 |
| Physical activity | 1690 (4.86) |
| Healthy diet | 0 |
| Waist circumference | 0 |
| Hyperlipidemia | 0 |
| Hypertension | 0 |

**Supplementary Table 4. Genetic instruments for plasma proteome.**

Table can be obtained in OSF data respiratory: https://osf.io/t6byn/files/osfstorage.

**Supplementary Table 5. Summary results from Mendelian randomization (MR), Colocalization, and SMR for 3 proteome-wide MR identified proteins using *cis*-pQTLs.**

| **Diseases** | **Protein** | **Discovery** | | **Validation** | | SMR^A^ | | | **Colocalization** |
| --- | --- | --- | --- | --- | --- | --- | --- | --- | --- |
|  |  | **OR (95% CI)** | ***P*** | **OR (95% CI)** | ***P*** | **OR (95% CI)** | PSMR | PHEIDI | **PP.H4** |
| MASLD | NCAN | 0.53 (0.44, 0.64) | 1.56E-11 | 0.33 (0.21, 0.52) | 1.42E-06 | 0.54 (0.44, 0.65) | 1.49E-10 | 7.45E-09 | 0.0004 |
|  | GKRP | 0.43 (0.30, 0.62) | 5.98E-06 | 0.07 (0.03, 0.15) | 1.06E-10 | 0.42 (0.28, 0.63) | 3.15E-05 | 0.04 | 0.99 |
| ALD | ADH1B | 7.83 (2.32, 26.47) | 3.44E-06 | 7.99 (3.72, 17.17) | 1.00E-07 | 7.90 (3.04, 20.50) | 2.18E-05 | 0.67 | 0.44 |
| Liver cancer | NCAN | 0.20 (0.11, 0.34) | 9.98E-09 | 0.28 (0.14, 0.56) | 3.29E-04 | 0.28 (0.14, 0.57) | 4.04E-04 | 0.22 | 0.99 |
| Abbreviations: MASLD, metabolic dysfunction-associated steatotic liver disease; ALD, alcoholic liver disease; SMR, summary-data-based Mendelian randomization; HEIDI, heterogeneity in dependent instruments; OR, odds ratio; CI, confidence interval. NCAN, Neurocan core protein; GKRP, Glucokinase regulatory protein; ADH1B, Alcohol dehydrogenase 1B. ^A^The SMR analyses were performed based on discovery datasets. | | | | | | | | | |

| **Diseases** | **Protein** | **Methods** | **SNPs** | **Discovery** | | | **Validation** | | | **Colocalization** | **SMR** | | | |
| --- | --- | --- | --- | --- | --- | --- | --- | --- | --- | --- | --- | --- | --- | --- |
|  |  |  |  | **beta** | **se** | ***P* discovery** | **beta** | **se** | ***P* validation** | **PP.H4** | **beta** | **se** | ***P*_SMR_** | ***P*_HEIDI_** |
| **MASLD** | ADH1A | Wald ratio | 1 | 4.12 | 0.28 | 5.07E-48 | 8.59 | 0.58 | 2.60E-49 | 0.998 | 4.4 | 0.72 | 8.58E-10 | 0.61 |
|  | DCXR | Wald ratio | 1 | 4.81 | 0.33 | 5.07E-48 | 10.03 | 0.68 | 2.60E-49 | 0.998 | 4.5 | 0.75 | 2.08E-09 | 0.75 |
|  | AKR7A3 | Wald ratio | 1 | 3.21 | 0.22 | 5.07E-48 | 6.68 | 0.45 | 2.60E-49 | 0.999 | 3.27 | 0.42 | 1.32E-14 | 0.76 |
|  | A1BG | Wald ratio | 1 | 0.76 | 0.17 | 5.98E-06 | 2.45 | 0.38 | 1.06E-10 | 0.983 | 0.83 | 0.22 | 1.49E-04 | 0.67 |
|  | ACY1 | Wald ratio | 1 | 4.12 | 0.28 | 5.07E-48 | 8.59 | 0.58 | 2.60E-49 | 0.999 | 4.23 | 0.68 | 5.01E-10 | 0.59 |
|  | BMP1 | Wald ratio | 1 | 1.08 | 0.24 | 5.98E-06 | 3.5 | 0.54 | 1.06E-10 | 0.983 | 1.2 | 0.34 | 4.06E-04 | 0.35 |
|  | C4BPA | Wald ratio | 1 | 1.08 | 0.24 | 5.98E-06 | 3.5 | 0.54 | 1.06E-10 | 0.984 | 1.04 | 0.29 | 3.88E-04 | 0.71 |
|  | ERBB3 | IVW | 2 | 0.77 | 0.16 | 1.77E-06 | 2.43 | 0.39 | 3.72E-10 | 0.983 | 0.92 | 0.24 | 1.60E-04 | 0.91 |
|  | F9 | Wald ratio | 1 | 0.84 | 0.19 | 5.98E-06 | 2.73 | 0.42 | 1.06E-10 | 0.983 | 0.94 | 0.25 | 1.32E-04 | 0.79 |
|  | FBLN1 | Wald ratio | 1 | -1.26 | 0.28 | 5.98E-06 | -4.09 | 0.63 | 1.06E-10 | 0.983 | -1.06 | 0.38 | 0.01 | 0.62 |
|  | FTCD | Wald ratio | 1 | 4.81 | 0.33 | 5.07E-48 | 10.03 | 0.68 | 2.60E-49 | 0.998 | 4.83 | 0.84 | 1.03E-08 | 0.70 |
|  | IGDCC4 | Wald ratio | 1 | -1.49 | 0.33 | 7.52E-06 | -4.64 | 0.75 | 7.95E-10 | 0.984 | -0.5 | 0.51 | 0.33 | 0.16 |
|  | IGSF3 | Wald ratio | 1 | -1.51 | 0.33 | 5.98E-06 | -4.91 | 0.76 | 1.06E-10 | 0.983 | 0.84 | 0.39 | 0.03 | 0.86 |
|  | IL11RA | Wald ratio | 1 | -1.51 | 0.33 | 5.98E-06 | -4.91 | 0.76 | 1.06E-10 | 0.983 | 0.44 | 0.34 | 0.20 | 0.93 |
|  | MREG | Wald ratio | 1 | 1.51 | 0.33 | 5.98E-06 | 4.91 | 0.76 | 1.06E-10 | 0.983 | 1.45 | 0.43 | 7.64E-04 | 0.38 |
|  | NAB1 | Wald ratio | 1 | 1.26 | 0.28 | 5.98E-06 | 4.09 | 0.63 | 1.06E-10 | 0.976 | 1.31 | 0.38 | 5.00E-04 | 0.89 |
| **ALD** | ADH1A | Wald ratio | 1 | 5.24 | 0.37 | 4.66E-46 | 6.44 | 0.8 | 9.04E-16 | 0.998 | 5.7 | 0.97 | 4.12E-09 | 0.95 |
|  | DCXR | Wald ratio | 1 | 6.11 | 0.43 | 4.66E-46 | 7.52 | 0.94 | 9.04E-16 | 0.998 | 5.83 | 1.01 | 8.72E-09 | 0.94 |
|  | AKR7A3 | Wald ratio | 1 | 4.07 | 0.29 | 4.66E-46 | 5.01 | 0.62 | 9.04E-16 | 0.998 | 4.23 | 0.59 | 5.30E-13 | 0.47 |
|  | ACY1 | Wald ratio | 1 | 5.24 | 0.37 | 4.66E-46 | 6.44 | 0.8 | 9.04E-16 | 0.033 | 5.48 | 0.92 | 2.62E-09 | 0.33 |
|  | ADH1B | Wald ratio | 1 | 2.52 | 0.54 | 3.439E-06 | 2.08 | 0.39 | 1.00E-07 | 0.999 | 2.18 | 0.46 | 1.99E-06 | 0.57 |
| **Cirrhosis** | ADH1A | Wald ratio | 1 | 6.04 | 0.55 | 5.25E-28 | 6.93 | 0.4 | 3.46E-68 | 0.999 | 7.39 | 1.17 | 2.86E-10 | 0.38 |
|  | DCXR | Wald ratio | 1 | 7.05 | 0.64 | 5.25E-28 | 8.09 | 0.46 | 3.46E-68 | 0.999 | 7.57 | 1.23 | 7.65E-10 | 0.54 |
|  | HTR7 | Wald ratio | 1 | 1.24 | 0.24 | 3.48E-07 | 1.61 | 0.16 | 7.00E-24 | 0.394 | 1.61 | 0.22 | 4.09E-13 | 0.09 |
|  | TK2 | Wald ratio | 1 | 1.30 | 0.26 | 3.48E-07 | 1.69 | 0.17 | 7.00E-24 | 0.999 | 1.69 | 0.24 | 1.93E-12 | 0.18 |
| Abbreviations: MASLD, metabolic dysfunction-associated steatotic liver disease; ALD, alcoholic liver disease; SNPs, single nucleotide polymorphism; SMR, summary-data-based Mendelian randomization; HEIDI, heterogeneity in dependent instruments. ADH1A, Alcohol dehydrogenase 1A (class I); DCXR, Dicarbonyl and L-xylulose reductase; AKR7A3, Aldo-Keto Reductase Family 7 Member A3; A1BG, Alpha-1B-glycoprotein; ACY1, Aminoacylase-1; BMP1, Bone Morphogenetic Protein 1; C4BPA, Complement component 4 binding protein alpha; ERBB3, Erb-B2 Receptor Tyrosine Kinase 3; F9, Coagulation Factor IX; FBLN1, Fibulin 1; FTCD, Formimidoyltransferase cyclodeaminase; IGDCC4, Immunoglobulin Superfamily DCC Subclass Member 4; IGSF3, Immunoglobulin superfamily member 3; IL11RA, Interleukin 11 receptor subunit alpha; MREG, Melanoregulin; NAB1, NGFI-A binding protein 1; HTR7, 5-hydroxytryptamine receptor 7; TK2, Thymidine Kinase 2. | | | | | | | | | | | | | | |

**Supplementary Table 6. Summary results from Mendelian randomization (MR), Colocalization, and SMR for 19 proteome-wide MR identified proteins using all the pQTLs.**

**Supplementary Table 7. *Cis*-only Mendelian Randomization results after covariate adjustment.**

| **Exposure** | **Outcome** | **Adjustment** | **OR (95% CI)** | ***P*** |
| --- | --- | --- | --- | --- |
| GKRP | MASLD | None | 0.43 (0.30, 0.62) | 5.98E-06 |
| GKRP | MASLD | Body mass index | 0.98 (0.84, 1.15) | 0.84 |
| GKRP | MASLD | Waist hip rate | 1.02 (0.85, 1.22) | 0.86 |
| GKRP | MASLD | Cigarettes per day | 0.78 (0.62, 0.98) | 0.03 |
| GKRP | MASLD | Drinks per week | 0.74 (0.57, 0.95) | 0.02 |
| GKRP | MASLD | Diet pattern | 1.06 (0.85, 1.32) | 0.61 |
| GKRP | MASLD | Physical activity | 0.75 (0.57, 0.99) | 0.04 |
| GKRP | MASLD | Type 2 diabetes | 0.85 (0.61, 1.20) | 0.37 |
| GKRP | MASLD | All | 1.07 (0.93, 1.24) | 0.33 |
| NCAN | MASLD | None | 0.53 (0.44,0.64) | 1.56E-11 |
| NCAN | MASLD | Body mass index | 0.74 (0.60,0.92) | 6.01E-03 |
| NCAN | MASLD | Waist hip rate | 0.80 (0.60,1.06) | 0.12 |
| NCAN | MASLD | Cigarettes per day | 0.79 (0.42,1.49) | 0.46 |
| NCAN | MASLD | Drinks per week | 0.61 (0.35,1.04) | 0.07 |
| NCAN | MASLD | Diet pattern | 1.60 (1.13,2.26) | 8.43E-03 |
| NCAN | MASLD | Physical activity | 0.76 (0.36,1.62) | 0.48 |
| NCAN | MASLD | Type 2 diabetes | 0.74 (0.43,1.26) | 0.27 |
| NCAN | MASLD | All | 0.79 (0.66,0.95) | 0.01 |
| NCAN | Liver cancer | None | 0.20 (0.11,0.34) | 9.98E-09 |
| NCAN | Liver cancer | Body mass index | 0.83 (0.47, 1.47) | 0.53 |
| NCAN | Liver cancer | Waist hip rate | 0.98 (0.45,2.12) | 0.96 |
| NCAN | Liver cancer | Cigarettes per day | 1.95 (0.49,7.74) | 0.34 |
| NCAN | Liver cancer | Drinks per week | 0.68 (0.20,2.25) | 0.52 |
| NCAN | Liver cancer | Diet pattern | 0.68 (0.18,2.59) | 0.57 |
| NCAN | Liver cancer | Physical activity | 1.50 (0.20,11.45) | 0.70 |
| NCAN | Liver cancer | Type 2 diabetes | 1.70 (0.29,9.93) | 0.55 |
| NCAN | Liver cancer | All | 1.08 (0.65,1.80) | 0.77 |
| ADH1B | ALD | None | 7.83 (2.32, 26.47) | 3.44E-06 |
| ADH1B | ALD | Body mass index | 1.08 (0.83, 1.41) | 0.56 |
| ADH1B | ALD | Waist hip rate | 1.49 (1.09, 2.05) | 0.01 |
| ADH1B | ALD | Cigarettes per day | 3.04 (1.34, 6.91) | 8.07E-03 |
| ADH1B | ALD | Drinks per week | 2.82 (1.53, 5.21) | 9.06E-04 |
| ADH1B | ALD | Diet pattern | 1.18 (0.59, 2.35) | 0.64 |
| ADH1B | ALD | Physical activity | 3.00 (1.18, 7.63) | 0.02 |
| ADH1B | ALD | Type 2 diabetes | 3.26 (1.83, 5.83) | 6.43E-05 |
| ADH1B | ALD | All | 1.29 (1.06, 1.58) | 0.01 |
| Abbreviations: MASLD, metabolic dysfunction-associated steatotic liver disease; ALD, alcoholic liver disease; OR, odds ratio; CI, confidence interval. NCAN, Neurocan core protein; GKRP, Glucokinase regulatory protein; ADH1B, Alcohol dehydrogenase 1B. | | | | |

**Supplementary Table 8. *Cis*+*trans* Mendelian Randomization results after covariate adjustment.**

| **Exposure** | **Outcome** | **Adjustment** | **OR (95% CI)** | ***P*** |
| --- | --- | --- | --- | --- |
| A1BG | MASLD | Body mass index | 1.19 (0.99, 1.44) | 0.06 |
| ACY1 | MASLD | Body mass index | 1.95 (1.62, 2.35) | 2.51E-12 |
| ADH1A | MASLD | Body mass index | 2.22 (1.82, 2.71) | 3.61E-15 |
| AKR7A3 | MASLD | Body mass index | 2.12 (1.76, 2.56) | 4.25E-15 |
| BMP1 | MASLD | Body mass index | 1.20 (1.01, 1.43) | 0.04 |
| C4BPA | MASLD | Body mass index | 1.20 (1.00, 1.43) | 0.04 |
| DCXR | MASLD | Body mass index | 1.78 (1.47, 2.16) | 6.71E-09 |
| ERBB3 | MASLD | Body mass index | 1.12 (0.96, 1.31) | 0.14 |
| F9 | MASLD | Body mass index | 1.29 (1.10, 1.50) | 1.24E-03 |
| FBLN1 | MASLD | Body mass index | 0.82 (0.70, 0.97) | 0.02 |
| FTCD | MASLD | Body mass index | 1.99 (1.62, 2.44) | 4.33E-11 |
| IGDCC4 | MASLD | Body mass index | 0.72 (0.60, 0.87) | 5.28E-04 |
| IGSF3 | MASLD | Body mass index | 0.97 (0.83, 1.12) | 0.64 |
| IL11RA | MASLD | Body mass index | 0.91 (0.77, 1.07) | 0.25 |
| MREG | MASLD | Body mass index | 1.11 (0.91, 1.35) | 0.30 |
| NAB1 | MASLD | Body mass index | 1.42 (1.20, 1.69) | 6.04E-05 |
| A1BG | MASLD | Waist hip rate | 1.49 (1.20, 1.84) | 2.75E-04 |
| ACY1 | MASLD | Waist hip rate | 2.95 (2.36, 3.69) | 1.72E-21 |
| ADH1A | MASLD | Waist hip rate | 2.80 (2.21, 3.55) | 1.96E-17 |
| AKR7A3 | MASLD | Waist hip rate | 3.29 (2.59, 4.19) | 3.15E-22 |
| BMP1 | MASLD | Waist hip rate | 1.82 (1.48, 2.23) | 1.14E-08 |
| C4BPA | MASLD | Waist hip rate | 1.47 (1.17, 1.85) | 1.15E-03 |
| DCXR | MASLD | Waist hip rate | 2.88 (2.19, 3.79) | 4.04E-14 |
| ERBB3 | MASLD | Waist hip rate | 1.59 (1.29, 1.96) | 1.47E-05 |
| F9 | MASLD | Waist hip rate | 1.67 (1.37, 2.04) | 3.28E-07 |
| FBLN1 | MASLD | Waist hip rate | 0.72 (0.57, 0.89) | 2.95E-03 |
| FTCD | MASLD | Waist hip rate | 2.67 (2.04, 3.50) | 1.19E-12 |
| IGDCC4 | MASLD | Waist hip rate | 0.68 (0.54, 0.85) | 7.37E-04 |
| IGSF3 | MASLD | Waist hip rate | 0.90 (0.69, 1.16) | 0.42 |
| IL11RA | MASLD | Waist hip rate | 0.68 (0.53, 0.88) | 2.71E-03 |
| MREG | MASLD | Waist hip rate | 1.74 (1.38, 2.21) | 3.96E-06 |
| NAB1 | MASLD | Waist hip rate | 1.81 (1.43, 2.29) | 8.87E-07 |
| A1BG | MASLD | Cigarettes per day | 1.73 (1.21, 2.45) | 2.34E-03 |
| ACY1 | MASLD | Cigarettes per day | 6.60 (3.52, 12.38) | 3.97E-09 |
| ADH1A | MASLD | Cigarettes per day | 9.90 (5.24, 18.73) | 1.76E-12 |
| AKR7A3 | MASLD | Cigarettes per day | 7.60 (4.69, 12.33) | 2.03E-16 |
| BMP1 | MASLD | Cigarettes per day | 2.05 (1.36, 3.09) | 6.34E-04 |
| C4BPA | MASLD | Cigarettes per day | 2.02 (1.31, 3.12) | 1.48E-03 |
| DCXR | MASLD | Cigarettes per day | 7.48 (4.03, 13.88) | 1.90E-10 |
| ERBB3 | MASLD | Cigarettes per day | 1.95 (1.47, 2.60) | 4.50E-06 |
| F9 | MASLD | Cigarettes per day | 1.76 (1.27, 2.44) | 6.72E-04 |
| FBLN1 | MASLD | Cigarettes per day | 0.37 (0.25, 0.55) | 7.59E-07 |
| FTCD | MASLD | Cigarettes per day | 6.30 (3.19, 12.45) | 1.17E-07 |
| IGDCC4 | MASLD | Cigarettes per day | 0.36 (0.23, 0.57) | 9.86E-06 |
| IGSF3 | MASLD | Cigarettes per day | 0.56 (0.32, 0.96) | 0.04 |
| IL11RA | MASLD | Cigarettes per day | 0.44 (0.28, 0.71) | 6.08E-04 |
| MREG | MASLD | Cigarettes per day | 2.85 (1.73, 4.71) | 4.19E-05 |
| NAB1 | MASLD | Cigarettes per day | 2.75 (1.85, 4.08) | 5.95E-07 |
| A1BG | MASLD | Drinks per week | 1.59 (1.16, 2.16) | 3.65E-03 |
| ACY1 | MASLD | Drinks per week | 4.35 (2.61, 7.26) | 1.70E-08 |
| ADH1A | MASLD | Drinks per week | 4.79 (2.60, 8.83) | 4.87E-07 |
| AKR7A3 | MASLD | Drinks per week | 4.48 (2.84, 7.06) | 1.18E-10 |
| BMP1 | MASLD | Drinks per week | 1.37 (0.96, 1.95) | 0.08 |
| C4BPA | MASLD | Drinks per week | 1.57 (1.08, 2.30) | 0.02 |
| DCXR | MASLD | Drinks per week | 3.51 (1.88, 6.55) | 8.40E-05 |
| ERBB3 | MASLD | Drinks per week | 1.74 (1.26, 2.40) | 7.35E-04 |
| F9 | MASLD | Drinks per week | 1.66 (1.16, 2.35) | 4.98E-03 |
| FBLN1 | MASLD | Drinks per week | 0.66 (0.43, 1.00) | 0.05 |
| FTCD | MASLD | Drinks per week | 5.26 (3.09, 8.94) | 8.58E-10 |
| IGDCC4 | MASLD | Drinks per week | 0.37 (0.26, 0.53) | 8.66E-08 |
| IGSF3 | MASLD | Drinks per week | 0.53 (0.33, 0.83) | 0.01 |
| IL11RA | MASLD | Drinks per week | 0.41 (0.25, 0.65) | 1.68E-04 |
| MREG | MASLD | Drinks per week | 1.52 (0.97, 2.36) | 0.07 |
| NAB1 | MASLD | Drinks per week | 1.26 (0.79, 2.00) | 0.34 |
| A1BG | MASLD | Diet pattern | 1.96 (1.37, 2.81) | 2.54E-04 |
| ACY1 | MASLD | Diet pattern | 7.41 (4.25, 12.90) | 1.49E-12 |
| ADH1A | MASLD | Diet pattern | 7.72 (4.53, 13.15) | 5.30E-14 |
| AKR7A3 | MASLD | Diet pattern | 8.72 (5.52, 13.76) | 1.35E-20 |
| BMP1 | MASLD | Diet pattern | 2.34 (1.46, 3.75) | 4.36E-04 |
| C4BPA | MASLD | Diet pattern | 2.03 (1.30, 3.17) | 1.74E-03 |
| DCXR | MASLD | Diet pattern | 6.99 (3.77, 12.98) | 7.26E-10 |
| ERBB3 | MASLD | Diet pattern | 0.62 (0.46, 0.84) | 2.12E-03 |
| F9 | MASLD | Diet pattern | 0.91 (0.77, 1.08) | 0.30 |
| FBLN1 | MASLD | Diet pattern | 1.13 (0.97, 1.32) | 0.13 |
| FTCD | MASLD | Diet pattern | 0.76 (0.55, 1.05) | 0.09 |
| IGDCC4 | MASLD | Diet pattern | 1.58 (1.16, 2.15) | 3.66E-03 |
| IGSF3 | MASLD | Diet pattern | 1.04 (0.95, 1.14) | 0.38 |
| IL11RA | MASLD | Diet pattern | 1.08 (0.94, 1.23) | 0.27 |
| MREG | MASLD | Diet pattern | 0.91 (0.71, 1.17) | 0.47 |
| NAB1 | MASLD | Diet pattern | 0.83 (0.70, 0.98) | 0.03 |
| A1BG | MASLD | Physical activity | 2.22 (1.65, 3.00) | 1.80E-07 |
| ACY1 | MASLD | Physical activity | 21.75 (8.28, 57.16) | 4.14E-10 |
| ADH1A | MASLD | Physical activity | 22.73 (7.71, 67.02) | 1.50E-08 |
| AKR7A3 | MASLD | Physical activity | 14.62 (7.30, 29.29) | 3.80E-14 |
| BMP1 | MASLD | Physical activity | 2.52 (1.62, 3.92) | 4.41E-05 |
| C4BPA | MASLD | Physical activity | 2.99 (2.19, 4.07) | 4.61E-12 |
| DCXR | MASLD | Physical activity | 41.17 (15.96, 106.20) | 1.48E-14 |
| ERBB3 | MASLD | Physical activity | 2.14 (1.59, 2.87) | 4.25E-07 |
| F9 | MASLD | Physical activity | 2.40 (1.80, 3.19) | 2.66E-09 |
| FBLN1 | MASLD | Physical activity | 0.41 (0.23, 0.74) | 3.35E-03 |
| FTCD | MASLD | Physical activity | 41.15 (11.54, 146.83) | 1.01E-08 |
| IGDCC4 | MASLD | Physical activity | 0.32 (0.17, 0.57) | 1.44E-04 |
| IGSF3 | MASLD | Physical activity | 0.37 (0.17, 0.77) | 0.01 |
| IL11RA | MASLD | Physical activity | 0.35 (0.17, 0.72) | 4.31E-03 |
| MREG | MASLD | Physical activity | 0.47 (0.21, 1.05) | 0.06 |
| NAB1 | MASLD | Physical activity | 0.46 (0.23, 0.94) | 0.03 |
| A1BG | MASLD | Type 2 diabetes | 1.77 (1.11, 2.83) | 0.02 |
| ACY1 | MASLD | Type 2 diabetes | 2.28 (1.44, 3.58) | 3.88E-04 |
| ADH1A | MASLD | Type 2 diabetes | 2.47 (1.53, 4.00) | 2.29E-04 |
| AKR7A3 | MASLD | Type 2 diabetes | 2.24 (1.31, 3.83) | 3.10E-03 |
| BMP1 | MASLD | Type 2 diabetes | 2.58 (1.52, 4.38) | 4.24E-04 |
| C4BPA | MASLD | Type 2 diabetes | 1.73 (1.00, 3.01) | 0.05 |
| DCXR | MASLD | Type 2 diabetes | 3.18 (1.63, 6.21) | 6.84E-04 |
| ERBB3 | MASLD | Type 2 diabetes | 1.65 (1.00, 2.70) | 0.05 |
| F9 | MASLD | Type 2 diabetes | 2.24 (1.47, 3.44) | 2.01E-04 |
| FBLN1 | MASLD | Type 2 diabetes | 0.54 (0.30, 0.99) | 0.05 |
| FTCD | MASLD | Type 2 diabetes | 1.89 (1.06, 3.36) | 0.03 |
| IGDCC4 | MASLD | Type 2 diabetes | 0.41 (0.20, 0.88) | 0.02 |
| IGSF3 | MASLD | Type 2 diabetes | 0.99 (0.56, 1.78) | 0.98 |
| IL11RA | MASLD | Type 2 diabetes | 0.67 (0.36, 1.24) | 0.20 |
| MREG | MASLD | Type 2 diabetes | 3.21 (1.65, 6.25) | 5.97E-04 |
| NAB1 | MASLD | Type 2 diabetes | 3.11 (1.72, 5.62) | 1.79E-04 |
| A1BG | MASLD | All | 1.18 (1.03, 1.36) | 0.02 |
| ACY1 | MASLD | All | 1.63 (1.44, 1.85) | 1.75E-14 |
| ADH1A | MASLD | All | 1.65 (1.44, 1.89) | 3.64E-13 |
| AKR7A3 | MASLD | All | 1.61 (1.41, 1.84) | 3.07E-12 |
| BMP1 | MASLD | All | 1.34 (1.17, 1.53) | 2.04E-05 |
| C4BPA | MASLD | All | 1.09 (0.94, 1.25) | 0.25 |
| DCXR | MASLD | All | 1.45 (1.25, 1.68) | 6.08E-07 |
| ERBB3 | MASLD | All | 1.09 (0.96, 1.25) | 0.18 |
| F9 | MASLD | All | 1.26 (1.11, 1.44) | 3.25E-04 |
| FBLN1 | MASLD | All | 0.82 (0.71, 0.95) | 0.01 |
| FTCD | MASLD | All | 1.43 (1.25, 1.65) | 3.94E-07 |
| IGDCC4 | MASLD | All | 0.78 (0.68, 0.90) | 5.23E-04 |
| IGSF3 | MASLD | All | 1.00 (0.86, 1.16) | 1.00 |
| IL11RA | MASLD | All | 0.80 (0.69, 0.93) | 3.60E-03 |
| MREG | MASLD | All | 1.22 (1.06, 1.42) | 0.01 |
| NAB1 | MASLD | All | 1.47 (1.28, 1.70) | 1.15E-07 |
| ADH1A | Cirrhosis | Body mass index | 1.30 (0.99, 1.69) | 0.06 |
| DCXR | Cirrhosis | Body mass index | 1.13 (0.86, 1.49) | 0.38 |
| HTR7 | Cirrhosis | Body mass index | 1.02 (0.75, 1.39) | 0.89 |
| TK2 | Cirrhosis | Body mass index | 1.27 (0.93, 1.73) | 0.14 |
| ADH1A | Cirrhosis | Waist hip rate | 1.39 (1.00, 1.93) | 0.05 |
| DCXR | Cirrhosis | Waist hip rate | 1.16 (0.78, 1.71) | 0.47 |
| HTR7 | Cirrhosis | Waist hip rate | 1.00 (0.65, 1.52) | 0.99 |
| TK2 | Cirrhosis | Waist hip rate | 0.90 (0.59, 1.35) | 0.60 |
| ADH1A | Cirrhosis | Cigarettes per day | 0.56 (0.24, 1.32) | 0.19 |
| DCXR | Cirrhosis | Cigarettes per day | 0.51 (0.20, 1.28) | 0.15 |
| HTR7 | Cirrhosis | Cigarettes per day | 0.93 (0.18, 4.74) | 0.93 |
| TK2 | Cirrhosis | Cigarettes per day | 0.64 (0.13, 3.05) | 0.57 |
| ADH1A | Cirrhosis | Drinks per week | 0.84 (0.39, 1.78) | 0.64 |
| DCXR | Cirrhosis | Drinks per week | 0.79 (0.41, 1.51) | 0.47 |
| HTR7 | Cirrhosis | Drinks per week | 0.96 (0.52, 1.78) | 0.91 |
| TK2 | Cirrhosis | Drinks per week | 1.22 (0.61, 2.43) | 0.58 |
| ADH1A | Cirrhosis | Diet pattern | 1.80 (0.81, 3.99) | 0.15 |
| DCXR | Cirrhosis | Diet pattern | 1.56 (0.59, 4.16) | 0.37 |
| HTR7 | Cirrhosis | Diet pattern | 1.20 (0.34, 4.26) | 0.78 |
| TK2 | Cirrhosis | Diet pattern | 2.47 (0.83, 7.31) | 0.10 |
| ADH1A | Cirrhosis | Physical activity | 0.44 (0.24, 0.78) | 0.01 |
| DCXR | Cirrhosis | Physical activity | 0.28 (0.11, 0.73) | 0.01 |
| HTR7 | Cirrhosis | Physical activity | 0.27 (0.03, 2.03) | 0.20 |
| TK2 | Cirrhosis | Physical activity | 0.76 (0.17, 3.52) | 0.73 |
| ADH1A | Cirrhosis | Type 2 diabetes | 2.51 (1.27, 4.98) | 0.01 |
| DCXR | Cirrhosis | Type 2 diabetes | 5.10 (2.15, 12.11) | 2.18E-04 |
| HTR7 | Cirrhosis | Type 2 diabetes | 1.74 (0.46, 6.58) | 0.41 |
| TK2 | Cirrhosis | Type 2 diabetes | 3.08 (0.78, 12.15) | 0.11 |
| ADH1A | Cirrhosis | All | 1.36 (1.13, 1.65) | 1.28E-03 |
| DCXR | Cirrhosis | All | 1.20 (0.98, 1.47) | 0.07 |
| HTR7 | Cirrhosis | All | 0.97 (0.77, 1.22) | 0.78 |
| TK2 | Cirrhosis | All | 1.10 (0.88, 1.39) | 0.40 |
| ACY1 | ALD | Body mass index | 1.69 (1.31, 2.18) | 4.96E-05 |
| ADH1A | ALD | Body mass index | 1.60 (1.21, 2.10) | 8.20E-04 |
| ADH1B | ALD | Body mass index | 1.08 (0.83, 1.41) | 0.56 |
| AKR7A3 | ALD | Body mass index | 1.87 (1.45, 2.42) | 1.68E-06 |
| DCXR | ALD | Body mass index | 1.63 (1.26, 2.12) | 2.50E-04 |
| ACY1 | ALD | Waist hip rate | 2.06 (1.51, 2.80) | 4.31E-06 |
| ADH1A | ALD | Waist hip rate | 2.17 (1.58, 2.98) | 1.87E-06 |
| ADH1B | ALD | Waist hip rate | 1.49 (1.09, 2.05) | 0.01 |
| AKR7A3 | ALD | Waist hip rate | 2.68 (1.94, 3.71) | 2.77E-09 |
| DCXR | ALD | Waist hip rate | 2.47 (1.73, 3.54) | 7.86E-07 |
| ACY1 | ALD | Cigarettes per day | 12.67 (5.40, 29.69) | 5.16E-09 |
| ADH1A | ALD | Cigarettes per day | 15.00 (5.82, 38.70) | 2.12E-08 |
| ADH1B | ALD | Cigarettes per day | 3.04 (1.34, 6.91) | 0.01 |
| AKR7A3 | ALD | Cigarettes per day | 12.03 (5.92, 24.45) | 6.16E-12 |
| DCXR | ALD | Cigarettes per day | 9.74 (3.84, 24.70) | 1.62E-06 |
| ACY1 | ALD | Drinks per week | 5.34 (2.56, 11.14) | 7.82E-06 |
| ADH1A | ALD | Drinks per week | 10.74 (5.12, 22.49) | 3.16E-10 |
| ADH1B | ALD | Drinks per week | 2.82 (1.53, 5.21) | 9.06E-04 |
| AKR7A3 | ALD | Drinks per week | 7.80 (4.25, 14.30) | 3.14E-11 |
| DCXR | ALD | Drinks per week | 11.58 (5.38, 24.90) | 3.72E-10 |
| ACY1 | ALD | Diet pattern | 5.58 (2.52, 12.34) | 2.19E-05 |
| ADH1A | ALD | Diet pattern | 4.87 (2.19, 10.85) | 1.05E-04 |
| ADH1B | ALD | Diet pattern | 1.18 (0.59, 2.35) | 0.64 |
| AKR7A3 | ALD | Diet pattern | 6.66 (3.31, 13.43) | 1.13E-07 |
| DCXR | ALD | Diet pattern | 5.16 (2.20, 12.12) | 1.67E-04 |
| ACY1 | ALD | Physical activity | 41.66 (9.94, 174.51) | 3.34E-07 |
| ADH1A | ALD | Physical activity | 48.33 (10.50, 222.36) | 6.37E-07 |
| ADH1B | ALD | Physical activity | 3.00 (1.18, 7.63) | 0.02 |
| AKR7A3 | ALD | Physical activity | 27.55 (10.27, 73.94) | 4.57E-11 |
| DCXR | ALD | Physical activity | 97.66 (25.27, 377.47) | 3.10E-11 |
| ACY1 | ALD | Type 2 diabetes | 1.91 (1.26, 2.88) | 2.11E-03 |
| ADH1A | ALD | Type 2 diabetes | 1.97 (1.23, 3.13) | 4.40E-03 |
| ADH1B | ALD | Type 2 diabetes | 3.26 (1.83, 5.83) | 6.43E-05 |
| AKR7A3 | ALD | Type 2 diabetes | 2.35 (1.46, 3.78) | 4.20E-04 |
| DCXR | ALD | Type 2 diabetes | 3.20 (1.78, 5.75) | 1.06E-04 |
| ACY1 | ALD | All | 1.28 (1.08, 1.52) | 3.72E-03 |
| ADH1A | ALD | All | 1.31 (1.10, 1.58) | 3.26E-03 |
| ADH1B | ALD | All | 1.30 (1.06, 1.58) | 0.01 |
| AKR7A3 | ALD | All | 1.33 (1.11, 1.59) | 2.19E-03 |
| DCXR | ALD | All | 1.20 (0.99, 1.46) | 0.07 |
| Abbreviations: MASLD, metabolic dysfunction-associated steatotic liver disease; ALD, alcoholic liver disease; OR, odds ratio; CI, confidence interval. ADH1A, Alcohol dehydrogenase 1A (class I); ADH1B, Alcohol dehydrogenase 1B; DCXR, Dicarbonyl and L-xylulose reductase; AKR7A3, Aldo-Keto Reductase Family 7 Member A3; A1BG, Alpha-1B-glycoprotein; ACY1, Aminoacylase-1; BMP1, Bone Morphogenetic Protein 1; C4BPA, Complement component 4 binding protein alpha; ERBB3, Erb-B2 Receptor Tyrosine Kinase 3; F9, Coagulation Factor IX; FBLN1, Fibulin 1; FTCD, Formimidoyltransferase cyclodeaminase; IGDCC4, Immunoglobulin Superfamily DCC Subclass Member 4; IGSF3, Immunoglobulin superfamily member 3; IL11RA, Interleukin 11 receptor subunit alpha; MREG, Melanoregulin; NAB1, NGFI-A binding protein 1; HTR7, 5-hydroxytryptamine receptor 7; TK2, Thymidine Kinase 2. | | | | |

**Supplementary Table 9. Druggability of proteins potentially causally associated with liver diseases.**

| **Protein** | **Protein full name** | **Drug or component name** | **Drug groups** | **Indication** |
| --- | --- | --- | --- | --- |
| A1BG | Alpha-1B-glycoprotein | Copper | Approved, Investigational | For use in the supplementation of total parenteral nutrition and in contraception with intrauterine devices. |
|  |  | Zinc | Approved, Investigational | Zinc can be used for the treatment and prevention of zinc deficiency/its consequences, including stunted growth and acute diarrhea in children, and slowed wound healing. It is also utilized for boosting the immune system, treating the common cold and recurrent ear infections, as well as preventing lower respiratory tract infections. |
|  |  | Zinc Acetate | Approved, Investigational | Zinc can be used for the treatment and prevention of zinc deficiency/its consequences, including stunted growth and acute diarrhea in children, and slowed wound healing. It is also utilized for boosting the immune system, treating the common cold and recurrent ear infections, as well as preventing lower respiratory tract infections. |
| ACY1 | Aminoacylase-1 | Aspartic Acid | Approved, Nutraceutical | Aspartic acid is an amino acid commonly found as a component in total parenteral nutrition. |
|  |  | Acetylcysteine | Approved, Investigational | Acetylcysteine is indicated for mucolytic therapy and in the management of acetaminophen overdose. |
|  |  | Copper | Approved, Investigational | For use in the supplementation of total parenteral nutrition and in contraception with intrauterine devices. |
| ADH1A | Alcohol dehydrogenase 1A (class I) | Antizol | Approved, Vet approved | Antizol is indicated as an antidote for ethylene glycol (such as antifreeze) or methanol poisoning, or for use in suspected ethylene glycol or methanol ingestion, either alone or in combination with hemodialysis |
|  |  | NADH | Approved, Nutraceutical | Some evidence suggests that NADH might be useful in treating Parkinson's disease, chronic fatigue syndrome, Alzheimer's disease, and cardiovascular disease. |
|  |  | 4-Iodopyrazole | Experimental | Not Available. |
|  |  | N-Cyclopentyl-N-Cyclobutylformamide | Experimental | Not Available. |
|  |  | Ethanol | Approved | For therapeutic neurolysis of nerves or ganglia for the relief of intractable chronic pain in such conditions as inoperable cancer and trigeminal neuralgia (tic douloureux), in patients for whom neurosurgical procedures are contraindicated. |
|  |  | Acetaldehyde | Preclinical | Not Available. |
|  |  | Abacavir | Approved, Investigational | Abacavir is indicated in combination with other anti-retroviral agents for the treatment of HIV-1 infection. It is available in a combination product. alongside dolutegravir and lamivudine for the treatment of adult and pediatric patients with HIV-1 who weigh more than 10 kg. |
| AKR7A3 | Aldo-Keto Reductase Family 7 Member A3 | - | - | - |
| BMP1 | Bone Morphogenetic Protein 1 | - | - | - |
| C4BPA | Complement component 4 binding protein alpha | Copper | Approved, Investigational | For use in the supplementation of total parenteral nutrition and in contraception with intrauterine devices. |
|  |  | Zinc | Approved, Investigational | Zinc can be used for the treatment and prevention of zinc deficiency/its consequences, including stunted growth and acute diarrhea in children, and slowed wound healing. It is also utilized for boosting the immune system, treating the common cold and recurrent ear infections, as well as preventing lower respiratory tract infections. |
|  |  | Zinc acetate | Approved, Investigational | Zinc can be used for the treatment and prevention of zinc deficiency/its consequences, including stunted growth and acute diarrhea in children, and slowed wound healing. It is also utilized for boosting the immune system, treating the common cold and recurrent ear infections, as well as preventing lower respiratory tract infections. |
|  |  | Zinc chloride | Approved, Investigational | Zinc chloride injections are indicated for use total parenteral nutrition to maintain zinc serum levels and prevent deficiency syndromes. |
|  |  | Zinc sulfate, unspecified form | Approved, Experimental | Zinc sulfate is a common zinc supplement in parenteral nutrition. |
| DCXR | Dicarbonyl and L-xylulose reductase | - | - | - |
| ERBB3 | Erb-B2 Receptor Tyrosine Kinase 3 | MM-121 | Investigational | Non-small cell lung carcinoma, cancer, neoplasm, peritoneum cancer, fallopian tube cancer, breast cancer, ovarian cancer. |
|  |  | Lumretuzumab | Investigational | Neoplasm, non-small cell lung carcinoma, breast cancer. |
|  |  | Seribantumab | Investigational | Pancreatic carcinoma, breast cancer. |
|  |  | Istiratumab | Investigational | Pancreatic carcinoma, hepatocellular carcinoma. |
|  |  | CDX-3379 | Investigational | Melanoma, head, and neck squamous cell carcinoma. |
|  |  | Tucatinib | Approved, Investigational | Tucatinib is indicated with trastuzumab and capecitabine for the treatment of adults diagnosed with advanced unresectable or metastatic HER2-positive breast cancer. This includes patients with brain metastases and those who have received one or more prior anti-HER2-based regimens in the metastatic setting. It is also indicated in combination with trastuzumab for the treatment of adult patients with RAS wild-type HER2-positive unresectable or metastatic colorectal cancer that has progressed following treatment with fluoropyrimidine-, oxaliplatin-, and irinotecan-based chemotherapy. |
|  |  | AV-203 | Investigational | Neoplasm. |
|  |  | AMG-888 | Investigational | Non-small cell lung carcinoma, breast cancer, neoplasm. |
|  |  | Patritumab | Investigational | Not Available. |
|  |  | Duligotuzumab | Investigational | Not Available. |
|  |  | Sapitinib | Investigational | Not Available. |
|  |  | MM-111 | Investigational | Breast cancer. |
|  |  | Pertuzumab | Approved | Pertuzumab is indicated for intravenous administration in combination with trastuzumab and docetaxel for the treatment of patients with HER2-positive metastatic breast cancer who have not received prior anti-HER2 therapy or chemotherapy for metastatic disease.6 It is also indicated in combination with trastuzumab and other chemotherapies for the neoadjuvant treatment of HER2-positive locally advanced, inflammatory, or early-stage breast cancer as part of a complete treatment regimen6 and as adjuvant treatment in patients with HER2-positive early-stage breast cancer at high risk of recurrence. Pertuzumab is also indicated for subcutaneous injection - in combination with trastuzumab and hyaluronidase - in the treatment of HER2-positive breast cancers in adults. |
|  |  | Elgemtumab | Investigational | Not Available. |
|  |  | Methylcurcumin | Preclinical | Not Available. |
| F9 | Coagulation Factor IX | Emicizumab | Approved, Investigational | The main function of Emicizumab is the prevention of bleeding episodes. Thus, Emicizumab is approved for the routine prophylaxis to prevent or reduce the frequency of bleeding episodes of adult and pediatric patients with hemophilia A with or without Factor VIII inhibitors. Hemophilia A is a deficiency of coagulation Factor VIII which causes a serious bleeding disorder. The standard treatment is done with the administration of recombinant or serum-deriver Factor VIII which induces the formation of anti-factor VIII alloantibodies (Factor VIII inhibitors) and renders the standard treatment ineffective. |
|  |  | Turoctocog alfa | Investigational | Ataxia telangiectasia, Hemophilia B, Tay-Sachs disease; Sandhoff disease; Niemann-Pick disease type C. |
|  |  | Nonacog Alfa | Investigational | Hemophilia B. |
|  |  | Nonacog beta pegol | Investigational | Hemophilia B. |
|  |  | Eftrenonacog alfa | Investigational | Hemophilia B, hemophilia. |
|  |  | Fidanacogene elaparvovec | Investigational | Hemophilia B. |
|  |  | Antihemophilic Factor, Human Recombinant | Approved, Investigational | The human recombinant antihemophilic factor is indicated for use in adults and children with hemophilia A for the control and prevention of bleeding episodes, perioperative management, and routine prophylaxis to prevent or reduce the frequency of bleeding episodes |
|  |  | TTP889 | Investigational | Investigated for use/treatment in blood (blood forming organ disorders, unspecified), cardiac surgery, and venous thromboembolism. |
|  |  | Coagulation Factor VII Human | Approved, Investigational | May be administered in cases of uncontrolled bleeding. Factor VII alone can be used in the treatment of congenital hemophilia A or B, acquired hemophilia, congenital factor VII deficiency, and Glanzmann's thrombasthenia. Off label use in the treatment of refractory bleeding after cardiac surgery and warfarin related intracerebral hemorrhage. Brands for human factor VII are currently only in combination with other vitamin K coagulation factors and can be used to reverse vitamin K antagonist activity in patients with acute major bleeds or for urgent surgery/invasive procedures. |
|  |  | Antihemophilic Factor Human | Approved | The human antihemophilic factor is indicated for the cases of hemophilia A, also known as classical hemophilia for the prevention and control of hemorrhagic episodes. If surgery is needed in patients with hemophilia A there is a need of correction of the clotting abnormality. In these cases, the human antihemophilic factor may be administered followed by intermittent maintenance doses. The hemophilia A is characterized by the deficiency of the coagulation factor VIII that results in prolonged blood flow after injury or surgery as well as recurrent bleeding. |
|  |  | Turoctocog Alfa | Approved, Investigational | Turoctocog alfa is indicated for the treatment and prophylaxis of bleedings in patients presenting hemophilia A. The treatment with turoctocog alfa is related with its use to control bleeding episodes or as a perioperative management. Hemophilia A is a hereditary hemorrhagic disorder generated by the congenital deficit of the coagulation factor VIII. This disease is manifested as excessive spontaneous or trauma-driven bleeding. The coagulation factor VIII is a robust initiator of thrombin which is later required for the generation of fibrin to form a platelet plug and its gene is expressed in the X chromosome. |
|  |  | Kappadione | Approved | Anticoagulant-induced prothrombin deficiency caused by coumadin or indanedione derivatives, prophylaxis and therapy of hemorrhagic disease of the newborn, hypoprothrombinemia due to antibacterial therapy, hypoprothrombinemia secondary to factors limiting absorption or synthesis of vitamin K (for example, obstructive jaundice, biliary fistula, sprue, ulcerative colitis, celiac disease, intestinal resection, cystic fibrosis of the pancreas, and regional enteritis, other drug-induced hypoprothrombinemia where it is definitely shown that the result is due to interference with vitamin K metabolism). |
|  |  | Lonoctocog Alfa | Approved, Investigational | Not Available. |
|  |  | Moroctocog Alfa | Approved | Moroctocog alfa is indicated for use in adults and children with hemophilia A (congenital factor VIII deficiency) for on-demand treatment and control of bleeding episodes, perioperative management, and routine prophylaxis to reduce the frequency of bleeding episodes. |
|  |  | Turoctocog Alfa Pegol | Approved | Turoctocog alfa pegol is indicated for use in adults and children with hemophilia A for on-demand treatment and control of bleeding episodes, perioperative management of bleeding, and routine prophylaxis to reduce the frequency of bleeding episodes. It is not indicated for the treatment of von Willebrand disease. |
|  |  | Efanesoctocog Alfa | Approved, Investigational | Efanesoctocog alfa is indicated for use in adults and children with hemophilia A (congenital factor VIII deficiency) for routine prophylaxis to reduce the frequency of bleeding episodes, on-demand treatment and control of bleeding episodes, and perioperative management of bleeding. |
|  |  | Pegnivacogin | Investigational | Investigated for use/treatment in thrombosis, coronary artery disease, and vascular diseases. |
|  |  | Levothyroxine | Approved | Levothyroxine is indicated as replacement therapy in primary (thyroidal), secondary (pituitary) and tertiary (hypothalamic) congenital or acquired hypothyroidism. It is also indicated as an adjunct to surgery and radioiodine therapy in the management of thyrotropin-dependent well-differentiated thyroid cancer. |
|  |  | Coumarin | Experimental | Not Available. |
|  |  | Menadione | Approved, Nutraceutical | The primary known function of vitamin K is to assist in the normal clotting of blood, but it may also play a role in normal bone calcification. |
|  |  | Hydrocortisone | Approved,  Vet approved | Otic solutions are indicated for infections of the external auditory canal caused by susceptible organisms and with inflammation. Hydrocortisone tablets are indicated for certain endocrine, rheumatic, collagen, allergic, ophthalmic, respiratory, hematologic, neoplastic, edematous, gastrointestinal, and other conditions. A hydrocortisone enema is indicated for ulcerative colitis, a topical ointment with antibiotics is indicated for corticosteroid responsive dermatoses with infections, and a topical cream with acyclovir is indicated to treat cold sores. Oral granules of hydrocortisone are used as a replacement therapy for Adrenocortical Insufficiency (AI) in children under years of age. |
|  |  |  |  |  |
|  |  | Dexamethasone | Approved, Investigational, Vet approved | Dexamethasone and ciprofloxacin otic suspension is indicated for bacterial infections with inflammation in acute otitis media and acute otitis externa. Intramuscular and intravenous injections are indicated for a few endocrine, rheumatic, collagen, dermatologic, allergic, ophthalmic, gastrointestinal, respiratory, hematologic, neoplastic, edematous, and other conditions. Oral tablets are indicated for the treatment of multiple myeloma. An intravitreal implant is indicated for some forms of macular edema and non-infectious posterior uveitis affecting the posterior of the eye. Various ophthalmic formulations are indicated for inflammatory conditions of the eye. |
| FBLN1 | Fibulin 1 | - | - | - |
| FTCD | Formimidoyltransferase cyclodeaminase | Glutamic Acid | Approved, Nutraceutical | Considered to be nature's "Brain food" by improving mental capacities; helps speed the healing of ulcers; gives a "lift" from fatigue; helps control alcoholism, schizophrenia, and the craving for sugar. |
|  |  | (6R)-Folinic Acid | Experimental | Not Available |
|  |  | Tetrahydrofolic Acid | Nutraceutical | For nutritional supplementation, also for treating dietary shortage or imbalance. |
| IGDCC4 | Immunoglobulin Superfamily DCC Subclass Member 4 | - | - | - |
| IGSF3 | Immunoglobulin superfamily member 3 | - | - | - |
| IL11RA | Interleukin 11 receptor subunit alpha | Oprelvekin | Approved, Investigational | Indicated for the prevention of severe thrombocytopenia and the reduction of the need for platelet transfusions following myelosuppressive chemotherapy in adult patients with nonmyeloid malignancies who are at high risk of severe thrombocytopenia. |
| MREG | Melanoregulin | - | - | - |
| NAB1 | NGFI-A binding protein 1 | - | - | - |
| HTR7 | 5-hydroxytryptamine receptor 7 | Fukinolic Acid | Preclinical | Not Available. |
|  |  | Cimipronidine | Preclinical | Not Available. |
|  |  | JNJ-18038683 | Investigational | Major depressive disorder；bipolar disorder |
|  |  | LY2590443 | Investigational | Migraine Disorders. |
|  |  | Loxapine | Approved | For the management of the manifestations of psychotic disorders such as schizophrenia |
|  |  | Imipramine | Approved | For the relief of symptoms of depression and as temporary adjunctive therapy in reducing enuresis in children aged 6 years and older. May also be used off-label to manage panic disorders with or without agoraphobia, as a second line agent for ADHD in children and adolescents, to manage bulimia nervosa, for short-term management of acute depressive episodes in bipolar disorder and schizophrenia, for the treatment of acute stress disorder and posttraumatic stress disorder, and for symptomatic treatment of postherpetic neuralgia and painful diabetic neuropathy. |
|  |  | Amoxapine | Approved | For the relief of symptoms of depression in patients with neurotic or reactive depressive disorders as well as endogenous and psychotic depressions. May also be used to treat depression accompanied by anxiety or agitation. |
|  |  | Dopamine | Approved | For the correction of hemodynamic imbalances present in the shock syndrome due to myocardial infarction, trauma, endotoxic septicemia, open-heart surgery, renal failure, and chronic cardiac decompensation as in congestive failure. |
|  |  | Amitriptyline | Approved | Major depressive disorder in adults; Management of neuropathic pain in adults; Prophylactic treatment of chronic tension-type headache (CTTH) in adults; Prophylactic treatment of migraine in adults; Treatment of nocturnal enuresis in children aged 6 years and above when organic pathology, including spina bifida and related disorders, have been excluded and no response has been achieved to all other non-drug and drug treatments, including antispasmodics and vasopressin-related products. This product should only be prescribed by a healthcare professional with expertise in the management of persistent enuresis. Off-label uses: irritable bowel syndrome, sleep disorders, diabetic neuropathy, agitation, fibromyalgia, and insomnia. |
|  |  | Maprotiline | Approved, Investigational | For treatment of depression, including the depressed phase of bipolar depression, psychotic depression, and involutional melancholia, and may also be helpful in treating certain patients suffering severe depressive neurosis. |
|  |  | Mianserin | Approved, Investigational | For the treatment of depression. |
|  |  | Vortioxetine | Approved, Investigational | Vortioxetine is indicated for the treatment of major depressive disorder (MDD). |
|  |  | Lisuride | Approved, Investigational | For the management of Parkinson's Disease |
|  |  | Paliperidone | Approved | As an oral extended-release tablet and a once-monthly extended-release suspension for intramuscular injection, paliperidone is indicated for the treatment of adults and adolescents with schizophrenia and in the treatment of schizoaffective disorder in combination with antidepressants or mood stabilizers. Paliperidone is also available in both an every-three-month and twice-yearly extended-release suspension for intramuscular injection for the treatment of schizophrenia. |
|  |  | Cyproheptadine | Approved | In the US, prescription cyproheptadine is indicated for the treatment of various allergic symptomatologies, including dermatographia, rhinitis, conjunctivitis, and urticaria - as well as adjunctive therapy in the management of anaphylaxis following treatment with epinephrine. In Canada, cyproheptadine is available over-the-counter and is indicated for the treatment of pruritus and for appetite stimulation. In Australia, cyproheptadine is additionally indicated for the treatment vascular headaches. Cyproheptadine is also used off-label for the treatment of serotonin syndrome. |
|  |  | Sb-269970 | investigational | Not Available. |
|  |  | Zotepine | Approved, Investigational, withdrawn | Zotepine, like other atypical antipsychotics, is considered as the first-line treatment in newly diagnosed schizophrenia. It is usually thought to be an option of choice for managing acute schizophrenic episodes when discussion with the patient is not possible. Zotepine, as an atypical antipsychotic, is used in patients who are suffering unacceptable side effects from conventional antipsychotics or in relapse patients that were inadequately controlled. It is important to consider that the indications stated above are related to atypical antipsychotics, that zotepine is not currently FDA, Canada or EMA approved and that studies have not shown any additional benefit when compared with other approved atypical antipsychotics. Schizophrenia is a chronic and severe mental disorder that affects how a person thinks, feels, and behaves. It is usually marked for a loose reality perspective delineated by hallucinations, delusions and thought and movement disorders. |
|  |  | Lofexidine | Approved, Investigational | Lofexidine is indicated for mitigation of symptoms associated with acute withdrawal from opioids and for facilitation of the completion of opioid discontinuation treatment. It is the first non-opioid medication for the symptomatic management of opioid discontinuation. Opioid withdrawal syndrome is a debilitating manifestation of opioid dependence. This condition is extremely unpleasant lasting several days with some of the main features being abdominal pain, nausea, diarrhea, mydriasis, lacrimation, and piloerection. These symptoms are often observed after abrupt reductions in the opioid dose and can be resolved by re-administration of the opioid. |
|  |  | Aripiprazole Lauroxil | Approved, Investigational | Aripiprazole lauroxil is indicated for the treatment of schizophrenia and related psychotic disorders. |
|  |  | Haloperidol | Approved | Haloperidol is indicated for several conditions including for the treatment of schizophrenia, for the manifestations of psychotic disorders, for the control of tics and vocal utterances of Tourette’s Disorder in children and adults, for treatment of severe behavior problems in children of combative, explosive hyperexcitability (which cannot be accounted for by immediate provocation). Haloperidol is also indicated in the short-term treatment of hyperactive children who show excessive motor activity with accompanying conduct disorders consisting of some or all the following symptoms: impulsivity, difficulty sustaining attention, aggressivity, mood lability, and poor frustration tolerance. Haloperidol should be reserved for these two groups of children only after failure to respond to psychotherapy or medications other than antipsychotics. |
|  |  | Cyclobenzaprine | Approved | Cyclobenzaprine is indicated as a short-term (2-3 weeks) adjunct therapy, along with rest and physical therapy, for relief of muscle spasm associated with acute, painful musculoskeletal conditions. It has not been found effective in the treatment of spasticity originating from cerebral or spinal cord disease, or spasticity in children with cerebral palsy. Cyclobenzaprine is also occasionally used off-label for reducing pain and sleep disturbances in patients with fibromyalgia. |
|  |  | Risperidone | Approved, Investigational | Risperidone is indicated for the treatment of schizophrenia and irritability associated with autistic disorder. It is also indicated as monotherapy, or adjunctly with lithium or valproic acid, for the treatment of acute mania or mixed episodes associated with bipolar I disorder. Risperidone is additionally indicated in Canada for the short-term symptomatic management of aggression or psychotic symptoms in patients with severe dementia of the Alzheimer type unresponsive to nonpharmacological approaches. Risperidone is also used off-label for several conditions including as an adjunct to antidepressants in treatment-resistant depression. |
|  |  | Zolmitriptan | Approved, Investigational | Zolmitriptan is indicated for the acute treatment of migraine with or without auras in patients aged 18 and over. |
|  |  | Chlorpromazine | Approved, Investigational, Vet approved | For the treatment of schizophrenia; to control nausea and vomiting; for relief of restlessness and apprehension before surgery; for acute intermittent porphyria; as an adjunct in the treatment of tetanus; to control the manifestations of the manic type of manic-depressive illness; for relief of intractable hiccups; for the treatment of severe behavioral problems in children (1 to 12 years of age) marked by combativeness and/or explosive hyperexcitable behavior (out of proportion to immediate provocations), and in the short-term treatment of hyperactive children who show excessive motor activity with accompanying conduct disorders consisting of some or all of the following symptoms: impulsivity, difficulty sustaining attention, aggressivity, mood lability, and poor frustration tolerance. |
|  |  | Iloperidone | Approved | Treatment of acute schizophrenia. |
|  |  | Lurasidone | Approved, Investigational | Lurasidone is indicated for the treatment of schizophrenia in patients ≥13 years old. It is also indicated as a monotherapy for the treatment of bipolar depression in patients ≥10 years old, or in combination with lithium or valproate for the treatment of bipolar depression in adults. |
|  |  | Amisulpride | Approved, Investigational | Intravenous amisulpride is indicated in adults for the prevention of postoperative nausea and vomiting, either alone or in combination with an antiemetic of a different class. It is also indicated for the treatment of postoperative nausea and vomiting in patients who have received anti-emetic prophylaxis with an agent of a different class or have not received prophylaxis. Oral amisulpride is indicated for the treatment of acute and chronic schizophrenic disorders, characterized by positive symptoms with delusions, hallucinations, thought disorders, hostility, and suspicious behavior; or primarily negative symptoms (deficit syndrome) with blunted affect, emotional and social withdrawal. Amisulpride also controls secondary negative symptoms in productive conditions as well as affective disorders such as depressive mood or retardation. |
|  |  | Cabergoline | Approved | For the treatment of hyperprolactinemic disorders, either idiopathic or due to prolactinoma (prolactin-secreting adenomas). May also be used to manage symptoms of Parkinsonian Syndrome as monotherapy during initial symptomatic management or as an adjunct to levodopa therapy during advanced stages of disease. |
|  |  | Quetiapine | Approved | Quetiapine is used in the symptomatic treatment of schizophrenia. In addition, it may be used for the management of acute manic or mixed episodes in patients with bipolar I disorder, as a monotherapy or combined with other drugs. It may be used to manage depressive episodes in bipolar disorder. In addition to the above indications, quetiapine is used in combination with antidepressant drugs for the treatment of major depression. Some off-label uses for this drug include the management of post-traumatic stress disorder (PTSD), generalized anxiety disorder, and psychosis associated with Parkinson's disease. |
|  |  | Aripiprazole | Approved, Investigational | Aripiprazole is indicated for manic and mixed episodes associated with bipolar I disorder, irritability associated with autism spectrum disorder, treatment of schizophrenia, treatment of Tourette's disorder, and as an adjunctive treatment of major depressive disorder Label. An injectable formulation of aripiprazole is indicated for agitation associated with schizophrenia or bipolar mania. |
|  |  | Clozapine | Approved | Clozapine is indicated for the treatment of severely ill patients with schizophrenia who fail to respond adequately to standard antipsychotic treatment. Because of the risks of severe neutropenia and of seizure associated with its use, Clozapine should be used only in patients who have failed to respond adequately to standard antipsychotic treatment. Clozapine is also indicated for reducing the risk of recurrent suicidal behavior in patients with schizophrenia or schizoaffective disorder who are judged to be at chronic risk for re-experiencing suicidal behavior, based on history and recent clinical state. Suicidal behavior refers to actions by a patient that put him/herself at risk for death. |
|  |  | Epinastine | Approved, Investigational | For the prevention of itching associated with allergic conjunctivitis. |
|  |  | Bromocriptine | Approved, Investigational, Withdrawn | For the treatment of galactorrhea due to hyperprolactinemia, prolactin-dependent menstrual disorders and infertility, prolactin-secreting adenomas, prolactin-dependent male hypogonadism, as adjunct therapy to surgery or radiotherapy for acromegaly or as monotherapy is special cases, as monotherapy in early Parksinsonian Syndrome or as an adjunct with levodopa in advanced cases with motor complications. Bromocriptine has also been used off-label to treat restless legs syndrome and neuroleptic malignant syndrome. |
|  |  | Asenapine | Approved | Used for treatment in psychosis, schizophrenia and schizoaffective disorders, manic disorders, and bipolar disorders as monotherapy or in combination. |
|  |  | Vortioxetine | Approved, Investigational | Vortioxetine is indicated for the treatment of major depressive disorder (MDD). |
|  |  | Methysergide | Approved | For the treatment of vascular headache. |
|  |  | Ziprasidone | Approved | In its oral form, ziprasidone is approved for the treatment of schizophrenia, as monotherapy for acute treatment of manic or mixed episodes related to bipolar, I disorder, and as adjunctive therapy to lithium or valproate for maintenance treatment of bipolar I disorder.13 The injectable formulation is approved only for treatment of acute agitation in schizophrenia. |
| TK2 | Thymidine Kinase 2 | Zalcitabine | Approved, Investigational | For the treatment of Human immunovirus (HIV) infections in conjunction with other antivirals. |
|  |  | Thymidine 5'-Triphosphate | Experimental | Not Available. |
|  |  | 2'-Deoxycytidine | Experimental, Investigational | Not Available. |
|  |  | Thymidine | Experimental, Investigational | Not Available. |
|  |  | Brivudine | Approved, Investigational | Not Available. |
|  |  | Gemcitabine | Approved | Gemcitabine is a chemotherapeutic agent used as monotherapy or in combination with other anticancer agents. In combination with carboplatin, it is indicated for the treatment of advanced ovarian cancer that has relapsed at least 6 months after completion of platinum-based therapy. Gemcitabine in combination with paclitaxel is indicated for the first-line treatment of patients with metastatic breast cancer after failure of prior anthracycline-containing adjuvant chemotherapy, unless anthracyclines were clinically contraindicated. In combination with cisplatin, gemcitabine is indicated for the first-line treatment of patients with inoperable, locally advanced (Stage IIIA or IIIB) or metastatic (Stage IV) non-small cell lung cancer (NSCLC). Dual therapy with cisplatin is also used to treat patients with Stage IV (locally advanced or metastatic) transitional cell carcinoma (TCC) of the bladder. Gemcitabine is indicated as first-line treatment for patients with locally advanced (nonresectable Stage II or Stage III) or metastatic (Stage IV) adenocarcinoma of the pancreas. Gemcitabine is indicated for patients previously treated with fluorouracil. |
| NCAN | Neurocan core protein | Beta-D-Glucose | Experimental | Not Available |
|  |  | Thiodigalactoside | Experimental | Not Available. |
|  |  | Hyaluronic acid | Approved, Vet_approved | The intra-articular preparations of hyaluronic acid are indicated for knee pain associated with osteoarthritis. Hyaluronic acid is used in cosmetic applications to prevent and reduce the appearance of wrinkles on the face, and as a dermal filler to correct facial imperfections or other imperfections on other parts of the body. It is frequently an ingredient in topical applications for wound healing and symptomatic treatment of skin irritation from various causes. Hyaluronic acid may also be indicated in ophthalmological preparations or oral capsules to treat discomfort caused by dry eyes or conjunctivitis and for its protective qualities during and before eye surgery.Finally, hyaluronic acid can be used off-label to coat the bladder for relief of interstitial cystitis symptoms. |
| GKRP | Glucokinase regulatory protein | - | - | - |
| ADH1B | Alcohol dehydrogenase 1B | NADH | Approved, Nutraceutical | Some evidence suggests that NADH might be useful in treating Parkinson's disease, chronic fatigue syndrome, Alzheimer's disease and cardiovascular disease. |
|  |  | Fomepizole | Approved, Vet_approved | Antizol is indicated as an antidote for ethylene glycol (such as antifreeze) or methanol poisoning, or for use in suspected ethylene glycol or methanol ingestion, either alone or in combination with hemodialysis |
|  |  | N-Benzylformamide | Experimental | Not Available. |
|  |  | 4-Iodopyrazole | Experimental | Not Available. |
|  |  | Cyclohexanol | Experimental | Not Available. |
|  |  | N-Heptylformamide | Experimental | Not Available. |
|  |  | Nicotinamide adenine dinucleotide phosphate | Experimental | Not Available. |
|  |  | Glycerin | Approved, Investigational | It is used as a solvent, emollient, pharmaceutical agent, and sweetening agent. |
|  |  | NADH | Approved, Investigational | Some evidence suggests that NADH might be useful in treating Parkinson's disease, chronic fatigue syndrome, Alzheimer's disease and cardiovascular disease. |
|  |  | Ethanol | Approved | For therapeutic neurolysis of nerves or ganglia for the relief of intractable chronic pain in such conditions as inoperable cancer and trigeminal neuralgia (tic douloureux), in patients for whom neurosurgical procedures are contraindicated. |

**Supplementary Table 10. Functions of identified proteins.**

| **Protein** | **Function** |
| --- | --- |
| ADH1A | Alcohol dehydrogenase; Oxidizes primary as well as secondary alcohols. Ethanol is a very poor substrate. |
| NCAN | May modulate neuronal adhesion and neurite growth during development by binding to neural cell adhesion molecules (NG-CAM and N-CAM). Chondroitin sulfate proteoglycan; binds to hyaluronic acid. |
| GKRP | Regulates glucokinase (GCK) by forming an inactive complex with this enzyme (PubMed:23621087, PubMed:23733961). Acts by promoting GCK recruitment to the nucleus, possibly to provide a reserve of GCK that can be quickly released in the cytoplasm after a meal (PubMed:10456334). The affinity of GKRP for GCK is modulated by fructose metabolites: GKRP with bound fructose 6-phosphate has increased affinity for GCK, while GKRP with bound fructose 1-phosphate has strongly decreased affinity for GCK and does not inhibit GCK activity (PubMed:23621087, PubMed:23733961). |
| DCXR | Catalyzes the NADPH-dependent reduction of several pentoses, tetroses, trioses, alpha-dicarbonyl compounds and L-xylulose. Participates in the uronate cycle of glucose metabolism. May play a role in the water absorption and cellular osmoregulation in the proximal renal tubules by producing xylitol, an osmolyte, thereby preventing osmolytic stress from occurring in the renal tubules. |
| AKR7A3 | Can reduce the dialdehyde protein-binding form of aflatoxin B1 (AFB1) to the non-binding AFB1 dialcohol. May be involved in protection of liver against the toxic and carcinogenic effects of AFB1, a potent hepatocarcinogen. |
| A1BG | Involve in immune response-regulating signaling pathway |
| ACY1 | Catalyzes the hydrolysis of N-acetylated amino acids to acetate and free amino acids. |
| BMP1 | Metalloprotease that plays key roles in regulating the formation of the extracellular matrix (ECM) via processing of various precursor proteins into mature functional enzymes or structural proteins (PubMed:33206546). Thereby participates in several developmental and physiological processes such as cartilage and bone formation, muscle growth and homeostasis, wound healing and tissue repair (PubMed:32636307, PubMed:33169406). Roles in ECM formation include cleavage of the C-terminal propeptides from procollagens such as procollagen I, II and III or the proteolytic activation of the enzyme lysyl oxidase LOX, necessary to formation of covalent cross-links in collagen and elastic fibers (PubMed:31152061, PubMed:33206546). Additional substrates include matricellular thrombospondin-1/THBS1 whose cleavage leads to cell adhesion disruption and TGF-beta activation (PubMed:32636307). |
| C4BPA | Controls the classical pathway of complement activation. It binds as a cofactor to C3b/C4b inactivator (C3bINA), which then hydrolyzes the complement fragment C4b. It also accelerates the degradation of the C4bC2a complex (C3 convertase) by dissociating the complement fragment C2a. Alpha chain binds C4b. It also interacts with anticoagulant protein S and with serum amyloid P component. |
| ERBB3 | Tyrosine-protein kinase that plays an essential role as cell surface receptor for neuregulins. Binds to neuregulin-1 (NRG1) and is activated by it; ligand-binding increases phosphorylation on tyrosine residues and promotes its association with the p85 subunit of phosphatidylinositol 3-kinase (PubMed:20682778). May also be activated by CSPG5 (PubMed:15358134). Involved in the regulation of myeloid cell differentiation (PubMed:27416908). |
| F9 | Factor IX is a vitamin K-dependent plasma protein that participates in the intrinsic pathway of blood coagulation by converting factor X to its active form in the presence of Ca2+ ions, phospholipids, and factor VIIIa. |
| FBLN1 | Incorporated into fibronectin-containing matrix fibers. May play a role in cell adhesion and migration along protein fibers within the extracellular matrix (ECM). Could be important for certain developmental processes and contribute to the supramolecular organization of ECM architecture, to those of basement membranes. Has been implicated in a role in cellular transformation and tumor invasion, it appears to be a tumor suppressor. May play a role in haemostasis and thrombosis owing to its ability to bind fibrinogen and incorporate into clots. Could play a significant role in modulating the neurotrophic activities of APP, particularly soluble APP. |
| FTCD | Folate-dependent enzyme, that displays both transferase and deaminase activity. Serves to channel one-carbon units from formiminoglutamate to the folate pool.Binds and promotes bundling of vimentin filaments originating from the Golgi. |
| IGDCC4 | Cell-cell adhesion. |
| IGSF3 | Likely interchromosomal Alu-mediated fusion between IGSF3 on 1p13.1 and GGT on 22q11.2. Breakpoints occurred inside Alu elements as well as in the 5' or 3' ends of them. |
| IL11RA | Receptor for interleukin-11 (IL11). The receptor systems for IL6, LIF, OSM, CNTF, IL11 and CT1 can utilize IL6ST for initiating signal transmission. The IL11/IL11RA/IL6ST complex may be involved in the control of proliferation and/or differentiation of skeletogenic progenitor or other mesenchymal cells (Probable). Essential for the normal development of craniofacial bones and teeth. Restricts suture fusion and tooth number. |
| MREG | Probably functions as a cargo-recognition protein that couples cytoplasmic vesicles to the transport machinery. Plays a role in hair pigmentation, a process that involves shedding of melanosome-containing vesicles from melanocytes, followed by phagocytosis of the melanosome-containing vesicles by keratinocytes. Functions on melanosomes as receptor for RILP and the complex formed by RILP and DCTN1, and thereby contributes to retrograde melanosome transport from the cell periphery to the center. Overexpression causes accumulation of late endosomes and/or lysosomes at the microtubule organising center (MTOC) at the center of the cell. Probably binds cholesterol and requires the presence of cholesterol in membranes to function in microtubule-mediated retrograde organelle transport. Binds phosphatidylinositol 3-phosphate, phosphatidylinositol 4-phosphate, phosphatidylinositol 5-phosphate and phosphatidylinositol 3,5-bisphosphate, but not phosphatidylinositol 3,4-bisphosphate or phosphatidylinositol 4,5-bisphosphate (By similarity). |
| NAB1 | Acts as a transcriptional repressor for zinc finger transcription factors EGR1 and EGR2. |
| ADH1B | Catalyzes the NAD-dependent oxidation of all-trans-retinol and its derivatives such as all-trans-4-hydroxyretinol and may participate in retinoid metabolism. In vitro can also catalyze the NADH-dependent reduction of all-trans-retinal and its derivatives such as all-trans-4-oxoretinal. Catalyzes in the oxidative direction with higher efficiency. Has the same affinity for all-trans-4-hydroxyretinol and all-trans-4-oxoretinal. |
| HTR7 | G-protein coupled receptor for 5-hydroxytryptamine (serotonin), a biogenic hormone that functions as a neurotransmitter, a hormone and a mitogen (PubMed:35714614, PubMed:8226867). Ligand binding causes a conformation change that triggers signaling via guanine nucleotide-binding proteins (G proteins) and modulates the activity of downstream effectors (PubMed:35714614, PubMed:8226867). HTR7 is coupled to G(s) G alpha proteins and mediates activation of adenylate cyclase activity (PubMed:35714614). |
| TK2 | Phosphorylates thymidine, deoxycytidine, and deoxyuridine in the mitochondrial matrix (PubMed:11687801, PubMed:9989599). In non-replicating cells, where cytosolic dNTP synthesis is down-regulated, mtDNA synthesis depends solely on TK2 and DGUOK (PubMed:9989599). Widely used as target of antiviral and chemotherapeutic agents (PubMed:9989599). |

**Supplementary Table 11. Results of Mendelian Randomization analysis from main analysis and alternative methods between modifiable risk factors and CLDs.**

| **Exposure** | **Methods** | **nsnp** | **b** | **se** | ***P* value** | ***P* heterogenicity** | ***P* intercept** |
| --- | --- | --- | --- | --- | --- | --- | --- |
| **MASLD_Ghodsian et al** |  |  |  |  |  |  |  |
| Body mass index | Inverse variance weighted | 516 | 0.52 | 0.06 | 4.33E-21 | 2.73E-04 | - |
| Body mass index | MR Egger | 516 | 0.40 | 0.15 | 0.01 | 2.71E-04 | 0.36 |
| Body mass index | Simple mode | 516 | 0.80 | 0.30 | 0.01 | - | - |
| Body mass index | Weighted median | 516 | 0.51 | 0.09 | 6.37E-09 | - | - |
| Body mass index | Weighted mode | 516 | 0.48 | 0.18 | 0.01 | - | - |
| Waist-hip ratio | Inverse variance weighted | 338 | 0.60 | 0.07 | 6.96E-16 | 1.47E-06 | - |
| Waist-hip ratio | MR Egger | 338 | 0.54 | 0.20 | 0.01 | 1.25E-06 | 0.78 |
| Waist-hip ratio | Simple mode | 338 | 0.33 | 0.29 | 0.26 | - | - |
| Waist-hip ratio | Weighted median | 338 | 0.59 | 0.11 | 1.47E-07 | - | - |
| Waist-hip ratio | Weighted mode | 338 | 0.61 | 0.22 | 0.01 | - | - |
| Lifetime smoking index | Inverse variance weighted | 119 | 0.41 | 0.12 | 3.80E-04 | 0.31 | - |
| Lifetime smoking index | MR Egger | 119 | -0.87 | 0.45 | 0.06 | 0.49 | 4.52E-03 |
| Lifetime smoking index | Simple mode | 119 | 0.10 | 0.50 | 0.84 | - | - |
| Lifetime smoking index | Weighted median | 119 | 0.26 | 0.18 | 0.14 | - | - |
| Lifetime smoking index | Weighted mode | 119 | -0.07 | 0.44 | 0.87 | - | - |
| Smoking initiation | Inverse variance weighted | 187 | 0.26 | 0.07 | 1.66E-04 | 0.21 | - |
| Smoking initiation | MR Egger | 187 | 0.41 | 0.28 | 0.14 | 0.20 | 0.59 |
| Smoking initiation | Simple mode | 187 | 0.18 | 0.30 | 0.56 | - | - |
| Smoking initiation | Weighted median | 187 | 0.20 | 0.10 | 0.05 | - | - |
| Smoking initiation | Weighted mode | 187 | 0.15 | 0.28 | 0.59 | - | - |
| Insomnia | Inverse variance weighted | 150 | 0.13 | 0.04 | 3.88E-04 | 0.18 | - |
| Insomnia | MR Egger | 150 | 0.24 | 0.14 | 0.09 | 0.18 | 0.41 |
| Insomnia | Simple mode | 150 | 0.15 | 0.16 | 0.35 | - | - |
| Insomnia | Weighted median | 150 | 0.13 | 0.05 | 0.01 | - | - |
| Insomnia | Weighted mode | 150 | 0.16 | 0.19 | 0.41 | - | - |
| Problematic alcohol use | Inverse variance weighted | 58 | -0.42 | 0.24 | 0.08 | 4.30E-12 | - |
| Problematic alcohol use | MR Egger | 58 | -1.12 | 0.76 | 0.14 | 6.25E-12 | 0.33 |
| Problematic alcohol use | Simple mode | 58 | -0.04 | 0.48 | 0.94 | - | - |
| Problematic alcohol use | Weighted median | 58 | -0.13 | 0.24 | 0.60 | - | - |
| Problematic alcohol use | Weighted mode | 58 | -0.06 | 0.43 | 0.88 | - | - |
| Drinks per week | Inverse variance weighted | 62 | -0.48 | 0.32 | 0.13 | 1.45E-09 | - |
| Drinks per week | MR Egger | 62 | -1.83 | 0.95 | 0.06 | 4.99E-09 | 0.14 |
| Drinks per week | Simple mode | 62 | 0.48 | 0.70 | 0.49 | - | - |
| Drinks per week | Weighted median | 62 | -0.03 | 0.35 | 0.94 | - | - |
| Drinks per week | Weighted mode | 62 | 0.16 | 0.52 | 0.76 | - | - |
| Fasting insulin | Inverse variance weighted | 32 | 0.77 | 0.36 | 0.03 | 4.84E-09 | - |
| Fasting insulin | MR Egger | 32 | -0.11 | 1.20 | 0.93 | 5.20E-09 | 0.45 |
| Fasting insulin | Simple mode | 32 | 1.88 | 0.58 | 2.72E-03 | - | - |
| Fasting insulin | Weighted median | 32 | 1.25 | 0.34 | 2.18E-04 | - | - |
| Fasting insulin | Weighted mode | 32 | 1.53 | 0.44 | 1.37E-03 | - | - |
| Fasting glucose | Inverse variance weighted | 54 | -0.11 | 0.17 | 0.50 | 1.40E-08 | - |
| Fasting glucose | MR Egger | 54 | -0.40 | 0.29 | 0.18 | 2.52E-08 | 0.24 |
| Fasting glucose | Simple mode | 54 | -0.35 | 0.38 | 0.37 | - | - |
| Fasting glucose | Weighted median | 54 | -0.18 | 0.16 | 0.26 | - | - |
| Fasting glucose | Weighted mode | 54 | -0.20 | 0.15 | 0.19 | - | - |
| Leisure television watching | Inverse variance weighted | 82 | 0.61 | 0.27 | 0.02 | 0.03 | - |
| Leisure television watching | MR Egger | 82 | 0.36 | 0.63 | 0.56 | 0.13 | 0.76 |
| Leisure television watching | Simple mode | 82 | 1.12 | 0.46 | 0.02 | - | - |
| Leisure television watching | Weighted median | 82 | 0.73 | 0.17 | 1.92E-05 | - | - |
| Leisure television watching | Weighted mode | 82 | 1.19 | 0.46 | 0.01 | - | - |
| Relative fat intake | Inverse variance weighted | 6 | 7.86 | 11.55 | 0.50 | 3.25E-04 | - |
| Relative fat intake | MR Egger | 6 | -72.42 | 46.98 | 0.20 | 0.01 | 0.16 |
| Relative fat intake | Simple mode | 6 | 14.39 | 16.40 | 0.42 | - | - |
| Relative fat intake | Weighted median | 6 | 2.80 | 8.43 | 0.74 | - | - |
| Relative fat intake | Weighted mode | 6 | -12.88 | 9.25 | 0.22 | - | - |
| Relative protein intake | Inverse variance weighted | 6 | 4.47 | 15.77 | 0.78 | 4.89E-05 | - |
| Relative protein intake | MR Egger | 6 | -33.59 | 34.95 | 0.39 | 4.91E-04 | 0.29 |
| Relative protein intake | Simple mode | 6 | 3.84 | 15.13 | 0.81 | - | - |
| Relative protein intake | Weighted median | 6 | -0.18 | 8.45 | 0.98 | - | - |
| Relative protein intake | Weighted mode | 6 | -2.59 | 8.57 | 0.77 | - | - |
| Sweet beverage consumption | Inverse variance weighted | 3 | -1.25 | 2.39 | 0.60 | 4.50E-04 | - |
| Sweet beverage consumption | MR Egger | 3 | -33.39 | 11.33 | 0.21 | 0.19 | 0.21 |
| Sweet beverage consumption | Simple mode | 3 | 1.89 | 1.38 | 0.30 | - | - |
| Sweet beverage consumption | Weighted median | 3 | 0.34 | 1.23 | 0.78 | - | - |
| Sweet beverage consumption | Weighted mode | 3 | 1.86 | 1.76 | 0.40 | - | - |
| Two-hour glucose | Inverse variance weighted | 12 | 0.04 | 0.16 | 0.81 | 1.21E-06 | - |
| Two-hour glucose | MR Egger | 12 | -0.63 | 0.39 | 0.14 | 8.48E-05 | 0.09 |
| Two-hour glucose | Simple mode | 12 | -0.19 | 0.19 | 0.33 | - | - |
| Two-hour glucose | Weighted median | 12 | -0.15 | 0.12 | 0.23 | - | - |
| Two-hour glucose | Weighted mode | 12 | -0.22 | 0.14 | 0.14 | - | - |
| Type 2 diabetes | Inverse variance weighted | 37 | 0.06 | 0.05 | 0.24 | 2.32E-14 | - |
| Type 2 diabetes | MR Egger | 37 | -0.06 | 0.14 | 0.64 | 5.51E-14 | 0.31 |
| Type 2 diabetes | Simple mode | 37 | -0.02 | 0.09 | 0.80 | - | - |
| Type 2 diabetes | Weighted median | 37 | -0.06 | 0.04 | 0.20 | - | - |
| Type 2 diabetes | Weighted mode | 37 | -0.08 | 0.05 | 0.11 | - | - |
| Glycated hemoglobin | Inverse variance weighted | 70 | -0.39 | 0.19 | 0.04 | 1.26E-04 | - |
| Glycated hemoglobin | MR Egger | 70 | 0.03 | 0.35 | 0.94 | 2.08E-04 | 0.16 |
| Glycated hemoglobin | Simple mode | 70 | -0.15 | 0.40 | 0.70 | - | - |
| Glycated hemoglobin | Weighted median | 70 | -0.17 | 0.23 | 0.45 | - | - |
| Glycated hemoglobin | Weighted mode | 70 | -0.15 | 0.24 | 0.52 | - | - |
| Body fat percentage | Inverse variance weighted | 10 | 0.46 | 0.28 | 0.10 | 8.53E-04 | - |
| Body fat percentage | MR Egger | 10 | 2.94 | 1.03 | 0.02 | 0.04 | 0.04 |
| Body fat percentage | Simple mode | 10 | 0.20 | 0.54 | 0.72 | - | - |
| Body fat percentage | Weighted median | 10 | 0.27 | 0.28 | 0.33 | - | - |
| Body fat percentage | Weighted mode | 10 | 0.22 | 0.62 | 0.73 | - | - |
| Age of initiation of smoking | Inverse variance weighted | 8 | -0.06 | 0.37 | 0.86 | 0.49 | - |
| Age of initiation of smoking | MR Egger | 8 | -0.78 | 1.87 | 0.69 | 0.39 | 0.71 |
| Age of initiation of smoking | Simple mode | 8 | -0.76 | 0.87 | 0.41 | - | - |
| Age of initiation of smoking | Weighted median | 8 | -0.02 | 0.48 | 0.96 | - | - |
| Age of initiation of smoking | Weighted mode | 8 | -0.85 | 0.92 | 0.38 | - | - |
| Bowls of cereal per week | Inverse variance weighted | 11 | 0.30 | 0.41 | 0.46 | 0.19 | - |
| Bowls of cereal per week | MR Egger | 11 | 2.64 | 1.90 | 0.20 | 0.24 | 0.24 |
| Bowls of cereal per week | Simple mode | 11 | 0.67 | 0.84 | 0.44 | - | - |
| Bowls of cereal per week | Weighted median | 11 | 0.48 | 0.51 | 0.35 | - | - |
| Bowls of cereal per week | Weighted mode | 11 | 1.06 | 0.73 | 0.18 | - | - |
| Cigarettes per day | Inverse variance weighted | 36 | 0.07 | 0.13 | 0.58 | 0.11 | - |
| Cigarettes per day | MR Egger | 36 | -0.19 | 0.22 | 0.41 | 0.14 | 0.16 |
| Cigarettes per day | Simple mode | 36 | -0.05 | 0.39 | 0.89 | - | - |
| Cigarettes per day | Weighted median | 36 | -0.11 | 0.16 | 0.49 | - | - |
| Cigarettes per day | Weighted mode | 36 | -0.08 | 0.15 | 0.58 | - | - |
| Coffee consumption | Inverse variance weighted | 27 | -0.50 | 0.30 | 0.10 | 3.75E-03 | - |
| Coffee consumption | MR Egger | 27 | -0.49 | 0.57 | 0.40 | 2.56E-03 | 0.99 |
| Coffee consumption | Simple mode | 27 | -0.68 | 0.54 | 0.22 | - | - |
| Coffee consumption | Weighted median | 27 | -0.54 | 0.29 | 0.06 | - | - |
| Coffee consumption | Weighted mode | 27 | -0.51 | 0.30 | 0.10 | - | - |
| Glasses of water per day | Inverse variance weighted | 19 | 0.08 | 0.29 | 0.79 | 0.33 | - |
| Glasses of water per day | MR Egger | 19 | 2.15 | 1.85 | 0.26 | 0.35 | 0.27 |
| Glasses of water per day | Simple mode | 19 | -0.48 | 0.72 | 0.51 | - | - |
| Glasses of water per day | Weighted median | 19 | 0.17 | 0.38 | 0.66 | - | - |
| Glasses of water per day | Weighted mode | 19 | 0.11 | 0.61 | 0.87 | - | - |
| Leisure computer use | Inverse variance weighted | 20 | -0.65 | 0.26 | 0.01 | 0.39 | - |
| Leisure computer use | MR Egger | 20 | 0.27 | 2.04 | 0.89 | 0.34 | 0.65 |
| Leisure computer use | Simple mode | 20 | -1.04 | 0.68 | 0.14 | - | - |
| Leisure computer use | Weighted median | 20 | -0.87 | 0.36 | 0.02 | - | - |
| Leisure computer use | Weighted mode | 20 | -1.07 | 0.70 | 0.14 | - | - |
| Glasses of milk intake per week | Wald ratio | - | - | - | - |  | - |
| Overall beef intake | Inverse variance weighted | 2 | -0.30 | 1.81 | 0.87 | 0.03 | - |
| Overall cheese intake | Inverse variance weighted | 21 | -0.13 | 0.30 | 0.67 | 0.13 | - |
| Overall cheese intake | MR Egger | 21 | -0.18 | 1.28 | 0.89 | 0.10 | 0.97 |
| Overall cheese intake | Simple mode | 21 | 0.09 | 0.66 | 0.90 | - | - |
| Overall cheese intake | Weighted median | 21 | -0.08 | 0.37 | 0.83 | - | - |
| Overall cheese intake | Weighted mode | 21 | 0.03 | 0.50 | 0.96 | - | - |
| Overall lamb/mutton intake | Inverse variance weighted | 8 | 0.06 | 0.53 | 0.92 | 0.17 | - |
| Overall lamb/mutton intake | MR Egger | 8 | 1.21 | 3.53 | 0.74 | 0.12 | 0.75 |
| Overall lamb/mutton intake | Simple mode | 8 | -0.27 | 1.01 | 0.80 | - | - |
| Overall lamb/mutton intake | Weighted median | 8 | -0.00003 | 0.60 | 1.00 | - | - |
| Overall lamb/mutton intake | Weighted mode | 8 | -0.12 | 0.78 | 0.88 | - | - |
| Overall non-oily fish intake | Inverse variance weighted | 2 | -0.33 | 0.90 | 0.72 | 0.76 | - |
| Overall non-oily fish intake | MR Egger | - | - | - | - | - | - |
| Overall oily fish intake | Inverse variance weighted | 23 | -0.09 | 0.24 | 0.71 | 0.49 | - |
| Overall oily fish intake | MR Egger | 23 | 0.01 | 0.92 | 0.99 | 0.43 | 0.91 |
| Overall oily fish intake | Simple mode | 23 | -0.24 | 0.62 | 0.71 | - | - |
| Overall oily fish intake | Weighted median | 23 | -0.17 | 0.34 | 0.62 | - | - |
| Overall oily fish intake | Weighted mode | 23 | -0.34 | 0.55 | 0.55 | - | - |
| Overall pork intake | Inverse variance weighted | 4 | -0.17 | 0.63 | 0.79 | 0.85 | - |
| Overall pork intake | MR Egger | 4 | 2.08 | 3.50 | 0.61 | 0.83 | 0.58 |
| Overall pork intake | Simple mode | 4 | 0.34 | 1.03 | 0.76 | - | - |
| Overall pork intake | Weighted median | 4 | -0.19 | 0.71 | 0.79 | - | - |
| Overall pork intake | Weighted mode | 4 | -0.66 | 1.01 | 0.56 | - | - |
| Overall poultry intake | Inverse variance weighted | 3 | 1.12 | 0.75 | 0.14 | 0.93 | - |
| Overall poultry intake | MR Egger | 3 | -3.14 | 12.22 | 0.84 | 0.89 | 0.79 |
| Overall poultry intake | Simple mode | 3 | 1.28 | 1.05 | 0.35 | - | - |
| Overall poultry intake | Weighted median | 3 | 1.14 | 0.91 | 0.21 | - | - |
| Overall poultry intake | Weighted mode | 3 | 1.22 | 0.99 | 0.34 | - | - |
| Overall processed meat intake | Inverse variance weighted | 6 | -0.62 | 0.54 | 0.25 | 0.53 | - |
| Overall processed meat intake | MR Egger | 6 | 1.28 | 3.39 | 0.72 | 0.43 | 0.60 |
| Overall processed meat intake | Simple mode | 6 | -0.58 | 1.00 | 0.58 | - | - |
| Overall processed meat intake | Weighted median | 6 | -0.63 | 0.68 | 0.35 | - | - |
| Overall processed meat intake | Weighted mode | 6 | -0.62 | 0.91 | 0.53 | - | - |
| Pieces of dried fruit per day | Inverse variance weighted | 8 | 0.22 | 0.61 | 0.71 | 0.09 | - |
| Pieces of dried fruit per day | MR Egger | 8 | 4.71 | 2.59 | 0.12 | 0.22 | 0.13 |
| Pieces of dried fruit per day | Simple mode | 8 | 0.73 | 0.90 | 0.45 | - | - |
| Pieces of dried fruit per day | Weighted median | 8 | 0.66 | 0.62 | 0.29 | - | - |
| Pieces of dried fruit per day | Weighted mode | 8 | 0.69 | 0.89 | 0.46 | - | - |
| Pieces of fresh fruit per day | Inverse variance weighted | 32 | -0.19 | 0.22 | 0.41 | 0.05 | - |
| Pieces of fresh fruit per day | MR Egger | 32 | -0.23 | 0.67 | 0.73 | 0.04 | 0.94 |
| Pieces of fresh fruit per day | Simple mode | 32 | 0.26 | 0.53 | 0.62 | - | - |
| Pieces of fresh fruit per day | Weighted median | 32 | 0.13 | 0.30 | 0.66 | - | - |
| Pieces of fresh fruit per day | Weighted mode | 32 | 0.13 | 0.41 | 0.75 | - | - |
| Processed meat consumption | Inverse variance weighted | 4 | -0.45 | 0.59 | 0.44 | 0.75 | - |
| Processed meat consumption | MR Egger | 4 | -1.96 | 3.19 | 0.60 | 0.61 | 0.68 |
| Processed meat consumption | Simple mode | 4 | -0.96 | 0.92 | 0.37 | - | - |
| Processed meat consumption | Weighted median | 4 | -0.68 | 0.68 | 0.32 | - | - |
| Processed meat consumption | Weighted mode | 4 | -0.95 | 0.92 | 0.38 | - | - |
| Proinsulin | Inverse variance weighted | 23 | 0.03 | 0.05 | 0.50 | 0.11 | - |
| Proinsulin | MR Egger | 23 | -0.01 | 0.09 | 0.95 | 0.09 | 0.59 |
| Proinsulin | Simple mode | 23 | 0.05 | 0.12 | 0.67 | - | - |
| Proinsulin | Weighted median | 23 | 0.06 | 0.06 | 0.28 | - | - |
| Proinsulin | Weighted mode | 23 | 0.06 | 0.06 | 0.33 | - | - |
| Relative carbohydrate intake | Inverse variance weighted | 5 | 3.11 | 5.99 | 0.60 | 0.14 | - |
| Relative carbohydrate intake | MR Egger | 5 | -1.27 | 51.16 | 0.98 | 0.08 | 0.94 |
| Relative carbohydrate intake | Simple mode | 5 | 1.13 | 8.93 | 0.91 | - | - |
| Relative carbohydrate intake | Weighted median | 5 | 1.52 | 6.25 | 0.81 | - | - |
| Relative carbohydrate intake | Weighted mode | 5 | 1.22 | 9.14 | 0.90 | - | - |
| Sleep duration | Inverse variance weighted | 56 | -0.003 | 0.003 | 0.27 | 0.02 | - |
| Sleep duration | MR Egger | 56 | -0.01 | 0.01 | 0.38 | 0.02 | 0.53 |
| Sleep duration | Simple mode | 56 | -0.01 | 0.01 | 0.29 | - | - |
| Sleep duration | Weighted median | 56 | -0.002 | 0.004 | 0.65 | - | - |
| Sleep duration | Weighted mode | 56 | 0.01 | 0.01 | 0.32 | - | - |
| Slices of bread per week | Inverse variance weighted | 9 | -0.12 | 0.48 | 0.81 | 0.12 | - |
| Slices of bread per week | MR Egger | 9 | -4.00 | 3.18 | 0.25 | 0.16 | 0.26 |
| Slices of bread per week | Simple mode | 9 | 0.41 | 0.99 | 0.69 | - | - |
| Slices of bread per week | Weighted median | 9 | 0.37 | 0.55 | 0.50 | - | - |
| Slices of bread per week | Weighted mode | 9 | 0.51 | 0.97 | 0.61 | - | - |
| Smoking cessation | Inverse variance weighted | 12 | 0.11 | 0.17 | 0.51 | 0.31 | - |
| Smoking cessation | MR Egger | 12 | -0.77 | 0.49 | 0.15 | 0.52 | 0.09 |
| Smoking cessation | Simple mode | 12 | -0.16 | 0.38 | 0.68 | - | - |
| Smoking cessation | Weighted median | 12 | -0.20 | 0.22 | 0.37 | - | - |
| Smoking cessation | Weighted mode | 12 | -0.22 | 0.24 | 0.37 | - | - |
| Spent driving | Inverse variance weighted | 4 | 0.98 | 0.57 | 0.09 | 0.93 | - |
| Spent driving | MR Egger | 4 | 3.91 | 4.64 | 0.49 | 0.99 | 0.59 |
| Spent driving | Simple mode | 4 | 0.70 | 0.86 | 0.48 | - | - |
| Spent driving | Weighted median | 4 | 0.86 | 0.66 | 0.19 | - | - |
| Spent driving | Weighted mode | 4 | 0.72 | 0.83 | 0.45 | - | - |
| Tablespoons of cooked vegetables per day | Inverse variance weighted | 6 | 0.52 | 0.60 | 0.39 | 0.27 | - |
| Tablespoons of cooked vegetables per day | MR Egger | 6 | 0.53 | 2.16 | 0.82 | 0.17 | 1.00 |
| Tablespoons of cooked vegetables per day | Simple mode | 6 | 1.02 | 1.05 | 0.38 | - | - |
| Tablespoons of cooked vegetables per day | Weighted median | 6 | 0.86 | 0.70 | 0.22 | - | - |
| Tablespoons of cooked vegetables per day | Weighted mode | 6 | 1.15 | 0.92 | 0.27 | - | - |
| Tablespoons of raw vegetables per day | Inverse variance weighted | 7 | -0.95 | 0.53 | 0.07 | 0.20 | - |
| Tablespoons of raw vegetables per day | MR Egger | 7 | 4.70 | 2.34 | 0.10 | 0.78 | 0.06 |
| Tablespoons of raw vegetables per day | Simple mode | 7 | -1.68 | 0.95 | 0.13 | - | - |
| Tablespoons of raw vegetables per day | Weighted median | 7 | -0.86 | 0.61 | 0.16 | - | - |
| Tablespoons of raw vegetables per day | Weighted mode | 7 | -0.70 | 0.79 | 0.41 | - | - |
| Tea consumption | Inverse variance weighted | 10 | -0.69 | 0.50 | 0.17 | 0.14 | - |
| Tea consumption | MR Egger | 10 | -0.60 | 1.54 | 0.70 | 0.10 | 0.95 |
| Tea consumption | Simple mode | 10 | -0.73 | 0.79 | 0.38 | - | - |
| Tea consumption | Weighted median | 10 | -0.72 | 0.55 | 0.19 | - | - |
| Tea consumption | Weighted mode | 10 | -0.80 | 0.61 | 0.22 | - | - |
| Waist circumference | Inverse variance weighted | 33 | 0.03 | 0.34 | 0.93 | 0.01 | - |
| Waist circumference | MR Egger | 33 | 4.25 | 1.76 | 0.02 | 0.03 | 0.02 |
| Waist circumference | Simple mode | 33 | 0.28 | 0.84 | 0.74 | - | - |
| Waist circumference | Weighted median | 33 | 0.39 | 0.41 | 0.34 | - | - |
| Waist circumference | Weighted mode | 33 | 0.52 | 0.68 | 0.45 | - | - |
| **ALD_FinnGen** |  |  |  |  |  |  |  |
| Body mass index | Inverse variance weighted | 530 | 0.08 | 0.09 | 0.36 | 0.09 | - |
| Body mass index | MR Egger | 530 | -0.29 | 0.24 | 0.24 | 0.10 | 0.10 |
| Body mass index | Simple mode | 530 | 0.32 | 0.48 | 0.50 | - | - |
| Body mass index | Weighted median | 530 | 0.06 | 0.15 | 0.68 | - | - |
| Body mass index | Weighted mode | 530 | 0.10 | 0.31 | 0.75 | - | - |
| Waist-hip ratio | Inverse variance weighted | 346 | 0.17 | 0.11 | 0.13 | 0.17 | - |
| Waist-hip ratio | MR Egger | 346 | -0.29 | 0.30 | 0.34 | 0.18 | 0.10 |
| Waist-hip ratio | Simple mode | 346 | 0.39 | 0.52 | 0.45 | - | - |
| Waist-hip ratio | Weighted median | 346 | 0.07 | 0.19 | 0.72 | - | - |
| Waist-hip ratio | Weighted mode | 346 | 0.03 | 0.31 | 0.92 | - | - |
| Lifetime smoking index | Inverse variance weighted | 122 | 0.91 | 0.21 | 2.06E-05 | 0.09 | - |
| Lifetime smoking index | MR Egger | 122 | 0.03 | 0.89 | 0.97 | 0.09 | 0.32 |
| Lifetime smoking index | Simple mode | 122 | 1.14 | 0.85 | 0.18 | - | - |
| Lifetime smoking index | Weighted median | 122 | 0.88 | 0.29 | 0.003 | - | - |
| Lifetime smoking index | Weighted mode | 122 | 0.91 | 0.85 | 0.29 | - | - |
| Smoking initiation | Inverse variance weighted | 192 | 0.70 | 0.13 | 4.68E-08 | 0.03 | - |
| Smoking initiation | MR Egger | 192 | 0.90 | 0.52 | 0.09 | 0.02 | 0.69 |
| Smoking initiation | Simple mode | 192 | 0.98 | 0.51 | 0.06 | - | - |
| Smoking initiation | Weighted median | 192 | 0.70 | 0.17 | 5.13E-05 | - | - |
| Smoking initiation | Weighted mode | 192 | 0.70 | 0.43 | 0.11 | - | - |
| Insomnia | Inverse variance weighted | 154 | 0.22 | 0.06 | 3.39E-04 | 0.20 | - |
| Insomnia | MR Egger | 154 | -0.39 | 0.23 | 0.09 | 0.32 | 0.01 |
| Insomnia | Simple mode | 154 | 0.15 | 0.24 | 0.53 | - | - |
| Insomnia | Weighted median | 154 | 0.18 | 0.09 | 0.05 | - | - |
| Insomnia | Weighted mode | 154 | 0.09 | 0.20 | 0.66 | - | - |
| Problematic alcohol use | Inverse variance weighted | 59 | 1.61 | 0.30 | 7.64E-08 | 0.02 | - |
| Problematic alcohol use | MR Egger | 59 | 1.84 | 0.82 | 0.03 | 0.01 | 0.76 |
| Problematic alcohol use | Simple mode | 59 | 1.55 | 0.82 | 0.06 | - | - |
| Problematic alcohol use | Weighted median | 59 | 1.50 | 0.38 | 9.89E-05 | - | - |
| Problematic alcohol use | Weighted mode | 59 | 1.59 | 0.65 | 0.02 | - | - |
| Drinks per week | Inverse variance weighted | 68 | 1.78 | 0.40 | 7.23E-06 | 0.03 | - |
| Drinks per week | MR Egger | 68 | 2.83 | 1.08 | 0.01 | 0.03 | 0.30 |
| Drinks per week | Simple mode | 68 | 2.32 | 1.21 | 0.06 | - | - |
| Drinks per week | Weighted median | 68 | 1.76 | 0.56 | 1.65E-03 | - | - |
| Drinks per week | Weighted mode | 68 | 2.07 | 0.86 | 0.02 | - | - |
| Fasting insulin | Inverse variance weighted | 33 | -0.09 | 0.35 | 0.80 | 0.81 | - |
| Fasting insulin | MR Egger | 33 | -0.34 | 1.16 | 0.77 | 0.78 | 0.82 |
| Fasting insulin | Simple mode | 33 | 0.95 | 1.08 | 0.39 | - | - |
| Fasting insulin | Weighted median | 33 | 0.13 | 0.53 | 0.80 | - | - |
| Fasting insulin | Weighted mode | 33 | 0.77 | 0.94 | 0.42 | - | - |
| Fasting glucose | Inverse variance weighted | 55 | 0.16 | 0.19 | 0.40 | 0.36 | - |
| Fasting glucose | MR Egger | 55 | 0.22 | 0.34 | 0.51 | 0.33 | 0.82 |
| Fasting glucose | Simple mode | 55 | 0.33 | 0.57 | 0.57 | - | - |
| Fasting glucose | Weighted median | 55 | 0.01 | 0.27 | 0.98 | - | - |
| Fasting glucose | Weighted mode | 55 | 0.05 | 0.27 | 0.86 | - | - |
| Leisure television watching | Inverse variance weighted | 85 | 0.46 | 0.21 | 0.03 | 0.38 | - |
| Leisure television watching | MR Egger | 85 | 1.12 | 1.03 | 0.28 | 0.36 | 0.51 |
| Leisure television watching | Simple mode | 85 | 1.79 | 0.92 | 0.05 | - | - |
| Leisure television watching | Weighted median | 85 | 0.62 | 0.31 | 0.05 | - | - |
| Leisure television watching | Weighted mode | 85 | 1.66 | 0.84 | 0.05 | - | - |
| Relative fat intake | Inverse variance weighted | 7 | -8.64 | 17.00 | 0.61 | 1.96E-03 | - |
| Relative fat intake | MR Egger | 7 | -69.03 | 71.71 | 0.38 | 2.82E-03 | 0.43 |
| Relative fat intake | Simple mode | 7 | 5.74 | 17.73 | 0.76 | - | - |
| Relative fat intake | Weighted median | 7 | -4.10 | 12.22 | 0.74 | - | - |
| Relative fat intake | Weighted mode | 7 | -6.92 | 14.40 | 0.65 | - | - |
| Relative protein intake | Inverse variance weighted | 7 | 11.40 | 19.78 | 0.56 | 0.01 | - |
| Relative protein intake | MR Egger | 7 | 15.02 | 51.02 | 0.78 | 3.86E-03 | 0.94 |
| Relative protein intake | Simple mode | 7 | 5.37 | 20.64 | 0.80 | - | - |
| Relative protein intake | Weighted median | 7 | 18.05 | 14.36 | 0.21 | - | - |
| Relative protein intake | Weighted mode | 7 | 11.22 | 14.40 | 0.47 | - | - |
| Sweet beverage consumption | Inverse variance weighted | 3 | -1.28 | 1.52 | 0.40 | 0.72 | - |
| Sweet beverage consumption | MR Egger | 3 | 3.32 | 15.31 | 0.86 | 0.45 | 0.81 |
| Sweet beverage consumption | Simple mode | 3 | -0.62 | 2.03 | 0.79 | - | - |
| Sweet beverage consumption | Weighted median | 3 | -0.75 | 1.73 | 0.67 | - | - |
| Sweet beverage consumption | Weighted mode | 3 | -0.52 | 2.12 | 0.83 | - | - |
| Two-hour glucose | Inverse variance weighted | 12 | 0.04 | 0.14 | 0.76 | 0.62 | - |
| Two-hour glucose | MR Egger | 12 | 0.48 | 0.36 | 0.22 | 0.69 | 0.22 |
| Two-hour glucose | Simple mode | 12 | 0.28 | 0.28 | 0.35 | - | - |
| Two-hour glucose | Weighted median | 12 | 0.19 | 0.18 | 0.30 | - | - |
| Two-hour glucose | Weighted mode | 12 | 0.18 | 0.26 | 0.49 | - | - |
| Type 2 diabetes | Inverse variance weighted | 38 | -0.003 | 0.05 | 0.96 | 0.13 | - |
| Type 2 diabetes | MR Egger | 38 | 0.06 | 0.14 | 0.66 | 0.11 | 0.62 |
| Type 2 diabetes | Simple mode | 38 | 0.11 | 0.13 | 0.39 | - | - |
| Type 2 diabetes | Weighted median | 38 | 0.02 | 0.07 | 0.82 | - | - |
| Type 2 diabetes | Weighted mode | 38 | 0.04 | 0.10 | 0.68 | - | - |
| Glycated hemoglobin | Inverse variance weighted | 73 | 0.26 | 0.26 | 0.32 | 0.46 | - |
| Glycated hemoglobin | MR Egger | 73 | 0.70 | 0.51 | 0.17 | 0.46 | 0.31 |
| Glycated hemoglobin | Simple mode | 73 | 1.06 | 0.80 | 0.19 | - | - |
| Glycated hemoglobin | Weighted median | 73 | 0.51 | 0.43 | 0.23 | - | - |
| Glycated hemoglobin | Weighted mode | 73 | 0.61 | 0.55 | 0.27 | - | - |
| Body fat percentage | Inverse variance weighted | 10 | 0.13 | 0.28 | 0.65 | 0.54 | - |
| Body fat percentage | MR Egger | 10 | 0.37 | 1.28 | 0.78 | 0.44 | 0.85 |
| Body fat percentage | Simple mode | 10 | 0.47 | 0.60 | 0.45 | - | - |
| Body fat percentage | Weighted median | 10 | 0.06 | 0.39 | 0.87 | - | - |
| Body fat percentage | Weighted mode | 10 | 0.16 | 0.50 | 0.76 | - | - |
| Age of initiation of smoking | Inverse variance weighted | 9 | -1.38 | 0.69 | 0.05 | 0.28 | - |
| Age of initiation of smoking | MR Egger | 9 | -5.17 | 3.48 | 0.18 | 0.30 | 0.30 |
| Age of initiation of smoking | Simple mode | 9 | -1.39 | 1.45 | 0.36 | - | - |
| Age of initiation of smoking | Weighted median | 9 | -1.72 | 0.87 | 0.05 | - | - |
| Age of initiation of smoking | Weighted mode | 9 | -2.12 | 1.47 | 0.19 | - | - |
| Bowls of cereal per week | Inverse variance weighted | 12 | 0.12 | 0.76 | 0.87 | 0.08 | - |
| Bowls of cereal per week | MR Egger | 12 | 3.51 | 3.75 | 0.37 | 0.08 | 0.38 |
| Bowls of cereal per week | Simple mode | 12 | 1.64 | 1.52 | 0.30 | - | - |
| Bowls of cereal per week | Weighted median | 12 | 1.03 | 0.81 | 0.20 | - | - |
| Bowls of cereal per week | Weighted mode | 12 | 1.16 | 1.07 | 0.30 | - | - |
| Cigarettes per day | Inverse variance weighted | 38 | -0.21 | 0.23 | 0.35 | 0.11 | - |
| Cigarettes per day | MR Egger | 38 | -1.20 | 0.35 | 1.38E-03 | 0.49 | 1.29E-03 |
| Cigarettes per day | Simple mode | 38 | 0.16 | 0.74 | 0.83 | - | - |
| Cigarettes per day | Weighted median | 38 | -0.64 | 0.29 | 0.03 | - | - |
| Cigarettes per day | Weighted mode | 38 | -0.67 | 0.27 | 0.02 | - | - |
| Coffee consumption | Inverse variance weighted | 28 | 0.41 | 0.45 | 0.36 | 0.11 | - |
| Coffee consumption | MR Egger | 28 | 0.99 | 0.83 | 0.25 | 0.10 | 0.41 |
| Coffee consumption | Simple mode | 28 | 0.42 | 1.10 | 0.71 | - | - |
| Coffee consumption | Weighted median | 28 | 0.61 | 0.52 | 0.24 | - | - |
| Coffee consumption | Weighted mode | 28 | 0.70 | 0.54 | 0.20 | - | - |
| Glasses of water per day | Inverse variance weighted | 18 | 0.01 | 0.56 | 0.98 | 0.18 | - |
| Glasses of water per day | MR Egger | 18 | 1.92 | 3.68 | 0.61 | 0.15 | 0.61 |
| Glasses of water per day | Simple mode | 18 | -1.00 | 1.51 | 0.52 | - | - |
| Glasses of water per day | Weighted median | 18 | 0.08 | 0.76 | 0.91 | - | - |
| Glasses of water per day | Weighted mode | 18 | -1.00 | 1.54 | 0.52 | - | - |
| Leisure computer use | Inverse variance weighted | 20 | -0.82 | 0.44 | 0.06 | 0.94 | - |
| Leisure computer use | MR Egger | 20 | 0.79 | 3.39 | 0.82 | 0.92 | 0.64 |
| Leisure computer use | Simple mode | 20 | -0.07 | 1.07 | 0.94 | - | - |
| Leisure computer use | Weighted median | 20 | -0.44 | 0.57 | 0.44 | - | - |
| Leisure computer use | Weighted mode | 20 | 0.004 | 1.08 | 1.00 | - | - |
| Glasses of milk intake per week | Wald ratio | 1 | -0.08 | 0.05 | 0.13 | - | - |
| Overall beef intake | Inverse variance weighted | 2 | -0.93 | 1.23 | 0.45 | 0.82 | - |
| Overall cheese intake | Inverse variance weighted | 21 | 0.04 | 0.62 | 0.95 | 0.01 | - |
| Overall cheese intake | MR Egger | 21 | -0.42 | 2.62 | 0.87 | 0.01 | 0.86 |
| Overall cheese intake | Simple mode | 21 | 0.24 | 1.57 | 0.88 | - | - |
| Overall cheese intake | Weighted median | 21 | 0.16 | 0.74 | 0.83 | - | - |
| Overall cheese intake | Weighted mode | 21 | 0.67 | 1.20 | 0.58 | - | - |
| Overall lamb/mutton intake | Inverse variance weighted | 8 | 1.32 | 0.77 | 0.09 | 0.57 | - |
| Overall lamb/mutton intake | MR Egger | 8 | -3.85 | 4.72 | 0.45 | 0.61 | 0.31 |
| Overall lamb/mutton intake | Simple mode | 8 | 0.96 | 1.52 | 0.54 | - | - |
| Overall lamb/mutton intake | Weighted median | 8 | 0.75 | 0.96 | 0.44 | - | - |
| Overall lamb/mutton intake | Weighted mode | 8 | 0.48 | 1.29 | 0.72 | - | - |
| Overall non-oily fish intake | Inverse variance weighted | 2 | 2.44 | 1.69 | 0.15 | 0.30 | - |
| Overall non-oily fish intake | MR Egger | - | - | - | - | - | - |
| Overall oily fish intake | Inverse variance weighted | 21 | 0.02 | 0.46 | 0.97 | 0.33 | - |
| Overall oily fish intake | MR Egger | 21 | 0.39 | 1.88 | 0.84 | 0.28 | 0.84 |
| Overall oily fish intake | Simple mode | 21 | -0.03 | 1.12 | 0.98 | - | - |
| Overall oily fish intake | Weighted median | 21 | -0.03 | 0.65 | 0.96 | - | - |
| Overall oily fish intake | Weighted mode | 21 | 0.001 | 0.95 | 1.00 | - | - |
| Overall pork intake | Inverse variance weighted | 5 | -1.71 | 0.87 | 0.05 | 0.59 | - |
| Overall pork intake | MR Egger | 5 | -0.42 | 2.04 | 0.85 | 0.51 | 0.53 |
| Overall pork intake | Simple mode | 5 | -1.56 | 1.52 | 0.36 | - | - |
| Overall pork intake | Weighted median | 5 | -1.56 | 1.08 | 0.15 | - | - |
| Overall pork intake | Weighted mode | 5 | -1.56 | 1.37 | 0.32 | - | - |
| Overall poultry intake | Inverse variance weighted | 3 | -0.003 | 2.77 | 1.00 | 0.02 | - |
| Overall poultry intake | MR Egger | 3 | -62.83 | 22.12 | 0.22 | 0.64 | 0.22 |
| Overall poultry intake | Simple mode | 3 | 2.95 | 2.59 | 0.37 | - | - |
| Overall poultry intake | Weighted median | 3 | 1.79 | 2.01 | 0.37 | - | - |
| Overall poultry intake | Weighted mode | 3 | 3.02 | 2.87 | 0.40 | - | - |
| Overall processed meat intake | Inverse variance weighted | 6 | 0.68 | 1.25 | 0.59 | 0.12 | - |
| Overall processed meat intake | MR Egger | 6 | -3.10 | 8.53 | 0.73 | 0.08 | 0.68 |
| Overall processed meat intake | Simple mode | 6 | 2.43 | 2.12 | 0.30 | - | - |
| Overall processed meat intake | Weighted median | 6 | 1.03 | 1.24 | 0.41 | - | - |
| Overall processed meat intake | Weighted mode | 6 | 1.75 | 1.99 | 0.42 | - | - |
| Pieces of dried fruit per day | Inverse variance weighted | 10 | -0.69 | 0.72 | 0.34 | 0.51 | - |
| Pieces of dried fruit per day | MR Egger | 10 | 4.27 | 4.65 | 0.39 | 0.52 | 0.31 |
| Pieces of dried fruit per day | Simple mode | 10 | 0.74 | 1.70 | 0.67 | - | - |
| Pieces of dried fruit per day | Weighted median | 10 | -0.27 | 0.95 | 0.78 | - | - |
| Pieces of dried fruit per day | Weighted mode | 10 | 1.01 | 1.62 | 0.55 | - | - |
| Pieces of fresh fruit per day | Inverse variance weighted | 37 | -0.62 | 0.37 | 0.09 | 0.04 | - |
| Pieces of fresh fruit per day | MR Egger | 37 | -3.50 | 1.06 | 2.30E-03 | 0.19 | 0.01 |
| Pieces of fresh fruit per day | Simple mode | 37 | 0.57 | 1.16 | 0.63 | - | - |
| Pieces of fresh fruit per day | Weighted median | 37 | -0.72 | 0.52 | 0.16 | - | - |
| Pieces of fresh fruit per day | Weighted mode | 37 | -0.54 | 0.73 | 0.46 | - | - |
| Processed meat consumption | Inverse variance weighted | 4 | -0.49 | 1.04 | 0.64 | 0.38 | - |
| Processed meat consumption | MR Egger | 4 | 4.10 | 5.99 | 0.56 | 0.31 | 0.52 |
| Processed meat consumption | Simple mode | 4 | 0.59 | 1.91 | 0.78 | - | - |
| Processed meat consumption | Weighted median | 4 | 0.03 | 1.25 | 0.98 | - | - |
| Processed meat consumption | Weighted mode | 4 | 0.61 | 1.98 | 0.78 | - | - |
| Proinsulin | Inverse variance weighted | 24 | 0.09 | 0.08 | 0.26 | 0.23 | - |
| Proinsulin | MR Egger | 24 | 0.08 | 0.14 | 0.57 | 0.19 | 0.94 |
| Proinsulin | Simple mode | 24 | -0.08 | 0.15 | 0.58 | - | - |
| Proinsulin | Weighted median | 24 | 0.09 | 0.09 | 0.37 | - | - |
| Proinsulin | Weighted mode | 24 | 0.07 | 0.09 | 0.44 | - | - |
| Relative carbohydrate intake | Inverse variance weighted | 5 | -2.96 | 8.04 | 0.71 | 0.35 | - |
| Relative carbohydrate intake | MR Egger | 5 | 51.00 | 58.28 | 0.45 | 0.33 | 0.42 |
| Relative carbohydrate intake | Simple mode | 5 | 0.89 | 14.21 | 0.95 | - | - |
| Relative carbohydrate intake | Weighted median | 5 | 0.72 | 9.26 | 0.94 | - | - |
| Relative carbohydrate intake | Weighted mode | 5 | 4.71 | 12.47 | 0.72 | - | - |
| Sleep duration | Inverse variance weighted | 57 | -0.001 | 0.01 | 0.93 | 2.95E-03 | - |
| Sleep duration | MR Egger | 57 | 0.03 | 0.02 | 0.24 | 3.84E-03 | 0.21 |
| Sleep duration | Simple mode | 57 | 0.01 | 0.02 | 0.55 | - | - |
| Sleep duration | Weighted median | 57 | 0.01 | 0.01 | 0.32 | - | - |
| Sleep duration | Weighted mode | 57 | 0.01 | 0.01 | 0.42 | - | - |
| Slices of bread per week | Inverse variance weighted | 10 | -0.79 | 0.66 | 0.23 | 0.31 | - |
| Slices of bread per week | MR Egger | 10 | 3.68 | 4.28 | 0.42 | 0.32 | 0.32 |
| Slices of bread per week | Simple mode | 10 | -0.64 | 1.46 | 0.67 | - | - |
| Slices of bread per week | Weighted median | 10 | -0.56 | 0.87 | 0.52 | - | - |
| Slices of bread per week | Weighted mode | 10 | -0.34 | 1.44 | 0.82 | - | - |
| Smoking cessation | Inverse variance weighted | 13 | 0.25 | 0.27 | 0.36 | 0.79 | - |
| Smoking cessation | MR Egger | 13 | 1.10 | 0.78 | 0.19 | 0.83 | 0.27 |
| Smoking cessation | Simple mode | 13 | -0.18 | 0.68 | 0.80 | - | - |
| Smoking cessation | Weighted median | 13 | 0.35 | 0.38 | 0.36 | - | - |
| Smoking cessation | Weighted mode | 13 | -0.28 | 0.61 | 0.65 | - | - |
| Spent driving | Inverse variance weighted | 4 | -0.66 | 1.26 | 0.60 | 0.19 | - |
| Spent driving | MR Egger | 4 | 14.84 | 7.87 | 0.20 | 0.65 | 0.19 |
| Spent driving | Simple mode | 4 | -0.86 | 2.01 | 0.70 | - | - |
| Spent driving | Weighted median | 4 | -0.89 | 1.30 | 0.49 | - | - |
| Spent driving | Weighted mode | 4 | -0.65 | 1.90 | 0.76 | - | - |
| Tablespoons of cooked vegetables per day | Inverse variance weighted | 7 | 0.41 | 0.86 | 0.63 | 0.49 | - |
| Tablespoons of cooked vegetables per day | MR Egger | 7 | 0.48 | 3.72 | 0.90 | 0.36 | 0.99 |
| Tablespoons of cooked vegetables per day | Simple mode | 7 | 0.34 | 1.68 | 0.85 | - | - |
| Tablespoons of cooked vegetables per day | Weighted median | 7 | 0.48 | 1.12 | 0.67 | - | - |
| Tablespoons of cooked vegetables per day | Weighted mode | 7 | 0.60 | 1.54 | 0.71 | - | - |
| Tablespoons of raw vegetables per day | Inverse variance weighted | 8 | -0.19 | 0.93 | 0.84 | 0.10 | - |
| Tablespoons of raw vegetables per day | MR Egger | 8 | 7.65 | 3.97 | 0.10 | 0.31 | 0.09 |
| Tablespoons of raw vegetables per day | Simple mode | 8 | -1.50 | 1.53 | 0.36 | - | - |
| Tablespoons of raw vegetables per day | Weighted median | 8 | -0.65 | 0.99 | 0.51 | - | - |
| Tablespoons of raw vegetables per day | Weighted mode | 8 | -0.98 | 1.39 | 0.50 | - | - |
| Tea consumption | Inverse variance weighted | 11 | -0.14 | 0.93 | 0.88 | 0.06 | - |
| Tea consumption | MR Egger | 11 | 3.85 | 2.49 | 0.16 | 0.15 | 0.12 |
| Tea consumption | Simple mode | 11 | 0.33 | 1.60 | 0.84 | - | - |
| Tea consumption | Weighted median | 11 | 0.70 | 0.97 | 0.47 | - | - |
| Tea consumption | Weighted mode | 11 | 0.86 | 1.02 | 0.42 | - | - |
| Waist circumference | Inverse variance weighted | 39 | 0.26 | 0.39 | 0.50 | 0.53 | - |
| Waist circumference | MR Egger | 39 | -0.84 | 1.20 | 0.49 | 0.53 | 0.34 |
| Waist circumference | Simple mode | 39 | 0.27 | 0.83 | 0.75 | - | - |
| Waist circumference | Weighted median | 39 | -0.11 | 0.59 | 0.86 | - | - |
| Waist circumference | Weighted mode | 39 | -0.10 | 0.60 | 0.87 | - | - |
| **Cirrhosis_Wong et al** |  |  |  |  |  |  |  |
| Body mass index | Inverse variance weighted | 543 | 0.40 | 0.08 | 3.28E-07 | 0.07 | - |
| Body mass index | MR Egger | 543 | 0.28 | 0.21 | 0.18 | 0.06 | 0.55 |
| Body mass index | Simple mode | 543 | 0.31 | 0.40 | 0.44 | - | - |
| Body mass index | Weighted median | 543 | 0.25 | 0.13 | 0.05 | - | - |
| Body mass index | Weighted mode | 543 | 0.28 | 0.24 | 0.25 | - | - |
| Waist-hip ratio | Inverse variance weighted | 356 | 0.54 | 0.10 | 9.39E-08 | 0.03 | - |
| Waist-hip ratio | MR Egger | 356 | 0.72 | 0.28 | 0.01 | 0.03 | 0.50 |
| Waist-hip ratio | Simple mode | 356 | 0.41 | 0.48 | 0.40 | - | - |
| Waist-hip ratio | Weighted median | 356 | 0.51 | 0.16 | 1.10E-03 | - | - |
| Waist-hip ratio | Weighted mode | 356 | 0.38 | 0.42 | 0.37 | - | - |
| Lifetime smoking index | Inverse variance weighted | 124 | 0.69 | 0.18 | 1.72E-04 | 0.11 | - |
| Lifetime smoking index | MR Egger | 124 | -0.15 | 0.73 | 0.84 | 0.11 | 0.24 |
| Lifetime smoking index | Simple mode | 124 | 0.74 | 0.71 | 0.30 | - | - |
| Lifetime smoking index | Weighted median | 124 | 0.72 | 0.27 | 0.01 | - | - |
| Lifetime smoking index | Weighted mode | 124 | 0.61 | 0.55 | 0.27 | - | - |
| Smoking initiation | Inverse variance weighted | 201 | 0.20 | 0.10 | 0.05 | 0.50 | - |
| Smoking initiation | MR Egger | 201 | 0.09 | 0.40 | 0.82 | 0.48 | 0.78 |
| Smoking initiation | Simple mode | 201 | 0.89 | 0.44 | 0.05 | - | - |
| Smoking initiation | Weighted median | 201 | 0.18 | 0.15 | 0.24 | - | - |
| Smoking initiation | Weighted mode | 201 | 0.31 | 0.38 | 0.42 | - | - |
| Insomnia | Inverse variance weighted | 158 | 0.04 | 0.06 | 0.52 | 0.02 | - |
| Insomnia | MR Egger | 158 | 0.16 | 0.22 | 0.48 | 0.02 | 0.58 |
| Insomnia | Simple mode | 158 | -0.02 | 0.24 | 0.94 | - | - |
| Insomnia | Weighted median | 158 | 0.02 | 0.08 | 0.80 | - | - |
| Insomnia | Weighted mode | 158 | 0.05 | 0.20 | 0.81 | - | - |
| Problematic alcohol use | Inverse variance weighted | 61 | 0.16 | 0.33 | 0.62 | 3.52E-15 | - |
| Problematic alcohol use | MR Egger | 61 | 0.22 | 0.61 | 0.72 | 1.97E-15 | 0.91 |
| Problematic alcohol use | Simple mode | 61 | 1.44 | 0.74 | 0.06 | - | - |
| Problematic alcohol use | Weighted median | 61 | 0.74 | 0.32 | 0.02 | - | - |
| Problematic alcohol use | Weighted mode | 61 | 0.81 | 0.33 | 0.02 | - | - |
| Drinks per week | Inverse variance weighted | 71 | 0.41 | 0.44 | 0.35 | 4.73E-13 | - |
| Drinks per week | MR Egger | 71 | 0.05 | 0.88 | 0.95 | 3.43E-13 | 0.64 |
| Drinks per week | Simple mode | 71 | -0.05 | 0.98 | 0.96 | - | - |
| Drinks per week | Weighted median | 71 | 1.05 | 0.44 | 0.02 | - | - |
| Drinks per week | Weighted mode | 71 | 0.87 | 0.51 | 0.09 | - | - |
| Fasting insulin | Inverse variance weighted | 33 | 1.50 | 0.32 | 3.51E-06 | 0.35 | - |
| Fasting insulin | MR Egger | 33 | 2.91 | 1.03 | 0.01 | 0.40 | 0.16 |
| Fasting insulin | Simple mode | 33 | 1.69 | 0.90 | 0.07 | - | - |
| Fasting insulin | Weighted median | 33 | 1.63 | 0.45 | 2.75E-04 | - | - |
| Fasting insulin | Weighted mode | 33 | 1.60 | 0.77 | 0.04 | - | - |
| Fasting glucose | Inverse variance weighted | 58 | -0.14 | 0.17 | 0.41 | 0.39 | - |
| Fasting glucose | MR Egger | 58 | -0.001 | 0.30 | 1.00 | 0.36 | 0.58 |
| Fasting glucose | Simple mode | 58 | -0.08 | 0.51 | 0.88 | - | - |
| Fasting glucose | Weighted median | 58 | 0.16 | 0.24 | 0.52 | - | - |
| Fasting glucose | Weighted mode | 58 | 0.11 | 0.24 | 0.67 | - | - |
| Leisure television watching | Inverse variance weighted | 93 | 0.59 | 0.21 | 4.47E-03 | 0.02 | - |
| Leisure television watching | MR Egger | 93 | 2.01 | 1.05 | 0.06 | 0.02 | 0.17 |
| Leisure television watching | Simple mode | 93 | 0.86 | 0.66 | 0.20 | - | - |
| Leisure television watching | Weighted median | 93 | 0.61 | 0.27 | 0.02 | - | - |
| Leisure television watching | Weighted mode | 93 | 0.86 | 0.61 | 0.16 | - | - |
| Relative fat intake | Inverse variance weighted | 7 | -3.35 | 17.49 | 0.85 | 1.89E-05 | - |
| Relative fat intake | MR Egger | 7 | -57.01 | 45.83 | 0.27 | 2.11E-04 | 0.26 |
| Relative fat intake | Simple mode | 7 | 19.30 | 25.38 | 0.48 | - | - |
| Relative fat intake | Weighted median | 7 | -7.46 | 11.59 | 0.52 | - | - |
| Relative fat intake | Weighted mode | 7 | -9.71 | 14.67 | 0.53 | - | - |
| Relative protein intake | Inverse variance weighted | 7 | -9.08 | 12.24 | 0.46 | 0.21 | - |
| Relative protein intake | MR Egger | 7 | 5.77 | 29.97 | 0.85 | 0.16 | 0.61 |
| Relative protein intake | Simple mode | 7 | -32.77 | 20.09 | 0.15 | - | - |
| Relative protein intake | Weighted median | 7 | -8.97 | 12.39 | 0.47 | - | - |
| Relative protein intake | Weighted mode | 7 | -5.92 | 12.56 | 0.65 | - | - |
| Sweet beverage consumption | Inverse variance weighted | 3 | -0.77 | 1.33 | 0.56 | 0.57 | - |
| Sweet beverage consumption | MR Egger | 3 | -4.21 | 13.78 | 0.81 | 0.30 | 0.84 |
| Sweet beverage consumption | Simple mode | 3 | -1.59 | 2.09 | 0.53 | - | - |
| Sweet beverage consumption | Weighted median | 3 | -1.52 | 1.62 | 0.35 | - | - |
| Sweet beverage consumption | Weighted mode | 3 | -1.64 | 2.00 | 0.50 | - | - |
| Two-hour glucose | Inverse variance weighted | 14 | 0.20 | 0.13 | 0.13 | 0.16 | - |
| Two-hour glucose | MR Egger | 14 | 0.34 | 0.38 | 0.38 | 0.13 | 0.70 |
| Two-hour glucose | Simple mode | 14 | 0.63 | 0.31 | 0.07 | - | - |
| Two-hour glucose | Weighted median | 14 | 0.25 | 0.18 | 0.16 | - | - |
| Two-hour glucose | Weighted mode | 14 | 0.44 | 0.27 | 0.13 | - | - |
| Type 2 diabetes | Inverse variance weighted | 39 | -0.04 | 0.07 | 0.63 | 1.22E-10 | - |
| Type 2 diabetes | MR Egger | 39 | -0.12 | 0.18 | 0.53 | 8.66E-11 | 0.64 |
| Type 2 diabetes | Simple mode | 39 | -0.01 | 0.14 | 0.91 | - | - |
| Type 2 diabetes | Weighted median | 39 | -0.16 | 0.07 | 0.02 | - | - |
| Type 2 diabetes | Weighted mode | 39 | -0.15 | 0.08 | 0.09 | - | - |
| Glycated hemoglobin | Inverse variance weighted | 75 | -0.77 | 0.27 | 3.67E-03 | 3.11E-03 | - |
| Glycated hemoglobin | MR Egger | 75 | -0.53 | 0.49 | 0.28 | 2.72E-03 | 0.56 |
| Glycated hemoglobin | Simple mode | 75 | -0.81 | 0.86 | 0.35 | - | - |
| Glycated hemoglobin | Weighted median | 75 | 0.07 | 0.35 | 0.84 | - | - |
| Glycated hemoglobin | Weighted mode | 75 | 0.15 | 0.40 | 0.71 | - | - |
| Body fat percentage | Inverse variance weighted | 10 | 0.37 | 0.34 | 0.27 | 0.06 | - |
| Body fat percentage | MR Egger | 10 | 2.42 | 1.48 | 0.14 | 0.10 | 0.19 |
| Body fat percentage | Simple mode | 10 | 0.29 | 0.57 | 0.63 | - | - |
| Body fat percentage | Weighted median | 10 | 0.41 | 0.34 | 0.23 | - | - |
| Body fat percentage | Weighted mode | 10 | 0.35 | 0.46 | 0.47 | - | - |
| Age of initiation of smoking | Inverse variance weighted | 9 | -0.57 | 0.64 | 0.37 | 0.21 | - |
| Age of initiation of smoking | MR Egger | 9 | 2.18 | 3.44 | 0.55 | 0.19 | 0.44 |
| Age of initiation of smoking | Simple mode | 9 | -0.04 | 1.23 | 0.98 | - | - |
| Age of initiation of smoking | Weighted median | 9 | -0.69 | 0.76 | 0.37 | - | - |
| Age of initiation of smoking | Weighted mode | 9 | -0.54 | 1.11 | 0.64 | - | - |
| Bowls of cereal per week | Inverse variance weighted | 13 | -0.16 | 0.48 | 0.74 | 0.84 | - |
| Bowls of cereal per week | MR Egger | 13 | -0.11 | 1.86 | 0.95 | 0.77 | 0.98 |
| Bowls of cereal per week | Simple mode | 13 | -1.19 | 1.08 | 0.29 | - | - |
| Bowls of cereal per week | Weighted median | 13 | -0.52 | 0.67 | 0.44 | - | - |
| Bowls of cereal per week | Weighted mode | 13 | -0.89 | 1.04 | 0.41 | - | - |
| Cigarettes per day | Inverse variance weighted | 38 | 0.17 | 0.18 | 0.33 | 0.57 | - |
| Cigarettes per day | MR Egger | 38 | 0.24 | 0.31 | 0.45 | 0.53 | 0.80 |
| Cigarettes per day | Simple mode | 38 | -0.16 | 0.57 | 0.78 | - | - |
| Cigarettes per day | Weighted median | 38 | 0.25 | 0.25 | 0.31 | - | - |
| Cigarettes per day | Weighted mode | 38 | 0.31 | 0.26 | 0.23 | - | - |
| Coffee consumption | Inverse variance weighted | 30 | -0.14 | 0.33 | 0.67 | 0.82 | - |
| Coffee consumption | MR Egger | 30 | -0.05 | 0.62 | 0.94 | 0.78 | 0.86 |
| Coffee consumption | Simple mode | 30 | 0.72 | 0.87 | 0.42 | - | - |
| Coffee consumption | Weighted median | 30 | -0.01 | 0.46 | 0.98 | - | - |
| Coffee consumption | Weighted mode | 30 | 0.14 | 0.47 | 0.77 | - | - |
| Glasses of water per day | Inverse variance weighted | 20 | 0.48 | 0.48 | 0.33 | 0.16 | - |
| Glasses of water per day | MR Egger | 20 | 3.49 | 3.14 | 0.28 | 0.16 | 0.34 |
| Glasses of water per day | Simple mode | 20 | 1.55 | 1.19 | 0.21 | - | - |
| Glasses of water per day | Weighted median | 20 | 0.88 | 0.62 | 0.16 | - | - |
| Glasses of water per day | Weighted mode | 20 | 1.70 | 1.10 | 0.14 | - | - |
| Leisure computer use | Inverse variance weighted | 21 | -0.44 | 0.53 | 0.41 | 0.01 | - |
| Leisure computer use | MR Egger | 21 | 0.23 | 4.36 | 0.96 | 0.01 | 0.88 |
| Leisure computer use | Simple mode | 21 | -1.43 | 1.18 | 0.24 | - | - |
| Leisure computer use | Weighted median | 21 | -1.20 | 0.58 | 0.04 | - | - |
| Leisure computer use | Weighted mode | 21 | -1.55 | 0.99 | 0.13 | - | - |
| Glasses of milk intake per week | Wald ratio | 1 | -0.002 | 0.05 | 0.97 | - | - |
| Overall beef intake | Inverse variance weighted | 2 | 0.15 | 2.72 | 0.96 | 0.07 | - |
| Overall cheese intake | Inverse variance weighted | 24 | -0.14 | 0.39 | 0.72 | 0.63 | - |
| Overall cheese intake | MR Egger | 24 | 1.80 | 1.70 | 0.30 | 0.65 | 0.25 |
| Overall cheese intake | Simple mode | 24 | 0.63 | 0.88 | 0.48 | - | - |
| Overall cheese intake | Weighted median | 24 | 0.38 | 0.54 | 0.48 | - | - |
| Overall cheese intake | Weighted mode | 24 | 0.59 | 0.76 | 0.44 | - | - |
| Overall lamb/mutton intake | Inverse variance weighted | 8 | -0.91 | 0.68 | 0.18 | 0.89 | - |
| Overall lamb/mutton intake | MR Egger | 8 | -3.06 | 4.16 | 0.49 | 0.85 | 0.62 |
| Overall lamb/mutton intake | Simple mode | 8 | -1.65 | 1.32 | 0.25 | - | - |
| Overall lamb/mutton intake | Weighted median | 8 | -0.92 | 0.84 | 0.27 | - | - |
| Overall lamb/mutton intake | Weighted mode | 8 | -1.28 | 1.14 | 0.30 | - | - |
| Overall non-oily fish intake | Inverse variance weighted | 2 | -0.53 | 1.58 | 0.74 | 0.26 | - |
| Overall non-oily fish intake | MR Egger | - | - | - | - | - | - |
| Overall oily fish intake | Inverse variance weighted | 25 | 0.49 | 0.44 | 0.27 | 0.09 | - |
| Overall oily fish intake | MR Egger | 25 | -1.09 | 1.70 | 0.53 | 0.09 | 0.35 |
| Overall oily fish intake | Simple mode | 25 | 0.64 | 0.99 | 0.52 | - | - |
| Overall oily fish intake | Weighted median | 25 | 0.47 | 0.54 | 0.38 | - | - |
| Overall oily fish intake | Weighted mode | 25 | 0.51 | 0.88 | 0.57 | - | - |
| Overall pork intake | Inverse variance weighted | 5 | -1.40 | 1.26 | 0.27 | 0.10 | - |
| Overall pork intake | MR Egger | 5 | -7.28 | 2.66 | 0.07 | 0.55 | 0.10 |
| Overall pork intake | Simple mode | 5 | -0.19 | 1.67 | 0.92 | - | - |
| Overall pork intake | Weighted median | 5 | -0.46 | 1.17 | 0.69 | - | - |
| Overall pork intake | Weighted mode | 5 | 0.01 | 1.47 | 0.99 | - | - |
| Overall poultry intake | Inverse variance weighted | 3 | 0.56 | 1.13 | 0.62 | 0.97 | - |
| Overall poultry intake | MR Egger | 3 | -2.52 | 18.30 | 0.91 | 0.88 | 0.89 |
| Overall poultry intake | Simple mode | 3 | 0.42 | 1.52 | 0.81 | - | - |
| Overall poultry intake | Weighted median | 3 | 0.47 | 1.29 | 0.71 | - | - |
| Overall poultry intake | Weighted mode | 3 | 0.40 | 1.49 | 0.81 | - | - |
| Overall processed meat intake | Inverse variance weighted | 6 | -0.12 | 0.83 | 0.88 | 0.86 | - |
| Overall processed meat intake | MR Egger | 6 | -2.67 | 5.22 | 0.64 | 0.79 | 0.65 |
| Overall processed meat intake | Simple mode | 6 | -0.95 | 1.50 | 0.55 | - | - |
| Overall processed meat intake | Weighted median | 6 | -0.61 | 0.99 | 0.54 | - | - |
| Overall processed meat intake | Weighted mode | 6 | -1.00 | 1.58 | 0.55 | - | - |
| Pieces of dried fruit per day | Inverse variance weighted | 11 | -0.80 | 0.63 | 0.20 | 0.93 | - |
| Pieces of dried fruit per day | MR Egger | 11 | 0.43 | 3.27 | 0.90 | 0.90 | 0.71 |
| Pieces of dried fruit per day | Simple mode | 11 | -0.70 | 1.18 | 0.57 | - | - |
| Pieces of dried fruit per day | Weighted median | 11 | -0.82 | 0.83 | 0.33 | - | - |
| Pieces of dried fruit per day | Weighted mode | 11 | -0.74 | 1.19 | 0.55 | - | - |
| Pieces of fresh fruit per day | Inverse variance weighted | 37 | -0.21 | 0.27 | 0.45 | 0.70 | - |
| Pieces of fresh fruit per day | MR Egger | 37 | -0.01 | 0.84 | 0.99 | 0.65 | 0.80 |
| Pieces of fresh fruit per day | Simple mode | 37 | -0.33 | 0.73 | 0.66 | - | - |
| Pieces of fresh fruit per day | Weighted median | 37 | -0.27 | 0.41 | 0.50 | - | - |
| Pieces of fresh fruit per day | Weighted mode | 37 | -0.19 | 0.52 | 0.72 | - | - |
| Processed meat consumption | Inverse variance weighted | 4 | -0.16 | 0.88 | 0.86 | 0.99 | - |
| Processed meat consumption | MR Egger | 4 | -1.57 | 4.65 | 0.77 | 0.99 | 0.79 |
| Processed meat consumption | Simple mode | 4 | -0.34 | 1.27 | 0.80 | - | - |
| Processed meat consumption | Weighted median | 4 | -0.25 | 0.98 | 0.80 | - | - |
| Processed meat consumption | Weighted mode | 4 | -0.34 | 1.34 | 0.82 | - | - |
| Proinsulin | Inverse variance weighted | 25 | 0.12 | 0.07 | 0.07 | 0.37 | - |
| Proinsulin | MR Egger | 25 | 0.13 | 0.11 | 0.28 | 0.32 | 0.92 |
| Proinsulin | Simple mode | 25 | 0.22 | 0.17 | 0.20 | - | - |
| Proinsulin | Weighted median | 25 | 0.20 | 0.09 | 0.03 | - | - |
| Proinsulin | Weighted mode | 25 | 0.20 | 0.08 | 0.03 | - | - |
| Relative carbohydrate intake | Inverse variance weighted | 5 | 9.72 | 7.08 | 0.17 | 0.95 | - |
| Relative carbohydrate intake | MR Egger | 5 | 16.05 | 52.40 | 0.78 | 0.87 | 0.91 |
| Relative carbohydrate intake | Simple mode | 5 | 12.22 | 10.46 | 0.31 | - | - |
| Relative carbohydrate intake | Weighted median | 5 | 10.99 | 8.46 | 0.19 | - | - |
| Relative carbohydrate intake | Weighted mode | 5 | 11.81 | 9.93 | 0.30 | - | - |
| Sleep duration | Inverse variance weighted | 63 | -0.004 | 0.004 | 0.25 | 0.74 | - |
| Sleep duration | MR Egger | 63 | 0.001 | 0.01 | 0.94 | 0.71 | 0.70 |
| Sleep duration | Simple mode | 63 | -0.01 | 0.01 | 0.47 | - | - |
| Sleep duration | Weighted median | 63 | -0.01 | 0.01 | 0.36 | - | - |
| Sleep duration | Weighted mode | 63 | -0.004 | 0.01 | 0.72 | - | - |
| Slices of bread per week | Inverse variance weighted | 12 | 0.81 | 0.55 | 0.14 | 0.95 | - |
| Slices of bread per week | MR Egger | 12 | -3.13 | 3.66 | 0.41 | 0.97 | 0.30 |
| Slices of bread per week | Simple mode | 12 | 0.61 | 1.02 | 0.56 | - | - |
| Slices of bread per week | Weighted median | 12 | 0.69 | 0.73 | 0.35 | - | - |
| Slices of bread per week | Weighted mode | 12 | 0.21 | 0.94 | 0.83 | - | - |
| Smoking cessation | Inverse variance weighted | 15 | 0.04 | 0.22 | 0.85 | 0.57 | - |
| Smoking cessation | MR Egger | 15 | 0.17 | 0.61 | 0.79 | 0.50 | 0.83 |
| Smoking cessation | Simple mode | 15 | 0.06 | 0.59 | 0.93 | - | - |
| Smoking cessation | Weighted median | 15 | -0.06 | 0.29 | 0.84 | - | - |
| Smoking cessation | Weighted mode | 15 | -0.28 | 0.45 | 0.55 | - | - |
| Spent driving | Inverse variance weighted | 4 | 0.52 | 0.90 | 0.56 | 0.99 | - |
| Spent driving | MR Egger | 4 | 1.25 | 7.37 | 0.88 | 0.93 | 0.93 |
| Spent driving | Simple mode | 4 | 0.25 | 1.38 | 0.87 | - | - |
| Spent driving | Weighted median | 4 | 0.46 | 0.98 | 0.64 | - | - |
| Spent driving | Weighted mode | 4 | 0.27 | 1.37 | 0.86 | - | - |
| Tablespoons of cooked vegetables per day | Inverse variance weighted | 8 | 1.55 | 1.18 | 0.19 | 0.04 | - |
| Tablespoons of cooked vegetables per day | MR Egger | 8 | 3.33 | 4.55 | 0.49 | 0.03 | 0.70 |
| Tablespoons of cooked vegetables per day | Simple mode | 8 | 0.19 | 1.82 | 0.92 | - | - |
| Tablespoons of cooked vegetables per day | Weighted median | 8 | 0.67 | 1.13 | 0.55 | - | - |
| Tablespoons of cooked vegetables per day | Weighted mode | 8 | -0.04 | 1.63 | 0.98 | - | - |
| Tablespoons of raw vegetables per day | Inverse variance weighted | 9 | -0.03 | 0.66 | 0.96 | 0.47 | - |
| Tablespoons of raw vegetables per day | MR Egger | 9 | -4.18 | 3.42 | 0.26 | 0.53 | 0.26 |
| Tablespoons of raw vegetables per day | Simple mode | 9 | -0.95 | 1.36 | 0.50 | - | - |
| Tablespoons of raw vegetables per day | Weighted median | 9 | -0.27 | 0.88 | 0.76 | - | - |
| Tablespoons of raw vegetables per day | Weighted mode | 9 | -0.52 | 1.16 | 0.66 | - | - |
| Tea consumption | Inverse variance weighted | 13 | -0.03 | 0.72 | 0.96 | 0.11 | - |
| Tea consumption | MR Egger | 13 | -1.56 | 2.22 | 0.50 | 0.10 | 0.48 |
| Tea consumption | Simple mode | 13 | -0.95 | 1.07 | 0.39 | - | - |
| Tea consumption | Weighted median | 13 | -0.68 | 0.76 | 0.37 | - | - |
| Tea consumption | Weighted mode | 13 | -0.56 | 0.89 | 0.54 | - | - |
| Waist circumference | Inverse variance weighted | 40 | -0.02 | 0.40 | 0.95 | 0.20 | - |
| Waist circumference | MR Egger | 40 | 3.28 | 1.89 | 0.09 | 0.28 | 0.08 |
| Waist circumference | Simple mode | 40 | -0.68 | 0.97 | 0.49 | - | - |
| Waist circumference | Weighted median | 40 | 0.03 | 0.54 | 0.95 | - | - |
| Waist circumference | Weighted mode | 40 | -0.20 | 0.78 | 0.80 | - | - |
| **Liver cancer_FinnGen** |  |  |  |  |  |  |  |
| Body mass index | Inverse variance weighted | 530 | 0.35 | 0.17 | 0.04 | 0.71 | - |
| Body mass index | MR Egger | 530 | 1.02 | 0.46 | 0.03 | 0.73 | 0.12 |
| Body mass index | Simple mode | 530 | 0.33 | 0.89 | 0.71 | - | - |
| Body mass index | Weighted median | 530 | 0.33 | 0.29 | 0.25 | - | - |
| Body mass index | Weighted mode | 530 | 0.77 | 0.55 | 0.16 | - | - |
| Waist-hip ratio | Inverse variance weighted | 346 | 0.48 | 0.22 | 0.03 | 0.12 | - |
| Waist-hip ratio | MR Egger | 346 | 1.61 | 0.60 | 0.01 | 0.15 | 0.04 |
| Waist-hip ratio | Simple mode | 346 | -0.75 | 1.09 | 0.49 | - | - |
| Waist-hip ratio | Weighted median | 346 | 0.50 | 0.35 | 0.15 | - | - |
| Waist-hip ratio | Weighted mode | 346 | 0.59 | 0.62 | 0.35 | - | - |
| Lifetime smoking index | Inverse variance weighted | 122 | 0.93 | 0.39 | 0.02 | 0.40 | - |
| Lifetime smoking index | MR Egger | 122 | -1.12 | 1.64 | 0.49 | 0.42 | 0.20 |
| Lifetime smoking index | Simple mode | 122 | -0.81 | 1.58 | 0.61 | - | - |
| Lifetime smoking index | Weighted median | 122 | 0.20 | 0.57 | 0.73 | - | - |
| Lifetime smoking index | Weighted mode | 122 | -0.63 | 1.47 | 0.67 | - | - |
| Smoking initiation | Inverse variance weighted | 192 | 0.59 | 0.23 | 0.01 | 0.57 | - |
| Smoking initiation | MR Egger | 192 | -0.61 | 0.93 | 0.51 | 0.59 | 0.18 |
| Smoking initiation | Simple mode | 192 | -0.09 | 1.03 | 0.93 | - | - |
| Smoking initiation | Weighted median | 192 | 0.38 | 0.34 | 0.26 | - | - |
| Smoking initiation | Weighted mode | 192 | -0.005 | 0.83 | 1.00 | - | - |
| Insomnia | Inverse variance weighted | 154 | 0.29 | 0.12 | 0.01 | 0.50 | - |
| Insomnia | MR Egger | 154 | 0.04 | 0.44 | 0.92 | 0.48 | 0.55 |
| Insomnia | Simple mode | 154 | 0.05 | 0.47 | 0.91 | - | - |
| Insomnia | Weighted median | 154 | 0.18 | 0.18 | 0.32 | - | - |
| Insomnia | Weighted mode | 154 | -0.10 | 0.43 | 0.81 | - | - |
| Problematic alcohol use | Inverse variance weighted | 59 | -0.05 | 0.49 | 0.91 | 0.38 | - |
| Problematic alcohol use | MR Egger | 59 | 0.04 | 1.23 | 0.97 | 0.34 | 0.93 |
| Problematic alcohol use | Simple mode | 59 | -0.47 | 1.41 | 0.74 | - | - |
| Problematic alcohol use | Weighted median | 59 | 0.48 | 0.69 | 0.49 | - | - |
| Problematic alcohol use | Weighted mode | 59 | 0.88 | 0.99 | 0.38 | - | - |
| Drinks per week | Inverse variance weighted | 68 | -0.35 | 0.66 | 0.60 | 0.69 | - |
| Drinks per week | MR Egger | 68 | 0.80 | 1.67 | 0.63 | 0.68 | 0.46 |
| Drinks per week | Simple mode | 68 | 1.75 | 1.99 | 0.38 | - | - |
| Drinks per week | Weighted median | 68 | 1.16 | 0.99 | 0.24 | - | - |
| Drinks per week | Weighted mode | 68 | 1.92 | 1.32 | 0.15 | - | - |
| Fasting insulin | Inverse variance weighted | 33 | 1.20 | 0.70 | 0.08 | 0.90 | - |
| Fasting insulin | MR Egger | 33 | 2.29 | 2.28 | 0.32 | 0.88 | 0.62 |
| Fasting insulin | Simple mode | 33 | 0.57 | 1.71 | 0.74 | - | - |
| Fasting insulin | Weighted median | 33 | 1.02 | 0.97 | 0.29 | - | - |
| Fasting insulin | Weighted mode | 33 | 1.27 | 1.57 | 0.43 | - | - |
| Fasting glucose | Inverse variance weighted | 55 | -0.17 | 0.38 | 0.66 | 0.28 | - |
| Fasting glucose | MR Egger | 55 | -0.95 | 0.66 | 0.16 | 0.32 | 0.16 |
| Fasting glucose | Simple mode | 55 | 0.05 | 1.01 | 0.96 | - | - |
| Fasting glucose | Weighted median | 55 | -0.31 | 0.54 | 0.57 | - | - |
| Fasting glucose | Weighted mode | 55 | -0.21 | 0.50 | 0.68 | - | - |
| Leisure television watching | Inverse variance weighted | 85 | -0.17 | 0.41 | 0.67 | 0.52 | - |
| Leisure television watching | MR Egger | 85 | -3.18 | 1.98 | 0.11 | 0.56 | 0.12 |
| Leisure television watching | Simple mode | 85 | -1.58 | 1.59 | 0.32 | - | - |
| Leisure television watching | Weighted median | 85 | -0.44 | 0.58 | 0.45 | - | - |
| Leisure television watching | Weighted mode | 85 | -1.84 | 1.56 | 0.24 | - | - |
| Relative fat intake | Inverse variance weighted | 7 | 0.96 | 26.78 | 0.97 | 0.03 | - |
| Relative fat intake | MR Egger | 7 | -79.92 | 104.60 | 0.48 | 0.03 | 0.46 |
| Relative fat intake | Simple mode | 7 | -37.17 | 36.48 | 0.35 | - | - |
| Relative fat intake | Weighted median | 7 | -23.71 | 25.73 | 0.36 | - | - |
| Relative fat intake | Weighted mode | 7 | -37.17 | 27.02 | 0.22 | - | - |
| Relative protein intake | Inverse variance weighted | 7 | -15.53 | 28.68 | 0.59 | 0.15 | - |
| Relative protein intake | MR Egger | 7 | 80.98 | 56.64 | 0.21 | 0.35 | 0.12 |
| Relative protein intake | Simple mode | 7 | -56.98 | 54.32 | 0.33 | - | - |
| Relative protein intake | Weighted median | 7 | 3.27 | 33.13 | 0.92 | - | - |
| Relative protein intake | Weighted mode | 7 | 13.20 | 30.53 | 0.68 | - | - |
| Sweet beverage consumption | Inverse variance weighted | 3 | -2.10 | 2.99 | 0.48 | 0.96 | - |
| Sweet beverage consumption | MR Egger | 3 | -2.05 | 30.08 | 0.96 | 0.78 | 1.00 |
| Sweet beverage consumption | Simple mode | 3 | -2.64 | 3.92 | 0.57 | - | - |
| Sweet beverage consumption | Weighted median | 3 | -2.44 | 3.29 | 0.46 | - | - |
| Sweet beverage consumption | Weighted mode | 3 | -2.58 | 4.08 | 0.59 | - | - |
| Two-hour glucose | Inverse variance weighted | 12 | 0.70 | 0.26 | 0.01 | 0.69 | - |
| Two-hour glucose | MR Egger | 12 | 1.08 | 0.67 | 0.14 | 0.64 | 0.55 |
| Two-hour glucose | Simple mode | 12 | 0.32 | 0.64 | 0.62 | - | - |
| Two-hour glucose | Weighted median | 12 | 0.33 | 0.36 | 0.36 | - | - |
| Two-hour glucose | Weighted mode | 12 | 0.20 | 0.51 | 0.71 | - | - |
| Type 2 diabetes | Inverse variance weighted | 38 | 0.07 | 0.14 | 0.64 | 3.30E-05 | - |
| Type 2 diabetes | MR Egger | 38 | 0.12 | 0.38 | 0.75 | 2.17E-05 | 0.87 |
| Type 2 diabetes | Simple mode | 38 | 0.15 | 0.29 | 0.61 | - | - |
| Type 2 diabetes | Weighted median | 38 | 0.15 | 0.15 | 0.30 | - | - |
| Type 2 diabetes | Weighted mode | 38 | 0.17 | 0.19 | 0.39 | - | - |
| Glycated hemoglobin | Inverse variance weighted | 73 | -0.28 | 0.51 | 0.59 | 0.60 | - |
| Glycated hemoglobin | MR Egger | 73 | -0.99 | 0.99 | 0.32 | 0.59 | 0.40 |
| Glycated hemoglobin | Simple mode | 73 | -0.69 | 1.53 | 0.65 | - | - |
| Glycated hemoglobin | Weighted median | 73 | -0.72 | 0.81 | 0.37 | - | - |
| Glycated hemoglobin | Weighted mode | 73 | -0.61 | 0.83 | 0.47 | - | - |
| Body fat percentage | Inverse variance weighted | 10 | 0.51 | 0.55 | 0.36 | 0.49 | - |
| Body fat percentage | MR Egger | 10 | 4.61 | 2.51 | 0.10 | 0.69 | 0.13 |
| Body fat percentage | Simple mode | 10 | 0.10 | 1.22 | 0.94 | - | - |
| Body fat percentage | Weighted median | 10 | 0.50 | 0.75 | 0.50 | - | - |
| Body fat percentage | Weighted mode | 10 | 0.35 | 1.05 | 0.74 | - | - |
| Age of initiation of smoking | Inverse variance weighted | 9 | -0.83 | 1.35 | 0.54 | 0.30 | - |
| Age of initiation of smoking | MR Egger | 9 | 2.84 | 7.18 | 0.70 | 0.24 | 0.62 |
| Age of initiation of smoking | Simple mode | 9 | 1.80 | 2.92 | 0.55 | - | - |
| Age of initiation of smoking | Weighted median | 9 | 1.16 | 1.67 | 0.49 | - | - |
| Age of initiation of smoking | Weighted mode | 9 | 1.70 | 2.48 | 0.51 | - | - |
| Bowls of cereal per week | Inverse variance weighted | 12 | 1.36 | 1.17 | 0.24 | 0.91 | - |
| Bowls of cereal per week | MR Egger | 12 | 5.10 | 5.67 | 0.39 | 0.90 | 0.52 |
| Bowls of cereal per week | Simple mode | 12 | 1.52 | 2.62 | 0.57 | - | - |
| Bowls of cereal per week | Weighted median | 12 | 1.54 | 1.55 | 0.32 | - | - |
| Bowls of cereal per week | Weighted mode | 12 | 2.50 | 2.34 | 0.31 | - | - |
| Cigarettes per day | Inverse variance weighted | 38 | 0.28 | 0.46 | 0.55 | 0.06 | - |
| Cigarettes per day | MR Egger | 38 | -0.62 | 0.80 | 0.44 | 0.07 | 0.18 |
| Cigarettes per day | Simple mode | 38 | 1.78 | 1.47 | 0.23 | - | - |
| Cigarettes per day | Weighted median | 38 | -0.002 | 0.59 | 1.00 | - | - |
| Cigarettes per day | Weighted mode | 38 | 0.24 | 0.54 | 0.65 | - | - |
| Coffee consumption | Inverse variance weighted | 28 | 0.81 | 0.76 | 0.28 | 0.80 | - |
| Coffee consumption | MR Egger | 28 | 0.35 | 1.41 | 0.81 | 0.77 | 0.70 |
| Coffee consumption | Simple mode | 28 | 1.67 | 1.96 | 0.40 | - | - |
| Coffee consumption | Weighted median | 28 | 0.72 | 1.03 | 0.49 | - | - |
| Coffee consumption | Weighted mode | 28 | 0.79 | 0.93 | 0.40 | - | - |
| Glasses of water per day | Inverse variance weighted | 18 | 0.76 | 0.99 | 0.44 | 0.39 | - |
| Glasses of water per day | MR Egger | 18 | 9.76 | 6.21 | 0.14 | 0.46 | 0.16 |
| Glasses of water per day | Simple mode | 18 | 2.85 | 2.65 | 0.30 | - | - |
| Glasses of water per day | Weighted median | 18 | 1.85 | 1.41 | 0.19 | - | - |
| Glasses of water per day | Weighted mode | 18 | 3.13 | 2.74 | 0.27 | - | - |
| Leisure computer use | Inverse variance weighted | 20 | -1.82 | 0.86 | 0.04 | 0.50 | - |
| Leisure computer use | MR Egger | 20 | 2.58 | 6.67 | 0.70 | 0.46 | 0.51 |
| Leisure computer use | Simple mode | 20 | -1.37 | 2.10 | 0.52 | - | - |
| Leisure computer use | Weighted median | 20 | -1.40 | 1.18 | 0.23 | - | - |
| Leisure computer use | Weighted mode | 20 | -1.26 | 2.11 | 0.56 | - | - |
| Glasses of milk intake per week | Wald ratio | 1 | 0.02 | 0.10 | 0.81 | - | - |
| Overall beef intake | Inverse variance weighted | 2 | -0.66 | 2.42 | 0.78 | 0.47 | - |
| Overall cheese intake | Inverse variance weighted | 21 | 0.52 | 0.93 | 0.58 | 0.41 | - |
| Overall cheese intake | MR Egger | 21 | 5.36 | 3.74 | 0.17 | 0.46 | 0.20 |
| Overall cheese intake | Simple mode | 21 | 0.26 | 2.40 | 0.92 | - | - |
| Overall cheese intake | Weighted median | 21 | 0.68 | 1.29 | 0.60 | - | - |
| Overall cheese intake | Weighted mode | 21 | 1.43 | 1.86 | 0.45 | - | - |
| Overall lamb/mutton intake | Inverse variance weighted | 8 | -0.53 | 1.70 | 0.76 | 0.27 | - |
| Overall lamb/mutton intake | MR Egger | 8 | 10.49 | 10.26 | 0.35 | 0.29 | 0.32 |
| Overall lamb/mutton intake | Simple mode | 8 | -4.38 | 4.04 | 0.31 | - | - |
| Overall lamb/mutton intake | Weighted median | 8 | -0.78 | 2.18 | 0.72 | - | - |
| Overall lamb/mutton intake | Weighted mode | 8 | 3.55 | 3.56 | 0.35 | - | - |
| Overall non-oily fish intake | Inverse variance weighted | 2 | 1.83 | 3.22 | 0.57 | 0.87 | - |
| Overall non-oily fish intake | MR Egger | - | - | - | - | - | - |
| Overall oily fish intake | Inverse variance weighted | 21 | -0.43 | 0.87 | 0.62 | 0.62 | - |
| Overall oily fish intake | MR Egger | 21 | -3.93 | 3.41 | 0.26 | 0.63 | 0.30 |
| Overall oily fish intake | Simple mode | 21 | -1.68 | 2.29 | 0.47 | - | - |
| Overall oily fish intake | Weighted median | 21 | -1.33 | 1.22 | 0.28 | - | - |
| Overall oily fish intake | Weighted mode | 21 | -2.09 | 1.84 | 0.27 | - | - |
| Overall pork intake | Inverse variance weighted | 5 | 3.08 | 1.71 | 0.07 | 0.68 | - |
| Overall pork intake | MR Egger | 5 | 4.88 | 4.06 | 0.31 | 0.56 | 0.66 |
| Overall pork intake | Simple mode | 5 | 5.02 | 2.89 | 0.16 | - | - |
| Overall pork intake | Weighted median | 5 | 4.33 | 2.17 | 0.05 | - | - |
| Overall pork intake | Weighted mode | 5 | 4.96 | 2.99 | 0.17 | - | - |
| Overall poultry intake | Inverse variance weighted | 3 | -1.92 | 2.67 | 0.47 | 0.47 | - |
| Overall poultry intake | MR Egger | 3 | -44.06 | 43.49 | 0.50 | 0.45 | 0.51 |
| Overall poultry intake | Simple mode | 3 | -3.86 | 4.08 | 0.44 | - | - |
| Overall poultry intake | Weighted median | 3 | -3.00 | 3.35 | 0.37 | - | - |
| Overall poultry intake | Weighted mode | 3 | -4.16 | 4.40 | 0.44 | - | - |
| Overall processed meat intake | Inverse variance weighted | 6 | -2.25 | 1.88 | 0.23 | 0.40 | - |
| Overall processed meat intake | MR Egger | 6 | -5.73 | 12.97 | 0.68 | 0.28 | 0.80 |
| Overall processed meat intake | Simple mode | 6 | -0.20 | 4.01 | 0.96 | - | - |
| Overall processed meat intake | Weighted median | 6 | -1.54 | 2.35 | 0.51 | - | - |
| Overall processed meat intake | Weighted mode | 6 | -0.09 | 3.88 | 0.98 | - | - |
| Pieces of dried fruit per day | Inverse variance weighted | 10 | 1.16 | 1.69 | 0.49 | 0.17 | - |
| Pieces of dried fruit per day | MR Egger | 10 | 7.48 | 11.23 | 0.52 | 0.14 | 0.58 |
| Pieces of dried fruit per day | Simple mode | 10 | -2.46 | 3.47 | 0.50 | - | - |
| Pieces of dried fruit per day | Weighted median | 10 | 1.15 | 2.03 | 0.57 | - | - |
| Pieces of dried fruit per day | Weighted mode | 10 | -0.85 | 3.42 | 0.81 | - | - |
| Pieces of fresh fruit per day | Inverse variance weighted | 37 | -0.27 | 0.70 | 0.70 | 0.10 | - |
| Pieces of fresh fruit per day | MR Egger | 37 | -1.94 | 2.20 | 0.38 | 0.10 | 0.43 |
| Pieces of fresh fruit per day | Simple mode | 37 | 0.38 | 1.78 | 0.83 | - | - |
| Pieces of fresh fruit per day | Weighted median | 37 | -0.84 | 0.88 | 0.34 | - | - |
| Pieces of fresh fruit per day | Weighted mode | 37 | -1.31 | 1.51 | 0.39 | - | - |
| Processed meat consumption | Inverse variance weighted | 4 | -1.85 | 2.04 | 0.36 | 0.84 | - |
| Processed meat consumption | MR Egger | 4 | 6.48 | 10.90 | 0.61 | 0.88 | 0.52 |
| Processed meat consumption | Simple mode | 4 | -0.30 | 3.39 | 0.93 | - | - |
| Processed meat consumption | Weighted median | 4 | -1.43 | 2.32 | 0.54 | - | - |
| Processed meat consumption | Weighted mode | 4 | -0.30 | 3.27 | 0.93 | - | - |
| Proinsulin | Inverse variance weighted | 24 | 0.003 | 0.14 | 0.98 | 0.46 | - |
| Proinsulin | MR Egger | 24 | 0.004 | 0.24 | 0.99 | 0.40 | 1.00 |
| Proinsulin | Simple mode | 24 | -0.34 | 0.36 | 0.36 | - | - |
| Proinsulin | Weighted median | 24 | -0.03 | 0.20 | 0.88 | - | - |
| Proinsulin | Weighted mode | 24 | -0.16 | 0.22 | 0.47 | - | - |
| Relative carbohydrate intake | Inverse variance weighted | 5 | 14.31 | 18.03 | 0.43 | 0.22 | - |
| Relative carbohydrate intake | MR Egger | 5 | 268.63 | 107.02 | 0.09 | 1.00 | 0.10 |
| Relative carbohydrate intake | Simple mode | 5 | -14.71 | 33.37 | 0.68 | - | - |
| Relative carbohydrate intake | Weighted median | 5 | 11.18 | 19.49 | 0.57 | - | - |
| Relative carbohydrate intake | Weighted mode | 5 | 38.59 | 32.09 | 0.30 | - | - |
| Sleep duration | Inverse variance weighted | 57 | 0.005 | 0.01 | 0.65 | 0.08 | - |
| Sleep duration | MR Egger | 57 | 0.02 | 0.04 | 0.66 | 0.07 | 0.73 |
| Sleep duration | Simple mode | 57 | 0.03 | 0.03 | 0.41 | - | - |
| Sleep duration | Weighted median | 57 | 0.01 | 0.01 | 0.31 | - | - |
| Sleep duration | Weighted mode | 57 | 0.02 | 0.02 | 0.40 | - | - |
| Slices of bread per week | Inverse variance weighted | 10 | 0.76 | 1.20 | 0.53 | 0.72 | - |
| Slices of bread per week | MR Egger | 10 | -4.19 | 7.86 | 0.61 | 0.67 | 0.54 |
| Slices of bread per week | Simple mode | 10 | -0.35 | 2.45 | 0.89 | - | - |
| Slices of bread per week | Weighted median | 10 | -0.31 | 1.51 | 0.84 | - | - |
| Slices of bread per week | Weighted mode | 10 | -0.44 | 2.33 | 0.85 | - | - |
| Smoking cessation | Inverse variance weighted | 12 | -1.07 | 0.57 | 0.06 | 0.40 | - |
| Smoking cessation | MR Egger | 12 | -2.04 | 1.80 | 0.29 | 0.34 | 0.58 |
| Smoking cessation | Simple mode | 12 | -1.41 | 1.04 | 0.20 | - | - |
| Smoking cessation | Weighted median | 12 | -1.12 | 0.75 | 0.13 | - | - |
| Smoking cessation | Weighted mode | 12 | -1.29 | 0.78 | 0.13 | - | - |
| Spent driving | Inverse variance weighted | 4 | 1.97 | 2.51 | 0.43 | 0.18 | - |
| Spent driving | MR Egger | 4 | -20.98 | 17.93 | 0.36 | 0.26 | 0.33 |
| Spent driving | Simple mode | 4 | 0.62 | 3.88 | 0.88 | - | - |
| Spent driving | Weighted median | 4 | 1.26 | 2.54 | 0.62 | - | - |
| Spent driving | Weighted mode | 4 | -0.05 | 3.69 | 0.99 | - | - |
| Tablespoons of cooked vegetables per day | Inverse variance weighted | 7 | -1.19 | 1.70 | 0.48 | 0.78 | - |
| Tablespoons of cooked vegetables per day | MR Egger | 7 | 1.75 | 6.98 | 0.81 | 0.69 | 0.68 |
| Tablespoons of cooked vegetables per day | Simple mode | 7 | 0.44 | 3.13 | 0.89 | - | - |
| Tablespoons of cooked vegetables per day | Weighted median | 7 | 0.36 | 2.10 | 0.86 | - | - |
| Tablespoons of cooked vegetables per day | Weighted mode | 7 | 0.52 | 2.82 | 0.86 | - | - |
| Tablespoons of raw vegetables per day | Inverse variance weighted | 8 | -0.89 | 1.40 | 0.52 | 0.52 | - |
| Tablespoons of raw vegetables per day | MR Egger | 8 | -1.35 | 7.21 | 0.86 | 0.41 | 0.95 |
| Tablespoons of raw vegetables per day | Simple mode | 8 | -1.55 | 2.49 | 0.55 | - | - |
| Tablespoons of raw vegetables per day | Weighted median | 8 | -0.30 | 1.74 | 0.86 | - | - |
| Tablespoons of raw vegetables per day | Weighted mode | 8 | 0.44 | 2.35 | 0.86 | - | - |
| Tea consumption | Inverse variance weighted | 11 | 0.34 | 1.45 | 0.82 | 0.36 | - |
| Tea consumption | MR Egger | 11 | 1.54 | 4.42 | 0.74 | 0.29 | 0.78 |
| Tea consumption | Simple mode | 11 | -0.85 | 2.74 | 0.76 | - | - |
| Tea consumption | Weighted median | 11 | 0.49 | 1.79 | 0.78 | - | - |
| Tea consumption | Weighted mode | 11 | 0.13 | 1.98 | 0.95 | - | - |
| Waist circumference | Inverse variance weighted | 39 | 0.11 | 0.78 | 0.88 | 0.56 | - |
| Waist circumference | MR Egger | 39 | 3.05 | 2.35 | 0.20 | 0.60 | 0.19 |
| Waist circumference | Simple mode | 39 | -1.43 | 2.24 | 0.53 | - | - |
| Waist circumference | Weighted median | 39 | 1.18 | 1.19 | 0.32 | - | - |
| Waist circumference | Weighted mode | 39 | 0.67 | 1.48 | 0.65 | - | - |

**Supplementary Table 12. Results of Mendelian Randomization analysis from main analysis and alternative methods between modifiable risk factors and identified proteins.**

Table can be obtained in OSF data respiratory: https://osf.io/t6byn/files/osfstorage.

**Supplementary Table 13. Associations of body mass index and waist-hip ratio with CLDs after adjusting for proteins and proportion mediated.**

| Exposure | Adjustment | Outcome | Association after adjusting for intermediate | | Proportion mediated (%) |
| --- | --- | --- | --- | --- | --- |
|  |  |  | OR (95% CI) | *P* |  |
| BMI | None | MASLD | 1.69 (1.51,1.88) | 4.33E-21 | - |
| BMI | ACY1 | MASLD | 1.61 (1.43,1.82) | 1.21E-14 | 8.24 |
| BMI | ADH1A | MASLD | 1.58 (1.40,1.79) | 4.85E-13 | 12.04 |
| BMI | AKR7A3 | MASLD | 1.50 (1.31,1.71) | 1.82E-09 | 22.21 |
| BMI | DCXR | MASLD | 1.65 (1.47,1.84) | 6.09E-18 | 4.53 |
| BMI | F9 | MASLD | 1.68 (1.49,1.89) | 6.82E-17 | 0.81 |
| BMI | FBLN1 | MASLD | 1.68 (1.50,1.88) | 6.08E-19 | 0.70 |
| BMI | FTCD | MASLD | 1.64 (1.46,1.84) | 2.80E-17 | 5.19 |
| BMI | NAB1 | MASLD | 1.66 (1.48,1.86) | 1.11E-17 | 3.32 |
| BMI | NCAN | MASLD | 1.67 (1.49,1.88) | 3.18E-18 | 1.25 |
| WHR | None | MASLD | 1.81 (1.57,2.10) | 6.96E-16 | - |
| WHR | A1BG | MASLD | 1.73 (1.48,2.02) | 2.97E-12 | 7.99 |
| WHR | ACY1 | MASLD | 1.38 (1.16,1.66) | 3.92E-04 | 45.43 |
| WHR | ADH1A | MASLD | 1.48 (1.24,1.77) | 1.55E-05 | 34.12 |
| WHR | AKR7A3 | MASLD | 1.50 (1.27,1.78) | 2.50E-06 | 31.61 |
| WHR | BMP1 | MASLD | 1.67 (1.43,1.96) | 2.27E-10 | 13.67 |
| WHR | C4BPA | MASLD | 1.78 (1.52,2.07) | 2.55E-13 | 3.31 |
| WHR | DCXR | MASLD | 1.57 (1.32,1.88) | 5.93E-07 | 23.82 |
| WHR | F9 | MASLD | 1.65 (1.41,1.94) | 6.13E-10 | 15.69 |
| WHR | FTCD | MASLD | 1.58 (1.32,1.89) | 4.55E-07 | 22.78 |
| WHR | IGDCC4 | MASLD | 1.75 (1.50,2.05) | 2.26E-12 | 5.79 |
| WHR | NAB1 | MASLD | 1.71 (1.46,2.00) | 3.57E-11 | 9.99 |
| WHR | NCAN | MASLD | 1.69 (1.44,1.98) | 8.82E-11 | 11.62 |
| BMI | None | Cirrhosis | 1.50 (1.28,1.75) | 3.28E-07 | - |
| BMI | ADH1A | Cirrhosis | 1.44 (1.19,1.74) | 1.38E-04 | 9.05 |
| BMI | DCXR | Cirrhosis | 1.44 (1.21,1.71) | 3.67E-05 | 9.90 |
| WHR | None | Cirrhosis | 1.71 (1.41,2.09) | 9.39E-08 | - |
| WHR | ADH1A | Cirrhosis | 1.65 (1.29,2.10) | 5.13E-05 | 7.44 |
| WHR | DCXR | Cirrhosis | 1.70 (1.35,2.15) | 8.48E-06 | 1.24 |
| Abbreviations: BMI, body mass index; WHR,waist-hip ratio; OR, odds ratio; CI, confidence interval; MASLD, metabolic dysfunction-associated steatotic liver disease; ACY1, Aminoacylase-1; ADH1A, Alcohol dehydrogenase 1A (class I); AKR7A3, Aldo-Keto Reductase Family 7 Member A3; C4BPA, Complement component 4 binding protein alpha; DCXR, Dicarbonyl and L-xylulose reductase; F9, Coagulation Factor IX; FBLN1, Fibulin 1; FTCD, Formimidoyltransferase cyclodeaminase; IGDCC4, Immunoglobulin Superfamily DCC Subclass Member 4; NAB1, NGFI-A binding protein 1; NCAN, Neurocan core protein; A1BG, Alpha-1B-glycoprotein; BMP1, Bone Morphogenetic Protein 1. | | | | | |

**Supplementary Table 14. Baseline characteristics of participants.**

| **Characteristic** | **Overall** |  | **Non-CLD** | **CLD** |  | **Non-CHE** | **CHE** |  | **Training** | **Validation** |
| --- | --- | --- | --- | --- | --- | --- | --- | --- | --- | --- |
|  | **N=34,778** |  | **N=34,102** | **N=676** |  | **N=34,554** | **N=224** |  | **N=24,345** | **N=10,433** |
| Sex |  |  |  |  |  |  |  |  |  |  |
| Female | 18882 (54.3) |  | 18574 (54.5) | 308 (45.6) |  | 18767 (54.3) | 115 (51.3) |  | 13170 (54.1) | 5712 (54.7) |
| Male | 15896 (45.7) |  | 15528 (45.5) | 368 (54.4) |  | 15787 (45.7) | 109 (48.7) |  | 11175 (45.9) | 4721 (45.3) |
| Age, years (mean (SD)) | 57.14 (8.10) |  | 57.13 (8.11) | 57.94 (7.72) |  | 57.12 (8.10) | 61.28 (6.77) |  | 57.18 (8.11) | 57.05 (8.09) |
| TDI, (mean (SD)) | -1.38 (3.05) |  | -1.40 (3.04) | -0.52 (3.31) |  | -1.38 (3.05) | -0.95 (3.21) |  | -1.38 (3.05) | -1.38 (3.05) |
| Smoking status, n (%) |  |  |  |  |  |  |  |  |  |  |
| Never | 18651 (53.6) |  | 18380 (53.9) | 271 (40.1) |  | 18560 (53.7) | 91 (40.6) |  | 13043 (53.6) | 5608 (53.8) |
| Previous | 12489 (35.9) |  | 12203 (35.8) | 286 (42.3) |  | 12387 (35.8) | 102 (45.5) |  | 8777 (36.1) | 3712 (35.6) |
| Current | 3638 (10.5) |  | 3519 (10.3) | 119 (17.6) |  | 3607 (10.4) | 31 (13.8) |  | 2525 (10.4) | 1113 (10.7) |
| Alcohol consumption, n (%) |  |  |  |  |  |  |  |  |  |  |
| Low | 27966 (80.4) |  | 27459 (80.5) | 507 (75.0) |  | 27788 (80.4) | 178 (79.5) |  | 19609 (80.5) | 8357 (80.1) |
| Excess | 5449 (15.7) |  | 5331 (15.6) | 118 (17.5) |  | 5418 (15.7) | 31 (13.8) |  | 3829 (15.7) | 1620 (15.5) |
| Heavy | 1249 (3.6) |  | 1201 (3.5) | 48 (7.1) |  | 1234 (3.6) | 15 (6.7) |  | 831 (3.4) | 418 (4.0) |
| BMI, kg/m^2^ (mean (SD)) | 27.37 (4.73) |  | 27.31 (4.69) | 30.53 (5.70) |  | 27.36 (4.73) | 28.60 (4.99) |  | 27.37 (4.71) | 27.36 (4.79) |
| Physical activity, n (%) |  |  |  |  |  |  |  |  |  |  |
| Not regular | 9181 (26.4) |  | 8953 (26.3) | 228 (33.7) |  | 9095 (26.3) | 86 (38.4) |  | 6440 (26.5) | 2741 (26.3) |
| Regular | 25597 (73.6) |  | 25149 (73.7) | 448 (66.3) |  | 25459 (73.7) | 138 (61.6) |  | 17905 (73.5) | 7692 (73.7) |
| Diet, n (%) |  |  |  |  |  |  |  |  |  |  |
| Unhealthy | 10688 (30.7) |  | 10422 (30.6) | 266 (39.3) |  | 10602 (30.7) | 86 (38.4) |  | 7477 (30.7) | 3211 (30.8) |
| Healthy | 24090 (69.3) |  | 23680 (69.4) | 410 (60.7) |  | 23952 (69.3) | 138 (61.6) |  | 16868 (69.3) | 7222 (69.2) |
| WC, cm (mean (SD)) | 90.21 (13.41) |  | 90.02 (13.32) | 99.66 (14.33) |  | 90.18 (13.40) | 94.48 (13.74) |  | 90.23 (13.37) | 90.18 (13.50) |
| diabetes, n (%) | 1632 (4.7) |  | 1533 (4.5) | 99 (14.6) |  | 1610 (4.7) | 22 (9.8) |  | 1160 (4.8) | 472 (4.5) |
| Hyperlipidemia, n (%) | 1212 (3.5) |  | 1154 (3.4) | 58 (8.6) |  | 1192 (3.4) | 20 (8.9) |  | 860 (3.5) | 352 (3.4) |
| Hypertension, n (%) | 9688 (27.9) |  | 9392 (27.5) | 296 (43.8) |  | 9585 (27.7) | 103 (46.0) |  | 6728 (27.6) | 2960 (28.4) |
| Cholesterol, mmol/L | 5.69 (1.16) |  | 5.70 (1.15) | 5.44 (1.24) |  | 5.69 (1.16) | 5.43 (1.12) |  | 5.68 (1.16) | 5.71 (1.15) |
| Glucose, mmol/L | 5.13 (1.21) |  | 5.12 (1.18) | 5.64 (2.21) |  | 5.13 (1.21) | 5.33 (1.40) |  | 5.12 (1.19) | 5.14 (1.27) |
| Platelets, 10^9 cells/L | 253.02 (59.12) |  | 253.09 (58.85) | 249.40 (71.38) |  | 253.05 (59.05) | 247.92 (69.26) |  | 252.68 (59.15) | 253.81 (59.05) |
| ALT, U/L | 23.28 (13.83) |  | 23.05 (13.33) | 35.15 (27.14) |  | 23.28 (13.83) | 23.88 (14.00) |  | 23.35 (14.22) | 23.11 (12.87) |
| AST, U/L | 26.23 (10.57) |  | 26.05 (10.03) | 35.51 (24.43) |  | 26.21 (10.51) | 28.91 (17.61) |  | 26.29 (11.14) | 26.09 (9.11) |
| GGT, U/L | 36.84 (39.59) |  | 35.99 (37.05) | 80.51 (98.27) |  | 36.73 (39.22) | 54.28 (76.45) |  | 36.72 (38.63) | 37.12 (41.76) |
| Albumin, g/L | 45.19 (2.62) |  | 45.20 (2.61) | 44.82 (2.83) |  | 45.20 (2.61) | 44.48 (2.76) |  | 45.19 (2.62) | 45.20 (2.60) |
| ALP, U/L | 83.54 (25.29) |  | 83.29 (24.86) | 95.92 (39.71) |  | 83.48 (25.25) | 92.50 (29.09) |  | 83.48 (24.74) | 83.66 (26.53) |
| Direct bilirubin, umol/L | 1.84 (0.80) |  | 1.83 (0.80) | 2.08 (1.04) |  | 1.84 (0.80) | 2.02 (0.99) |  | 1.84 (0.80) | 1.84 (0.81) |
| Creatinine, umol/L | 72.18 (15.43) |  | 72.15 (15.36) | 73.75 (18.67) |  | 72.14 (15.30) | 78.38 (28.49) |  | 72.28 (15.48) | 71.97 (15.33) |
| Total bilirubin, umol/L | 9.14 (4.37) |  | 9.13 (4.36) | 9.56 (4.76) |  | 9.14 (4.37) | 9.41 (4.26) |  | 9.14 (4.37) | 9.14 (4.37) |
| Total protein, g/L | 72.35 (4.04) |  | 72.33 (4.02) | 72.96 (4.53) |  | 72.35 (4.03) | 72.04 (4.54) |  | 72.35 (4.04) | 72.33 (4.03) |
| A1BG* | -0.01 (0.19) |  | -0.01 (0.19) | 0.03 (0.18) |  | -0.01 (0.19) | 0.03 (0.20) |  | -0.006 (0.19) | -0.01 (0.19) |
| ACY1* | 0.07 (0.76) |  | 0.05 (0.75) | 0.81 (0.97) |  | 0.06 (0.76) | 0.32 (0.88) |  | 0.07 (0.76) | 0.06 (0.76) |
| DCXR* | 0.05 (0.65) |  | 0.04 (0.64) | 0.45 (0.78) |  | 0.04 (0.65) | 0.12 (0.71) |  | 0.05 (0.65) | 0.04 (0.64) |
| ERBB3* | 0.003 (0.23) |  | 0.003 (0.23) | 0.04 (0.25) |  | 0.003 (0.23) | 0.01 (0.26) |  | 0.003 (0.23) | 0.01 (0.23) |
| F9* | 0.002 (0.21) |  | 0.0001 (0.21) | 0.09 (0.22) |  | 0.001 (0.21) | 0.05 (0.23) |  | 0.0003 (0.21) | 0.01 (0.22) |
| FTCD* | 0.13 (0.95) |  | 0.11 (0.94) | 0.93 (1.17) |  | 0.12 (0.95) | 0.37 (1.06) |  | 0.13 (0.95) | 0.12 (0.95) |
| IGDCC4* | -0.01 (0.28) |  | -0.004 (0.28) | -0.08 (0.29) |  | -0.005 (0.28) | -0.09 (0.31) |  | -0.005 (0.28) | -0.01 (0.28) |
| IGSF3* | 0.03 (0.41) |  | 0.03 (0.40) | 0.43 (0.60) |  | 0.03 (0.41) | 0.25 (0.53) |  | 0.04 (0.41) | 0.03 (0.40) |
| NCAN* | -0.02 (0.47) |  | -0.02 (0.47) | -0.19 (0.49) |  | -0.02 (0.47) | -0.10 (0.49) |  | -0.02 (0.47) | -0.02 (0.48) |
| ADH1B* | 0.11 (0.82) |  | 0.10 (0.81) | 0.72 (1.03) |  | 0.11 (0.82) | 0.24 (0.89) |  | 0.11 (0.83) | 0.11 (0.81) |
| Data are n (%) or mean (SD). *Protein data are processed by UK Biobank, not actual plasma levels. Abbreviations: CLD, chronic liver disease; CHE, composite hepatic events; TDI, Townsend deprivation index; BMI, body mass index; WC, waist circumference; ALT, Alanine aminotransferase; AST, aspartate aminotransferase; GGT, gamma-glutamyltransferase; ALP, alkaline phosphatase; A1BG, Alpha-1B-glycoprotein; ACY1, Aminoacylase-1; DCXR, Dicarbonyl and L-xylulose reductase; ERBB3, Erb-B2 Receptor Tyrosine Kinase 3; F9, Coagulation Factor IX; FTCD, Formimidoyltransferase cyclodeaminase; IGDCC4, Immunoglobulin Superfamily DCC Subclass Member 4; IGSF3, Immunoglobulin superfamily member 3; NCAN, Neurocan core protein; ADH1B, Alcohol dehydrogenase 1B. | | | | | | | | | | |

**Supplementary Table 15. Associations between 10 proteins and liver diseases.**

| **Protein** | **Disease** | **Primary model** | |  | **Full adjusted model** | |
| --- | --- | --- | --- | --- | --- | --- |
|  |  | **HR (95% CI)** | ***P*** |  | **HR (95% CI)** | ***P*** |
| A1BG | Chronic liver disease | 2.84 (1.86, 4.34) | 1.21E-06 |  | 1.96 (1.23, 3.11) | 4.45E-03 |
| ACY1 | Chronic liver disease | 2.73 (2.52, 2.96) | 5.63E-130 |  | 2.28 (2.07, 2.51) | 7.70E-63 |
| DCXR | Chronic liver disease | 2.24 (2.03, 2.48) | 1.03E-54 |  | 1.82 (1.62, 2.03) | 6.85E-25 |
| ERBB3 | Chronic liver disease | 1.80 (1.29, 2.51) | 5.61E-04 |  | 1.77 (1.25, 2.51) | 1.27E-03 |
| F9 | Chronic liver disease | 5.64 (4.05, 7.84) | 1.08E-24 |  | 1.54 (1.03, 2.31) | 0.04 |
| FTCD | Chronic liver disease | 2.00 (1.87, 2.13) | 7.44E-98 |  | 1.68 (1.56, 1.81) | 1.44E-43 |
| IGDCC4 | Chronic liver disease | 0.42 (0.33, 0.54) | 4.64E-12 |  | 0.63 (0.48, 0.84) | 1.74E-03 |
| IGSF3 | Chronic liver disease | 2.71 (2.51, 2.93) | 9.30E-146 |  | 2.41 (2.17, 2.67) | 2.74E-63 |
| NCAN | Chronic liver disease | 0.47 (0.40, 0.55) | 3.93E-21 |  | 0.93 (0.77, 1.12) | 0.43 |
| ADH1B | Chronic liver disease | 1.95 (1.82, 2.10) | 9.95E-78 |  | 1.64 (1.51, 1.77) | 8.07E-33 |
| A1BG | MASLD | 3.34 (2.04, 5.47) | 1.73E-06 |  | 1.85 (1.08, 3.18) | 0.03 |
| ACY1 | MASLD | 2.82 (2.57, 3.10) | 4.25E-104 |  | 2.41 (2.16, 2.69) | 9.86E-55 |
| DCXR | MASLD | 2.36 (2.10, 2.65) | 2.17E-46 |  | 1.98 (1.74, 2.25) | 1.77E-24 |
| ERBB3 | MASLD | 2.56 (1.75, 3.73) | 1.12E-06 |  | 2.34 (1.58, 3.48) | 2.52E-05 |
| F9 | MASLD | 7.91 (5.49, 11.4) | 1.25E-28 |  | 2.46 (1.55, 3.91) | 1.36E-04 |
| FTCD | MASLD | 2.09 (1.94, 2.25) | 2.58E-83 |  | 1.75 (1.61, 1.91) | 2.23E-38 |
| IGDCC4 | MASLD | 0.40 (0.30, 0.53) | 1.67E-10 |  | 0.60 (0.43, 0.83) | 2.39E-03 |
| IGSF3 | MASLD | 2.69 (2.46, 2.95) | 4.78E-103 |  | 2.27 (2.01, 2.58) | 6.76E-38 |
| NCAN | MASLD | 0.48 (0.40, 0.58) | 5.71E-15 |  | 0.91 (0.74, 1.13) | 0.40 |
| ADH1B | MASLD | 2.02 (1.87, 2.19) | 6.20E-66 |  | 1.68 (1.53, 1.85) | 3.64E-28 |
| A1BG | Alcoholic liver disease | 1.64 (0.44, 6.07) | 0.46 |  | 2.37 (0.62, 9.14) | 0.21 |
| ACY1 | Alcoholic liver disease | 3.63 (2.86, 4.60) | 2.11E-26 |  | 2.86 (2.17, 3.77) | 1.24E-13 |
| DCXR | Alcoholic liver disease | 2.61 (1.93, 3.53) | 4.84E-10 |  | 1.98 (1.43, 2.76) | 4.56E-05 |
| ERBB3 | Alcoholic liver disease | 2.57 (0.94, 7.02) | 0.06 |  | 1.91 (0.65, 5.66) | 0.24 |
| F9 | Alcoholic liver disease | 8.21 (3.13, 21.53) | 1.89E-05 |  | 2.62 (0.77, 8.85) | 0.12 |
| FTCD | Alcoholic liver disease | 2.16 (1.77, 2.63) | 1.53E-14 |  | 1.96 (1.57, 2.44) | 2.80E-09 |
| IGDCC4 | Alcoholic liver disease | 0.34 (0.17, 0.69) | 2.81E-03 |  | 0.43 (0.18, 1.02) | 0.06 |
| IGSF3 | Alcoholic liver disease | 2.74 (2.17, 3.46) | 2.20E-17 |  | 2.79 (2.10, 3.70) | 1.10E-12 |
| NCAN | Alcoholic liver disease | 0.39 (0.24, 0.63) | 1.28E-04 |  | 0.91 (0.51, 1.62) | 0.75 |
| ADH1B | Alcoholic liver disease | 2.04 (1.65, 2.52) | 4.80E-11 |  | 1.85 (1.44, 2.38) | 1.32E-06 |
| A1BG | Cirrhosis | 2.31 (1.09, 4.90) | 0.03 |  | 2.07 (0.91, 4.73) | 0.08 |
| ACY1 | Cirrhosis | 3.25 (2.83, 3.73) | 3.33E-62 |  | 2.60 (2.20, 3.07) | 1.96E-29 |
| DCXR | Cirrhosis | 2.44 (2.05, 2.91) | 3.04E-23 |  | 1.85 (1.52, 2.26) | 1.10E-09 |
| ERBB3 | Cirrhosis | 0.70 (0.38, 1.31) | 0.27 |  | 0.85 (0.44, 1.62) | 0.62 |
| F9 | Cirrhosis | 1.93 (1.01, 3.67) | 0.05 |  | 0.32 (0.16, 0.64) | 1.32E-03 |
| FTCD | Cirrhosis | 2.18 (1.95, 2.44) | 2.32E-42 |  | 1.80 (1.59, 2.05) | 1.87E-19 |
| IGDCC4 | Cirrhosis | 0.42 (0.27, 0.64) | 8.26E-05 |  | 0.72 (0.44, 1.20) | 0.21 |
| IGSF3 | Cirrhosis | 3.16 (2.84, 3.51) | 7.49E-101 |  | 3.08 (2.69, 3.53) | 3.12E-59 |
| NCAN | Cirrhosis | 0.45 (0.34, 0.60) | 1.90E-08 |  | 1.09 (0.79, 1.51) | 0.60 |
| ADH1B | Cirrhosis | 2.13 (1.89, 2.40) | 5.73E-36 |  | 1.80 (1.57, 2.07) | 6.82E-17 |
| A1BG | Liver cancer | 0.90 (0.07, 12.06) | 0.93 |  | 0.60 (0.03, 11.32) | 0.74 |
| ACY1 | Liver cancer | 3.50 (2.16, 5.65) | 3.12E-07 |  | 2.31 (1.30, 4.12) | 4.48E-03 |
| DCXR | Liver cancer | 3.14 (1.77, 5.57) | 9.63E-05 |  | 2.17 (1.12, 4.20) | 0.02 |
| ERBB3 | Liver cancer | 0.52 (0.06, 4.72) | 0.56 |  | 1.36 (0.14, 13.05) | 0.79 |
| F9 | Liver cancer | 4.95 (0.60, 41.06) | 0.14 |  | 0.64 (0.05, 8.22) | 0.73 |
| FTCD | Liver cancer | 2.40 (1.63, 3.53) | 8.87E-06 |  | 1.71 (1.10, 2.65) | 0.02 |
| IGDCC4 | Liver cancer | 0.20 (0.09, 0.47) | 2.31E-04 |  | 0.26 (0.07, 0.97) | 0.05 |
| IGSF3 | Liver cancer | 3.22 (2.24, 4.63) | 2.43E-10 |  | 2.89 (1.62, 5.17) | 3.49E-04 |
| NCAN | Liver cancer | 0.25 (0.10, 0.61) | 2.35E-03 |  | 0.88 (0.29, 2.69) | 0.82 |
| ADH1B | Liver cancer | 2.30 (1.55, 3.42) | 3.97E-05 |  | 1.71 (1.06, 2.77) | 0.03 |
| A1BG | Composite hepatic event | 3.09 (1.49, 6.39) | 2.39E-03 |  | 1.80 (0.81, 3.99) | 0.15 |
| ACY1 | Composite hepatic event | 1.41 (1.20, 1.66) | 3.43E-05 |  | 1.23 (1.02, 1.50) | 0.03 |
| DCXR | Composite hepatic event | 1.13 (0.93, 1.39) | 0.23 |  | 0.96 (0.77, 1.21) | 0.76 |
| ERBB3 | Composite hepatic event | 1.14 (0.63, 2.07) | 0.66 |  | 1.29 (0.69, 2.41) | 0.42 |
| F9 | Composite hepatic event | 2.85 (1.55, 5.26) | 7.87E-04 |  | 1.18 (0.58, 2.38) | 0.64 |
| FTCD | Composite hepatic event | 1.24 (1.09, 1.41) | 1.25E-03 |  | 1.09 (0.94, 1.27) | 0.25 |
| IGDCC4 | Composite hepatic event | 0.40 (0.26, 0.60) | 1.14E-05 |  | 0.72 (0.44, 1.19) | 0.20 |
| IGSF3 | Composite hepatic event | 2.07 (1.71, 2.52) | 1.85E-13 |  | 1.75 (1.35, 2.26) | 1.91E-05 |
| NCAN | Composite hepatic event | 0.72 (0.54, 0.95) | 0.02 |  | 0.98 (0.71, 1.35) | 0.90 |
| ADH1B | Composite hepatic event | 1.15 (0.99, 1.35) | 0.07 |  | 0.99 (0.83, 1.17) | 0.88 |
| A1BG | Ascites | 2.63 (0.73, 9.41) | 0.14 |  | 1.87 (0.47, 7.51) | 0.38 |
| ACY1 | Ascites | 1.91 (1.47, 2.49) | 1.53E-06 |  | 1.88 (1.38, 2.58) | 7.07E-05 |
| DCXR | Ascites | 1.62 (1.16, 2.26) | 4.46E-03 |  | 1.53 (1.06, 2.21) | 0.02 |
| ERBB3 | Ascites | 0.40 (0.14, 1.17) | 0.09 |  | 0.42 (0.14, 1.33) | 0.14 |
| F9 | Ascites | 0.85 (0.28, 2.54) | 0.77 |  | 0.33 (0.10, 1.07) | 0.07 |
| FTCD | Ascites | 1.63 (1.32, 2.01) | 4.08E-06 |  | 1.58 (1.25, 1.99) | 1.15E-04 |
| IGDCC4 | Ascites | 0.39 (0.19, 0.81) | 0.01 |  | 0.64 (0.27, 1.50) | 0.30 |
| IGSF3 | Ascites | 2.34 (1.75, 3.12) | 8.33E-09 |  | 2.17 (1.52, 3.10) | 2.04E-05 |
| NCAN | Ascites | 0.57 (0.35, 0.93) | 0.02 |  | 0.76 (0.44, 1.32) | 0.33 |
| ADH1B | Ascites | 1.43 (1.12, 1.84) | 4.67E-03 |  | 1.34 (1.02, 1.77) | 0.03 |
| A1BG | Variceal bleeding | 0.71 (0.01, 272.04) | 0.91 |  | 3.06 (0, 3364.64) | 0.75 |
| ACY1 | Variceal bleeding | 4.57 (1.57, 13.35) | 5.44E-03 |  | 4.36 (1.58, 12.02) | 4.44E-03 |
| DCXR | Variceal bleeding | 6.12 (2.20, 17.03) | 5.26E-04 |  | 7.76 (2.29, 26.23) | 9.85E-04 |
| ERBB3 | Variceal bleeding | 0.05 (0.01, 5.25) | 0.20 |  | 0.75 (0.01, 129.24) | 0.91 |
| F9 | Variceal bleeding | 0.11 (0.01, 8.98) | 0.33 |  | 0.04 (0.01, 2.15) | 0.11 |
| FTCD | Variceal bleeding | 3.30 (1.39, 7.80) | 6.61E-03 |  | 3.5 0(1.45, 8.42) | 5.21E-03 |
| IGDCC4 | Variceal bleeding | 0.17 (0.04, 0.79) | 0.02 |  | 0.10 (0.01, 0.70) | 0.02 |
| IGSF3 | Variceal bleeding | 3.20 (1.37, 7.46) | 6.99E-03 |  | 2.83 (0.94, 8.51) | 0.06 |
| NCAN | Variceal bleeding | 0.32 (0.04, 2.69) | 0.29 |  | 0.92 (0.09, 9.69) | 0.95 |
| ADH1B | Variceal bleeding | 3.04 (1.42, 6.50) | 4.15E-03 |  | 3.03 (1.33, 6.90) | 8.40E-03 |
| A1BG | Spontaneous bacterial peritonitis | 2.85 (0.79, 10.30) | 0.11 |  | 1.50 (0.37, 6.09) | 0.57 |
| ACY1 | Spontaneous bacterial peritonitis | 1.56 (1.18, 2.07) | 1.99E-03 |  | 1.22 (0.87, 1.70) | 0.24 |
| DCXR | Spontaneous bacterial peritonitis | 1.01 (0.70, 1.46) | 0.94 |  | 0.76 (0.50, 1.14) | 0.19 |
| ERBB3 | Spontaneous bacterial peritonitis | 1.83 (0.66, 5.03) | 0.24 |  | 1.98 (0.69, 5.72) | 0.21 |
| F9 | Spontaneous bacterial peritonitis | 6.88 (2.59, 18.26) | 1.10E-04 |  | 2.57 (0.77, 8.52) | 0.12 |
| FTCD | Spontaneous bacterial peritonitis | 1.17 (0.92, 1.48) | 0.19 |  | 0.93 (0.71, 1.22) | 0.61 |
| IGDCC4 | Spontaneous bacterial peritonitis | 0.34 (0.17, 0.68) | 2.19E-03 |  | 0.63 (0.27, 1.51) | 0.30 |
| IGSF3 | Spontaneous bacterial peritonitis | 2.33 (1.74, 3.12) | 1.15E-08 |  | 1.94 (1.32, 2.87) | 8.33E-04 |
| NCAN | Spontaneous bacterial peritonitis | 0.77 (0.47, 1.27) | 0.30 |  | 1.37 (0.78, 2.43) | 0.27 |
| ADH1B | Spontaneous bacterial peritonitis | 1.12 (0.85, 1.48) | 0.41 |  | 0.88 (0.64, 1.20) | 0.41 |
| A1BG | Hepatic encephalopathy | 3.08 (1.02, 9.28) | 0.05 |  | 1.60 (0.48, 5.35) | 0.45 |
| ACY1 | Hepatic encephalopathy | 1.06 (0.81, 1.38) | 0.67 |  | 0.87 (0.63, 1.19) | 0.38 |
| DCXR | Hepatic encephalopathy | 0.90 (0.65, 1.24) | 0.51 |  | 0.74 (0.51, 1.06) | 0.10 |
| ERBB3 | Hepatic encephalopathy | 1.76 (0.73, 4.22) | 0.21 |  | 2.00 (0.79, 5.02) | 0.14 |
| F9 | Hepatic encephalopathy | 2.89 (1.14, 7.30) | 0.02 |  | 1.19 (0.41, 3.47) | 0.75 |
| FTCD | Hepatic encephalopathy | 1.01 (0.81, 1.25) | 0.93 |  | 0.86 (0.67, 1.10) | 0.22 |
| IGDCC4 | Hepatic encephalopathy | 0.44 (0.23, 0.84) | 0.01 |  | 0.91 (0.42, 1.95) | 0.81 |
| IGSF3 | Hepatic encephalopathy | 1.58 (1.07, 2.33) | 0.02 |  | 1.22 (0.74, 2.01) | 0.44 |
| NCAN | Hepatic encephalopathy | 0.74 (0.48, 1.13) | 0.16 |  | 0.89 (0.54, 1.45) | 0.64 |
| ADH1B | Hepatic encephalopathy | 0.98 (0.76, 1.26) | 0.89 |  | 0.82 (0.62, 1.09) | 0.18 |
| A1BG | Liver failure | 3.31 (0.57, 19.34) | 0.18 |  | 3.15 (0.43, 22.96) | 0.26 |
| ACY1 | Liver failure | 3.74 (2.72, 5.13) | 4.59E-16 |  | 3.09 (2.12, 4.50) | 3.88E-09 |
| DCXR | Liver failure | 3.06 (2.08, 4.50) | 1.39E-08 |  | 2.40 (1.56, 3.70) | 7.30E-05 |
| ERBB3 | Liver failure | 2.18 (0.55, 8.59) | 0.27 |  | 3.08 (0.83, 11.45) | 0.09 |
| F9 | Liver failure | 12.6 (3.96, 40.14) | 1.82E-05 |  | 3.72 (0.73, 18.90) | 0.11 |
| FTCD | Liver failure | 2.21 (1.70, 2.87) | 3.16E-09 |  | 1.82 (1.34, 2.46) | 1.05E-04 |
| IGDCC4 | Liver failure | 0.94 (0.30, 2.97) | 0.92 |  | 2.53 (0.81, 7.92) | 0.11 |
| IGSF3 | Liver failure | 3.13 (2.43, 4.03) | 1.11E-18 |  | 3.08 (2.18, 4.35) | 1.68E-10 |
| NCAN | Liver failure | 0.31 (0.17, 0.57) | 2.01E-04 |  | 0.87 (0.41, 1.82) | 0.71 |
| ADH1B | Liver failure | 2.14 (1.63, 2.83) | 6.64E-08 |  | 1.81 (1.31, 2.51) | 3.23E-04 |
| Abbreviations: HR, hazard ratio; CI, confidence interval; MASLD, metabolic dysfunction-associated steatotic liver disease; A1BG, Alpha-1B-glycoprotein; ACY1, Aminoacylase-1; DCXR, Dicarbonyl and L-xylulose reductase; ERBB3, Erb-B2 Receptor Tyrosine Kinase 3; F9, Coagulation Factor IX; FTCD, Formimidoyltransferase cyclodeaminase; IGDCC4, Immunoglobulin Superfamily DCC Subclass Member 4; IGSF3, Immunoglobulin superfamily member 3; NCAN, Neurocan core protein; ADH1B, Alcohol dehydrogenase 1B. Fully adjusted model was adjusted for gender, age, Townsend deprivation index, education, smoking status, alcohol consumption, body mass index, physical activity, healthy diet, waist circumference, hyperlipidemia, and hypertension. | | | | | | |

**Supplementary Table 16. Associations between proteins and progression of chronic liver disease.**

| **Protein** | **Crude model** | | |  | **Fully adjusted model** | | |
| --- | --- | --- | --- | --- | --- | --- | --- |
|  | **HR (95% CI)** | ***P*** | ***P* fdr** |  | **HR (95% CI)** | ***P*** | ***P* fdr** |
| **Early-stage (MASLD/ALD) to advanced-stage (cirrhosis/cancer)** | | | | | | | |
| A1BG | 0.75 (0.16, 3.42) | 0.71 | 0.85 |  | 2.06 (0.34, 12.43) | 0.43 | 0.65 |
| ACY1 | 1.88 (1.39, 2.56) | 5.29E-05 | 2.64E-04 |  | 1.83 (1.29, 2.59) | 6.32E-04 | 2.11E-03 |
| DCXR | 1.68 (1.16, 2.42) | 5.29E-05 | 0.01 |  | 1.68 (1.13, 2.49) | 0.01 | 0.02 |
| ERBB3 | 0.90 (0.26, 3.14) | 0.87 | 0.87 |  | 1.09 (0.27, 4.44) | 0.90 | 0.90 |
| F9 | 0.46 (0.10, 2.15) | 0.32 | 0.46 |  | 0.51 (0.09, 2.93) | 0.45 | 0.65 |
| FTCD | 1.39 (1.07, 1.80) | 0.01 | 0.03 |  | 1.54 (1.15, 2.07) | 3.74E-03 | 9.35E-03 |
| IGDCC4 | 1.18 (0.40, 3.45) | 0.77 | 0.85 |  | 1.23 (0.37, 4.09) | 0.73 | 0.81 |
| IGSF3 | 2.71 (1.79, 4.10) | 2.21E-06 | 2.21E-05 |  | 3.43 (2.15, 5.45) | 2.05E-07 | 2.05E-06 |
| NCAN | 0.62 (0.33, 1.17) | 0.14 | 0.23 |  | 0.83 (0.40, 1.71) | 0.60 | 0.76 |
| ADH1B | 1.58 (1.19, 2.10) | 1.55E-03 | 5.18E-03 |  | 1.77 (1.31, 2.39) | 1.84E-04 | 9.21E-04 |
| **Non MASLD/ALD to advanced-stage (cirrhosis/cancer)** | | | | | | | |
| A1BG | 2.85 (1.67, 4.86) | 1.14E-04 | 1.26E-04 |  | 2.03 (1.13, 3.65) | 0.02 | 0.03 |
| ACY1 | 2.12 (1.90, 2.36) | 2.66E-42 | 1.33E-41 |  | 1.76 (1.55, 2.00) | 1.07E-17 | 5.37E-17 |
| DCXR | 1.65 (1.44, 1.90) | 8.93E-13 | 1.79E-12 |  | 1.33 (1.14, 1.55) | 3.49E-04 | 6.99E-04 |
| ERBB3 | 1.01 (0.65, 1.56) | 0.96 | 0.96 |  | 1.15 (0.73, 1.81) | 0.55 | 0.61 |
| F9 | 2.47 (1.57, 3.87) | 8.52E-05 | 1.07E-04 |  | 0.65 (0.39, 1.09) | 0.10 | 0.13 |
| FTCD | 1.65 (1.51, 1.80) | 1.52E-29 | 5.08E-29 |  | 1.40 (1.27, 1.54) | 2.11E-11 | 7.03E-11 |
| IGDCC4 | 0.40 (0.30, 0.55) | 6.48E-09 | 1.08E-08 |  | 0.72 (0.50, 1.03) | 0.07 | 0.10 |
| IGSF3 | 2.66 (2.41, 2.94) | 6.83E-84 | 6.83E-83 |  | 2.44 (2.14, 2.78) | 8.38E-41 | 8.38E-40 |
| NCAN | 0.59 (0.48, 0.72) | 2.52E-07 | 3.60E-07 |  | 1.06 (0.84, 1.33) | 0.65 | 0.65 |
| ADH1B | 1.62 (1.46, 1.78) | 9.56E-22 | 2.39E-21 |  | 1.37 (1.23, 1.53) | 2.74E-08 | 6.85E-08 |
| **Noncirrhosis to liver cancer** | | | | | | | |
| A1BG | 1.34 (0.30, 5.95) | 0.70 | 0.77 |  | 0.78 (0.15, 4.06) | 0.77 | 0.77 |
| ACY1 | 1.68 (1.21, 2.32) | 1.80E-03 | 6.01E-03 |  | 1.73 (1.20, 2.48) | 3.34E-03 | 0.011 |
| DCXR | 1.51 (1.02, 2.24) | 0.04 | 0.07 |  | 1.54 (1.00, 2.38) | 0.05 | 0.08 |
| ERBB3 | 1.11 (0.33, 3.74) | 0.87 | 0.87 |  | 1.83 (0.50, 6.72) | 0.36 | 0.40 |
| F9 | 2.90 (0.82, 10.19) | 0.10 | 0.12 |  | 2.65 (0.68, 10.39) | 0.16 | 0.23 |
| FTCD | 1.46 (1.13, 1.89) | 3.39E-03 | 8.48E-03 |  | 1.44 (1.09, 1.90) | 0.01 | 0.025 |
| IGDCC4 | 0.43 (0.18, 1.05) | 0.06 | 0.09 |  | 0.59 (0.22, 1.59) | 0.30 | 0.37 |
| IGSF3 | 2.34 (1.63, 3.35) | 4.46E-06 | 4.46E-05 |  | 2.05 (1.30, 3.23) | 1.88E-03 | 0.009 |
| NCAN | 0.31 (0.18, 0.52) | 1.14E-05 | 5.69E-05 |  | 0.36 (0.19, 0.66) | 1.05E-03 | 0.009 |
| ADH1B | 1.45 (1.08, 1.94) | 0.01 | 0.03 |  | 1.44 (1.05, 1.97) | 0.02 | 0.046 |
| **Cirrhosis to liver cancer** | | | | | | | |
| A1BG | 1.49 (0.03, 65.52) | 0.84 | 0.84 |  | 8.04 (0.04, 1496.27) | 0.43 | 0.78 |
| ACY1 | 0.84 (0.42, 1.71) | 0.64 | 0.79 |  | 0.73 (0.31, 1.71) | 0.47 | 0.78 |
| DCXR | 0.73 (0.29, 1.82) | 0.50 | 0.79 |  | 0.57 (0.18, 1.80) | 0.34 | 0.78 |
| ERBB3 | 0.47 (0.03, 8.36) | 0.61 | 0.79 |  | 0.39 (0.01, 16.84) | 0.62 | 0.78 |
| F9 | 0.22 (0.01, 4.11) | 0.31 | 0.79 |  | 0.02 (0.01, 0.52) | 0.03 | 0.27 |
| FTCD | 0.81 (0.42, 1.55) | 0.52 | 0.79 |  | 0.82 (0.38, 1.74) | 0.60 | 0.78 |
| IGDCC4 | 1.43 (0.17, 12.23) | 0.74 | 0.83 |  | 1.31 (0.09, 18.62) | 0.84 | 0.94 |
| IGSF3 | 1.55 (0.56, 4.30) | 0.40 | 0.79 |  | 1.35 (0.42, 4.37) | 0.62 | 0.78 |
| NCAN | 0.65 (0.17, 2.41) | 0.52 | 0.79 |  | 0.98 (0.16, 6.16) | 0.99 | 0.99 |
| ADH1B | 0.55 (0.24, 1.24) | 0.15 | 0.79 |  | 0.53 (0.22, 1.23) | 0.14 | 0.70 |
| Abbreviations: HR, hazard ratio; CI, confidence interval; fdr, false discovery rate; MASLD, metabolic dysfunction-associated steatotic liver disease; ALD, alcoholic liver disease; A1BG, Alpha-1B-glycoprotein; ACY1, Aminoacylase-1; DCXR, Dicarbonyl and L-xylulose reductase; ERBB3, Erb-B2 Receptor Tyrosine Kinase 3; F9, Coagulation Factor IX; FTCD, Formimidoyltransferase cyclodeaminase; IGDCC4, Immunoglobulin Superfamily DCC Subclass Member 4; IGSF3, Immunoglobulin superfamily member 3; NCAN, Neurocan core protein; ADH1B, Alcohol dehydrogenase 1B. Fully adjusted model was adjusted for gender, age, Townsend deprivation index, education, smoking status, alcohol consumption, body mass index, physical activity, healthy diet, waist circumference, hyperlipidemia, and hypertension. | | | | | | | |

**Supplementary Table 17. Protein-protein interaction analysis of 10 proteins in chronic liver disease risk.**

| **Protein-protein interaction** | **HR (95% CI)** | ***P*** |
| --- | --- | --- |
| A1BG:ACY1 | 1.03 (0.13, 8.28) | 0.98 |
| A1BG:DCXR | 0.87 (0.09, 8.24) | 0.90 |
| A1BG:ERBB3 | 0.34 (0.01, 9.40) | 0.53 |
| A1BG:F9 | 29.83 (0.34, 2606.49) | 0.14 |
| A1BG:FTCD | 0.69 (0.13, 3.76) | 0.67 |
| A1BG:IGDCC4 | 0.23 (0.01, 4.58) | 0.33 |
| A1BG:IGSF3 | 2.15 (0.41, 11.22) | 0.36 |
| A1BG:NCAN | 0.41 (0.08, 2.19) | 0.30 |
| A1BG:ADH1B | 1.37 (0.20, 9.32) | 0.75 |
| ACY1:DCXR | 0.59 (0.29, 1.20) | 0.14 |
| ACY1:ERBB3 | 1.07 (0.23, 5.01) | 0.93 |
| ACY1:F9 | 0.53 (0.09, 2.97) | 0.47 |
| ACY1:FTCD | 0.90 (0.50, 1.62) | 0.73 |
| ACY1:IGDCC4 | 0.70 (0.20, 2.48) | 0.58 |
| ACY1:IGSF3 | 2.05 (1.13, 3.73) | 0.02 |
| ACY1:NCAN | 1.17 (0.61, 2.21) | 0.64 |
| ACY1:ADH1B | 1.45 (0.75, 2.83) | 0.27 |
| DCXR:ERBB3 | 1.31 (0.23, 7.63) | 0.76 |
| DCXR:F9 | 1.60 (0.24, 10.59) | 0.63 |
| DCXR:FTCD | 1.81 (0.95, 3.45) | 0.07 |
| DCXR:IGDCC4 | 0.49 (0.12, 2.03) | 0.32 |
| DCXR:IGSF3 | 0.54 (0.26, 1.11) | 0.09 |
| DCXR:NCAN | 0.86 (0.42, 1.77) | 0.69 |
| DCXR:ADH1B | 1.36 (0.68, 2.70) | 0.38 |
| ERBB3:F9 | 0.56 (0.02, 16.08) | 0.74 |
| ERBB3:FTCD | 3.31 (0.92, 11.92) | 0.07 |
| ERBB3:IGDCC4 | 0.43 (0.04, 4.76) | 0.49 |
| ERBB3:IGSF3 | 0.35 (0.09, 1.45) | 0.15 |
| ERBB3:NCAN | 0.86 (0.27, 2.71) | 0.80 |
| ERBB3:ADH1B | 0.16 (0.04, 0.71) | 0.02 |
| F9:FTCD | 1.42 (0.35, 5.67) | 0.62 |
| F9:IGDCC4 | 1.08 (0.08, 15.50) | 0.95 |
| F9:IGSF3 | 0.42 (0.10, 1.79) | 0.24 |
| F9:NCAN | 2.73 (0.70, 10.58) | 0.15 |
| F9:ADH1B | 0.55 (0.10, 2.97) | 0.49 |
| FTCD:IGDCC4 | 2.53 (0.94, 6.81) | 0.07 |
| FTCD:IGSF3 | 0.75 (0.41, 1.37) | 0.35 |
| FTCD:NCAN | 1.01 (0.59, 1.74) | 0.97 |
| FTCD:ADH1B | 0.66 (0.44, 0.97) | 0.04 |
| IGDCC4:IGSF3 | 0.67 (0.21, 2.16) | 0.51 |
| IGDCC4:NCAN | 0.36 (0.12, 1.06) | 0.06 |
| IGDCC4:ADH1B | 0.81 (0.25, 2.65) | 0.73 |
| IGSF3:NCAN | 0.53 (0.29, 0.96) | 0.04 |
| IGSF3:ADH1B | 1.05 (0.54, 2.03) | 0.89 |
| NCAN:ADH1B | 1.41 (0.76, 2.59) | 0.28 |
| Abbreviations: HR, hazard ratio; CI, confidence interval; A1BG, Alpha-1B-glycoprotein; ACY1, Aminoacylase-1; DCXR, Dicarbonyl and L-xylulose reductase; ERBB3, Erb-B2 Receptor Tyrosine Kinase 3; F9, Coagulation Factor IX; FTCD, Formimidoyltransferase cyclodeaminase; IGDCC4, Immunoglobulin Superfamily DCC Subclass Member 4; IGSF3, Immunoglobulin superfamily member 3; NCAN, Neurocan core protein; ADH1B, Alcohol dehydrogenase 1B. Fully adjusted model was adjusted for gender, age, Townsend deprivation index, education, smoking status, alcohol consumption, body mass index, physical activity, healthy diet, waist circumference, hyperlipidemia, and hypertension. | | |

**Supplementary Table 18. Results of net reclassification improvement and integrated discrimination improvement.**

| **Disease** |  | **Train** | | **Validation** | |  | **Train** | | **Validation** | |
| --- | --- | --- | --- | --- | --- | --- | --- | --- | --- | --- |
|  |  | **NRI (95% CI)** | ***P*** | **NRI (95% CI)** | ***P*** |  | **IDI (95% CI)** | ***P*** | **IDI (95% CI)** | ***P*** |
| Chronic liver disease |  |  |  |  |  |  |  |  |  |  |
|  | LiverRisk score | Ref |  | Ref |  |  |  |  |  |  |
|  | LiverRisk score+ProRS | 0.5269 (0.3578, 0.6795) | <0.0001 | 0.3569 (0.1613, 0.5724) | <0.0001 |  | 0.0005 (0.0002, 0.0012) | <0.0001 | 0.0046 (0.0003, 0.0234) | <0.0001 |
| MASLD |  |  |  |  |  |  |  |  |  |  |
|  | LiverRisk score | Ref |  | Ref |  |  |  |  |  |  |
|  | LiverRisk score+ProRS | 0.5122 (0.2684, 0.7318) | <0.0001 | 0.2347 (-0.0142, 0.5156) | 0.076 |  | 0.0002 (0.0001, 0.0004) | <0.0001 | 0.0001 (-0.00004, 0.0003) | 0.244 |
| Alcoholic liver disease |  |  |  |  |  |  |  |  |  |  |
|  | LiverRisk score | Ref |  | Ref |  |  |  |  |  |  |
|  | LiverRisk score+ProRS | 0.2648 (-0.0526, 0.8389) | 0.192 | 0.5169 (0.0808, 0.8394) | <0.0001 |  | 0.0001 (0.0000, 0.0005) | 0.200 | 0.0074 (0.0003, 0.0905) | <0.0001 |
| Cirrhosis |  |  |  |  |  |  |  |  |  |  |
|  | LiverRisk score | Ref |  | Ref |  |  |  |  |  |  |
|  | LiverRisk score+ProRS | 0.4978 (0.2548, 0.7142) | <0.0001 | 0.9222 (0.8810, 0.9400) | <0.0001 |  | 0.0005 (0.0002, 0.0014) | <0.0001 | 0.0092 (0.0007, 0.0519) | <0.0001 |
| Composite hepatic event |  |  |  |  |  |  |  |  |  |  |
|  | LiverRisk score | Ref |  | Ref |  |  |  |  |  |  |
|  | LiverRisk score+ProRS | 0.1639 (-0.0264, 0.3693) | 0.128 | 0.3431 (0.0518, 0.6199) | 0.012 |  | 0.0001 (-0.00002, 0.0002) | 0.148 | 0.0003 (0.0000, 0.0012) | <0.0001 |
| Ascites |  |  |  |  |  |  |  |  |  |  |
|  | LiverRisk score | Ref |  | Ref |  |  |  |  |  |  |
|  | LiverRisk score+ProRS | 0.1123 (-0.0698, 0.5415) | 0.255 | 0.5050 (0.0226, 0.8349) | 0.044 |  | 0.0001 (0.0000, 0.0003) | 0.279 | 0.0002 (0.0000, 0.0004) | 0.052 |
| Spontaneous bacterial peritonitis |  |  |  |  |  |  |  |  |  |  |
|  | LiverRisk score | Ref |  | Ref |  |  |  |  |  |  |
|  | LiverRisk score+ProRS | 0.5381 (0.1827, 0.8414) | 0.004 | 0.5959 (0.0675, 0.8401) | 0.028 |  | 0.0003 (0.0001, 0.0004) | 0.004 | 0.0003 (0.0000, 0.0008) | 0.024 |
| Hepatic encephalopathy |  |  |  |  |  |  |  |  |  |  |
|  | LiverRisk score | Ref |  | Ref |  |  |  |  |  |  |
|  | LiverRisk score+ProRS | -0.0536 (-0.2428,0.2831) | 0.583 | 0.0850 (-0.2325, 0.4182) | 0.675 |  | 0.0000 (-0.0001, 0.0001) | 0.583 | 0.0001 (-0.0001, 0.0022) | 0.539 |
| Liver failure |  |  |  |  |  |  |  |  |  |  |
|  | LiverRisk score | Ref |  | Ref |  |  |  |  |  |  |
|  | LiverRisk score+ProRS | 0.9537 (0.9269, 0.9773) | 0.004 | 0.8639 (-0.1518, 0.9029) | 0.188 |  | 0.0005 (0.0005, 0.0005) | <0.0001 | 0.0004 (-0.0001, 0.0004) | 0.188 |

Abbreviations: NRI, net reclassification improvement; IDI, integrated discrimination improvement; ProSC, proteomic risk score; CI, confidence interval; MASLD, metabolic dysfunction-associated steatotic liver disease.

**Supplementary Table 19. Results of subgroup analyses.**

| **Group** | **Disease** | **Train** | | **Validation** | |
| --- | --- | --- | --- | --- | --- |
|  |  | **Cases/controls** | **AUC (95% CI)** | **Cases/controls** | **AUC (95% CI)** |
| Female | Chronic liver disease | 222/12994 | 0.76 (0.73, 0.80) | 86/5580 | 0.79 (0.74, 0.84) |
|  | MASLD | 163/13053 | 0.77 (0.73, 0.81) | 87/5579 | 0.79 (0.74, 0.84) |
|  | Alcoholic liver disease | 10/13206 | 0.81 (0.69, 0.92) | 7/5659 | 0.80 (0.54, 1.00) |
|  | Cirrhosis | 50/13166 | 0.80 (0.74, 0.87) | 24/5642 | 0.82 (0.72, 0.91) |
|  | Liver cancer | 2/13214 | - | 2/5664 | 0.57 (0.00, 1.00) |
|  | Composite hepatic event | 79/13137 | 0.64 (0.58, 0.70) | 36/5630 | 0.67 (0.58, 0.75) |
| Male | Chronic liver disease | 252/10874 | 0.76 (0.73, 0.79) | 116/4654 | 0.79 (0.75, 0.83) |
|  | MASLD | 160/10966 | 0.78 (0.74, 0.82) | 81/4689 | 0.76 (0.71, 0.82) |
|  | Alcoholic liver disease | 38/11088 | 0.82 (0.75, 0.90) | 10/4760 | 0.85 (0.72, 0.98) |
|  | Cirrhosis | 99/11027 | 0.81 (0.76, 0.87) | 38/4732 | 0.83 (0.76, 0.89) |
|  | Liver cancer | 9/11117 | 0.78 (0.60, 0.96) | 4/4766 | 0.94 (0.87, 1.00) |
|  | Composite hepatic event | 71/11055 | 0.63 (0.55, 0.70) | 38/4732 | 0.70 (0.62, 0.77) |
| < 50 years | Chronic liver disease | 70/5136 | 0.76 (0.71, 0.82) | 41/2190 | 0.81 (0.75, 0.87) |
|  | MASLD | 57/5149 | 0.78 (0.72, 0.83) | 28/2203 | 0.76 (0.67, 0.85) |
|  | Alcoholic liver disease | 17/5189 | 0.86 (0.78, 0.93) | 2/2229 | 0.59 (0.46, 0.71) |
|  | Cirrhosis | 19/5187 | 0.84 (0.74, 0.93) | 6/2225 | 0.83 (0.64, 1.00) |
|  | Liver cancer | 1/5205 | - | 0/2231 | - |
|  | Composite hepatic event | 13/5193 | 0.62 (0.42, 0.81) | 3/2228 | 0.76 (0.41, 1.00) |
| ≥ 50 years | Chronic liver disease | 397/18739 | 0.78 (0.75, 0.80) | 168/8037 | 0.76 (0.72, 0.80) |
|  | MASLD | 284/18852 | 0.77 (0.74, 0.80) | 122/8083 | 0.79 (0.75, 0.83) |
|  | Alcoholic liver disease | 28/19108 | 0.86 (0.78, 0.93) | 18/8187 | 0.86 (0.76, 0.95) |
|  | Cirrhosis | 124/19012 | 0.85 (0.81, 0.88) | 62/8143 | 0.76 (0.69, 0.83) |
|  | Liver cancer | 12/19124 | 0.82 (0.67, 0.97) | 4/8201 | 0.81 (0.55, 1.00) |
|  | Composite hepatic event | 147/18989 | 0.64 (0.59, 0.68) | 61/8144 | 0.62 (0.55, 0.70) |
| BMI ≤ 24 kg/m2 | Chronic liver disease | 47/5743 | 0.74 (0.66, 0.82) | 25/2457 | 0.78 (0.68, 0.87) |
|  | MASLD | 29/5761 | 0.79 (0.71, 0.87) | 8/2474 | 0.68 (0.46, 0.91) |
|  | Alcoholic liver disease | 10/5780 | 0.87 (0.75, 0.99) | 3/2479 | 0.96 (0.92, 1.00) |
|  | Cirrhosis | 22/5768 | 0.83 (0.75, 0.91) | 10/2472 | 0.72 (0.50, 0.95) |
|  | Liver cancer | 11/18541 | 0.77 (0.61, 0.94） | 6/7948 | 0.92 (0.80,1.00) |
|  | Composite hepatic event | 25/5765 | 0.69 (0.60, 0.78) | 12/2470 | 0.70 (0.59, 0.82) |
| BMI > 24 kg/m2 | Chronic liver disease | 425/18127 | 0.76 (0.74, 0.79) | 179/7775 | 0.76 (0.72, 0.80) |
|  | MASLD | 322/18230 | 0.76 (0.73, 0.79) | 132/7822 | 0.75 (0.71, 0.79) |
|  | Alcoholic liver disease | 39/18513 | 0.84 (0.76, 0.91) | 13/7941 | 0.81 (0.67, 0.95) |
|  | Cirrhosis | 129/18423 | 0.83 (0.80, 0.87) | 50/7904 | 0.81 (0.73, 0.89) |
|  | Liver cancer | 13/18539 | 0.93 (0.89, 0.97) | 4/7950 | 0.82 (0.48,1.00) |
|  | Composite hepatic event | 119/18433 | 0.63 (0.58, 0.68) | 68/7886 | 0.63 (0.56, 0.71) |

Abbreviations: MASLD, metabolic dysfunction-associated steatotic liver disease; AUC, area under the curve; CI, confidence interval; BMI, body mass index.

**Supplementary Table 20. 10-year absolute risk of liver-related outcomes across ProRS risk groups.**

| Outcome | ProRS group | N | Median 10-year absolute risk (%) | Proportion of participants above 5% threshold |
| --- | --- | --- | --- | --- |
| Chronic liver disease | Low | 11593 | 0.95 | 0 |
| Chronic liver disease | Moderate | 11593 | 0.96 | 0 |
| Chronic liver disease | High | 11592 | 1.01 | 0.16 |
| MASLD | Low | 11593 | 0.68 | 0 |
| MASLD | Moderate | 11593 | 0.69 | 0 |
| MASLD | High | 11592 | 0.72 | 0.07 |
| Alcoholic liver disease | Low | 11593 | 0.11 | 0 |
| Alcoholic liver disease | Moderate | 11593 | 0.11 | 0 |
| Alcoholic liver disease | High | 11592 | 0.12 | 0.02 |
| Cirrhosis | Low | 11593 | 0.32 | 0 |
| Cirrhosis | Moderate | 11593 | 0.33 | 0 |
| Cirrhosis | High | 11592 | 0.34 | 0.03 |
| Liver cancer | Low | 11593 | 0.03 | 0 |
| Liver cancer | Moderate | 11593 | 0.03 | 0 |
| Liver cancer | High | 11592 | 0.03 | 0.01 |
| Composite hepatic event | Low | 11593 | 0.42 | 0 |
| Composite hepatic event | Moderate | 11593 | 0.43 | 0 |
| Composite hepatic event | High | 11592 | 0.44 | 0.02 |
| Ascites | Low | 11593 | 0.13 | 0 |
| Ascites | Moderate | 11593 | 0.14 | 0 |
| Ascites | High | 11592 | 0.14 | 0.01 |
| Variceal Bleeding | Low | 11593 | 0 | 0 |
| Variceal Bleeding | Moderate | 11593 | 0 | 0 |
| Variceal Bleeding | High | 11592 | 0.01 | 0.01 |
| Spontaneous bacterial peritonitis | Low | 11593 | 0.13 | 0 |
| Spontaneous bacterial peritonitis | Moderate | 11593 | 0.14 | 0 |
| Spontaneous bacterial peritonitis | High | 11592 | 0.14 | 0.01 |
| Hepatic encephalopathy | Low | 11593 | 0.16 | 0 |
| Hepatic encephalopathy | Moderate | 11593 | 0.16 | 0 |
| Hepatic encephalopathy | High | 11592 | 0.17 | 0 |
| Liver failure | Low | 11593 | 0.06 | 0 |
| Liver failure | Moderate | 11593 | 0.06 | 0 |
| Liver failure | High | 11592 | 0.06 | 0.01 |

Abbreviations: MASLD, metabolic dysfunction-associated steatotic liver disease.
