# Supplementary material for: Proteomic Profiling of Plasma to Uncover Novel Intervention Targets and Prognostic Biomarkers for Chronic Liver Diseases
Source: Diabetes Obes Metab. 2026 Mar 26;28(6):5029–42. doi: 10.1111/dom.70696 (PMC13146168; doi:10.1111/dom.70696)
Supplement: Supplementary file 2 — Figure S1: Flowchart for the selection of study participants. Figure S2: Regional association plot for colocalization analysis of proteins identified by cis‐MR with CLDs risk. Figure S3: Regional association plot for colocalization analysis of proteins identified by all‐MR with MASLD risk. Figure S4: Regional association plot for colocalization analysis of proteins identified by all‐MR with ALD risk. Figure S5: Regional association plot for colocalization analysis of proteins identified by all‐MR with cirrhosis risk. Figure S6: The correlation heat map of covariates. Figure S7: Results of single‐cell expression analysis. Figure S8: Results of pathway enrichment analysis. Figure S9: Decision curve analysis in the (A) training and (B) validation datasets. Figure S10: Calibration plots of proteomic risk score for chronic liver disease and composite hepatic event. Figure S11: The cumulative incidence curves for chronic liver disease stratified by proteomic risk score tertiles. Figure S12: The cumulative incidence curves for composite hepatic event stratified by proteomic risk score tertiles. [file DOM-28-5029-s002.docx]

**Supplemental Figures**

**[Figure S1. Flowchart for the selection of study participants.](#F1)**

**[Figure S2. Regional association plot for colocalization analysis of proteins identified by](#F2) *[cis](#F2)*[-MR with CLDs risk.](#F2)**

**[Figure S3. Regional association plot for colocalization analysis of proteins identified by all-MR with MASLD risk.](#F3)**

**[Figure S4. Regional association plot for colocalization analysis of proteins identified by all-MR with ALD risk.](#F4)**

**[Figure S5. Regional association plot for colocalization analysis of proteins identified by all-MR with cirrhosis risk.](#F5)**

**[Figure S6. The correlation heat map of covariates.](#F6)**

**[Figure S7. Results of](#F7) [single-cell expression analysis.](#F6)**

**[Figure S8. Results of pathway enrichment analysis.](#F7)**

**[Figure S9](#F6)**[.](#F6) **[Decision curve analysis in the (A) training and (B) validation datasets.](#F6)**

**[Figure S10. Calibration plots of proteomic risk score for chronic liver disease and composite hepatic event.](#F7)**

**[Figure S11. The cumulative incidence curves for chronic liver disease stratified by proteomic risk score tertiles.](#F8)**

**[Figure S12. The cumulative incidence curves for composite hepatic event stratified by proteomic risk score tertiles.](#F9)**

**
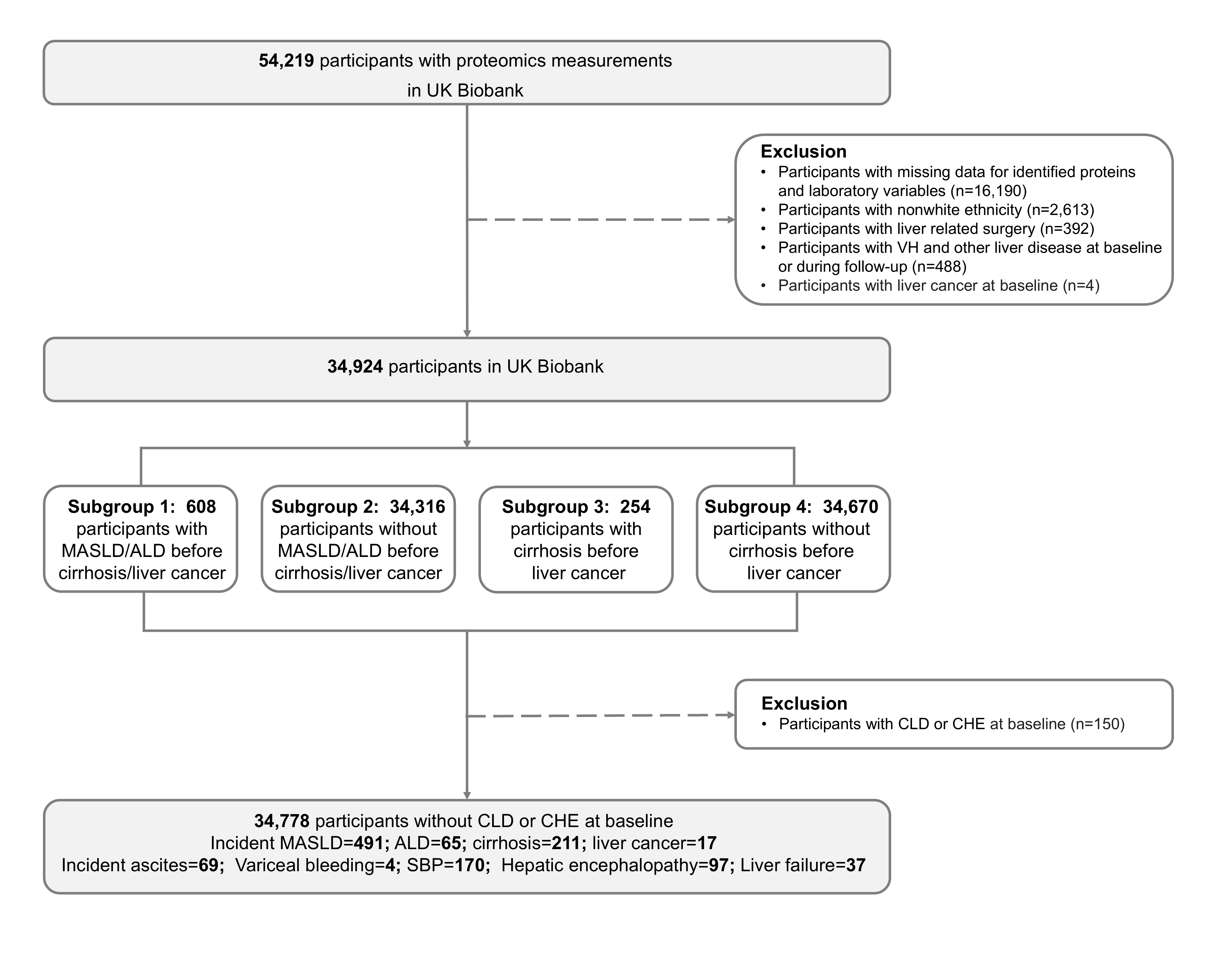
**

**Figure S1.** Flowchart for the selection of study participants.

Abbreviations: MASLD, metabolic dysfunction-associated steatotic liver disease; ALD, alcoholic liver disease; VH, viral hepatitis; CLD, chronic liver disease; CHE, composite hepatic event; SBP, spontaneous bacterial peritonitis.

**
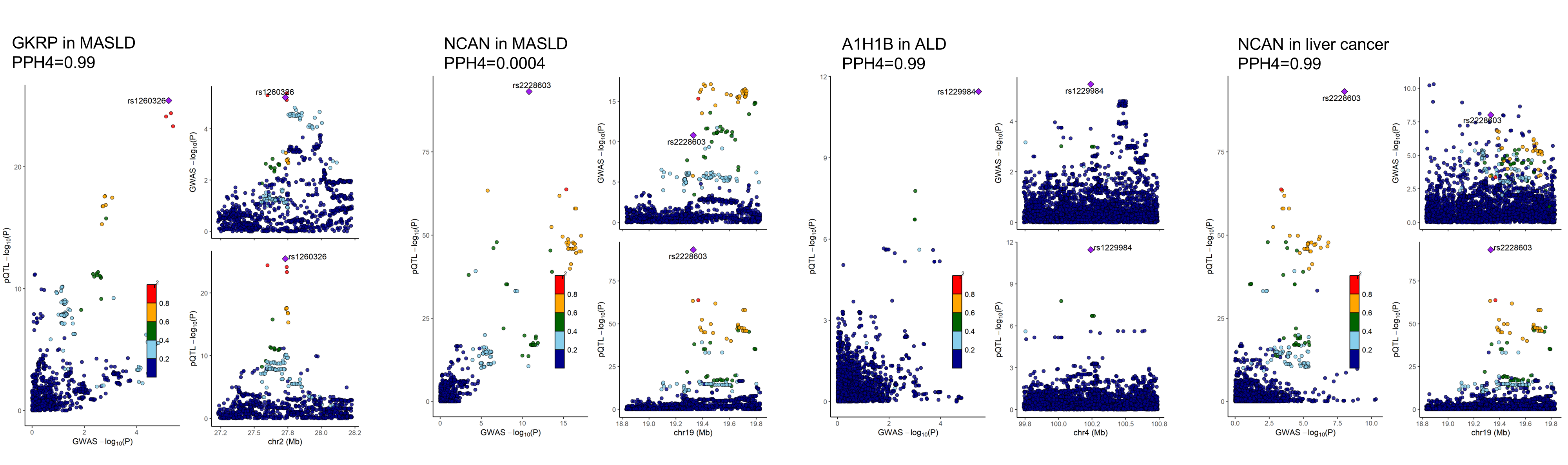
**

**Figure S2**. Regional association plot for colocalization analysis of proteins identified by *cis*-MR with CLDs risk. The lead SNP is shown as a purple diamond. Abbreviations: MASLD, metabolic dysfunction-associated steatotic liver disease; ALD, alcoholic liver disease.


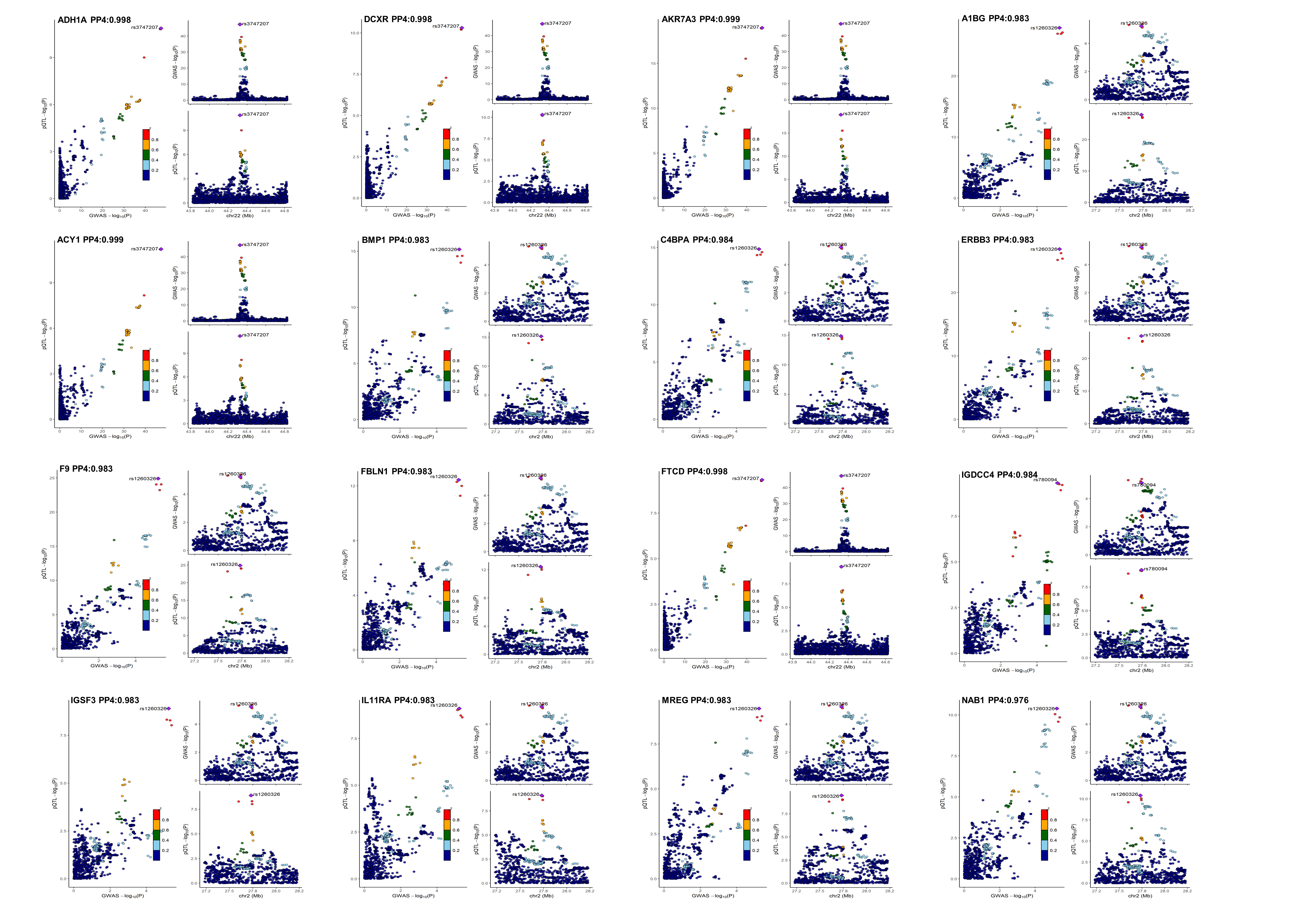


**Figure S3**. Regional association plot for colocalization analysis of proteins identified by all-MR with MASLD risk. The lead SNP is shown as a purple diamond.


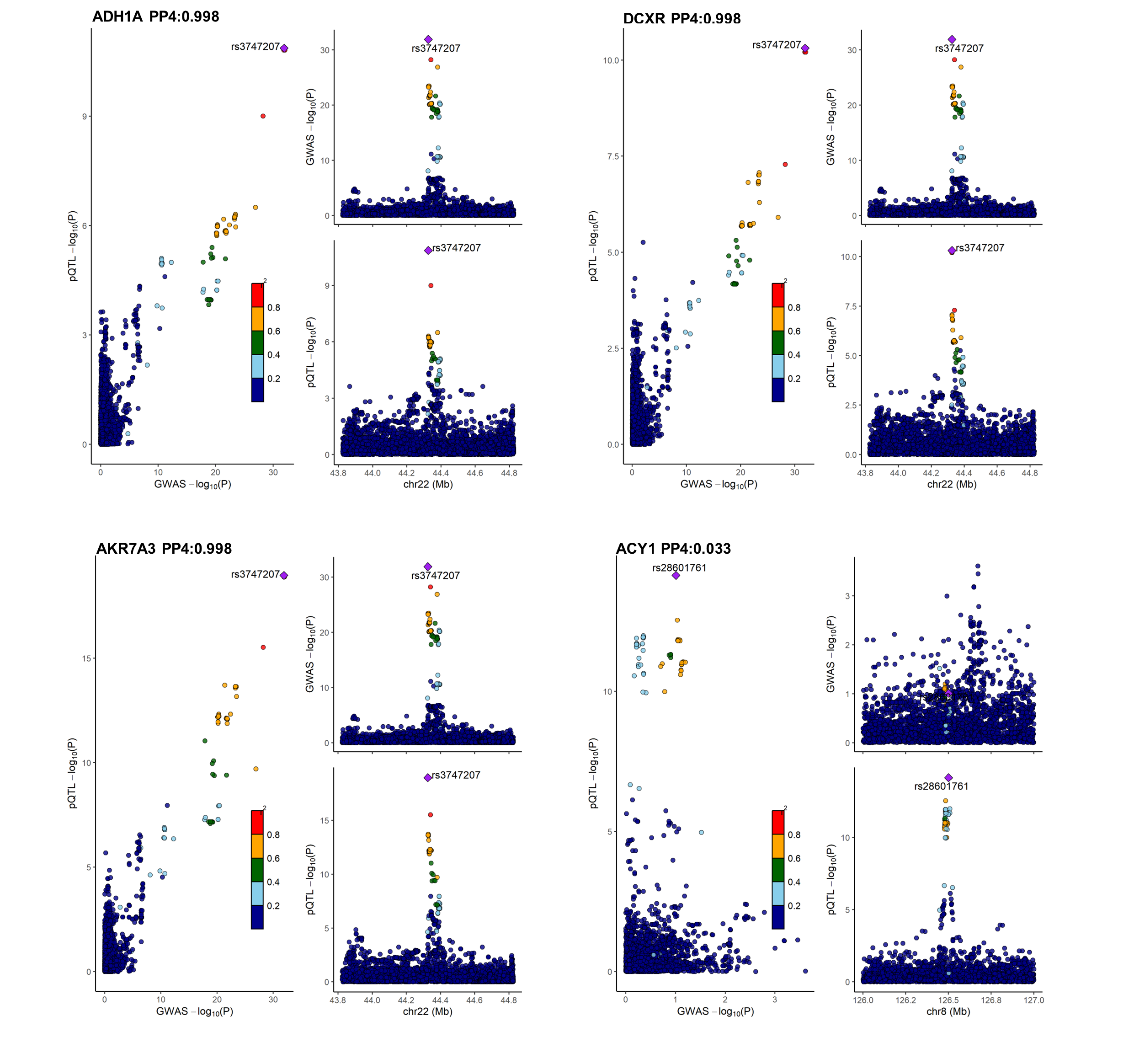


**Figure S4**. Regional association plot for colocalization analysis of proteins identified by all-MR with ALD risk. The lead SNP is shown as a purple diamond.


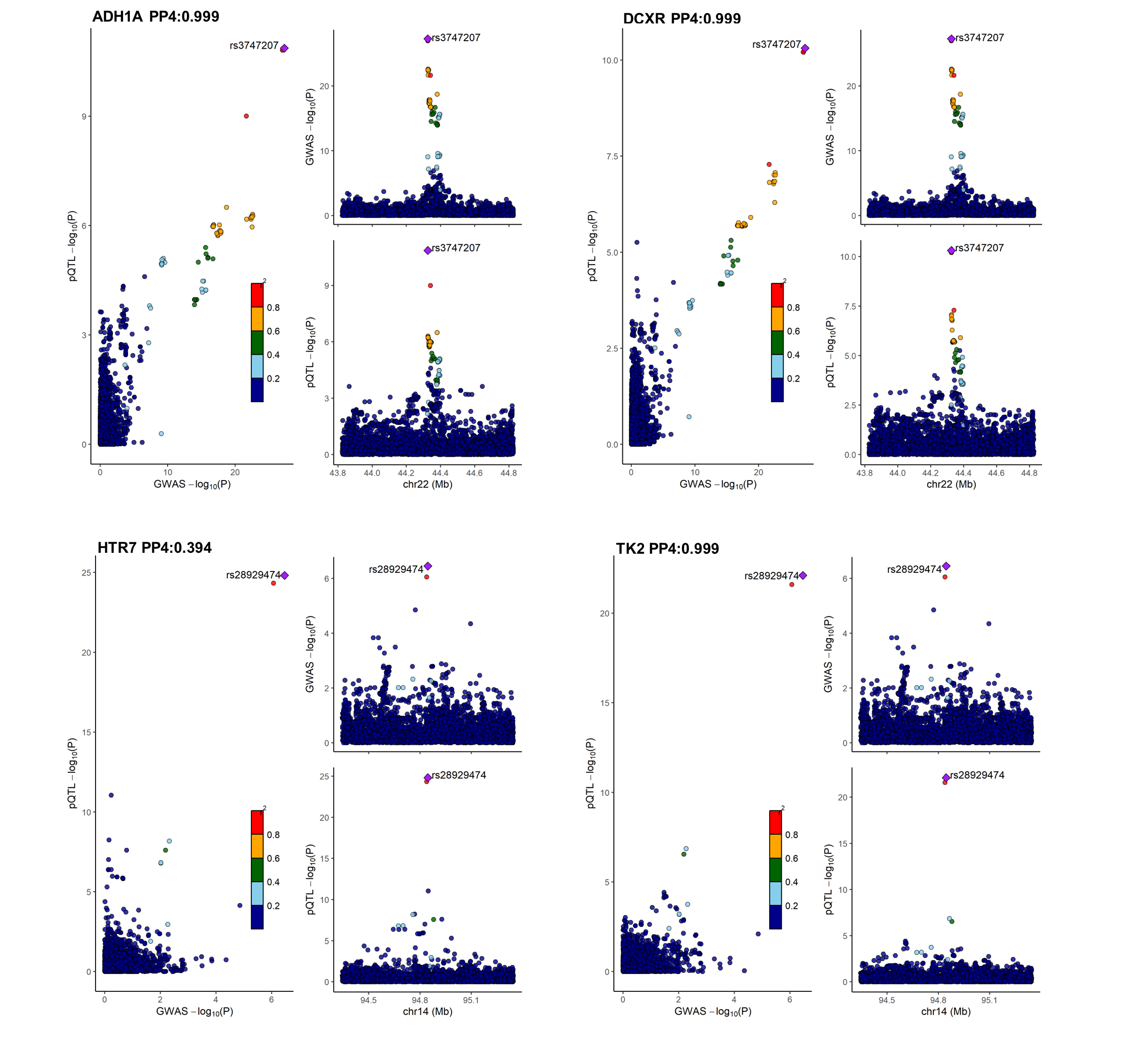


**Figure S5**. Regional association plot for colocalization analysis of proteins identified by all-MR with cirrhosis risk. The lead SNP is shown as a purple diamond.


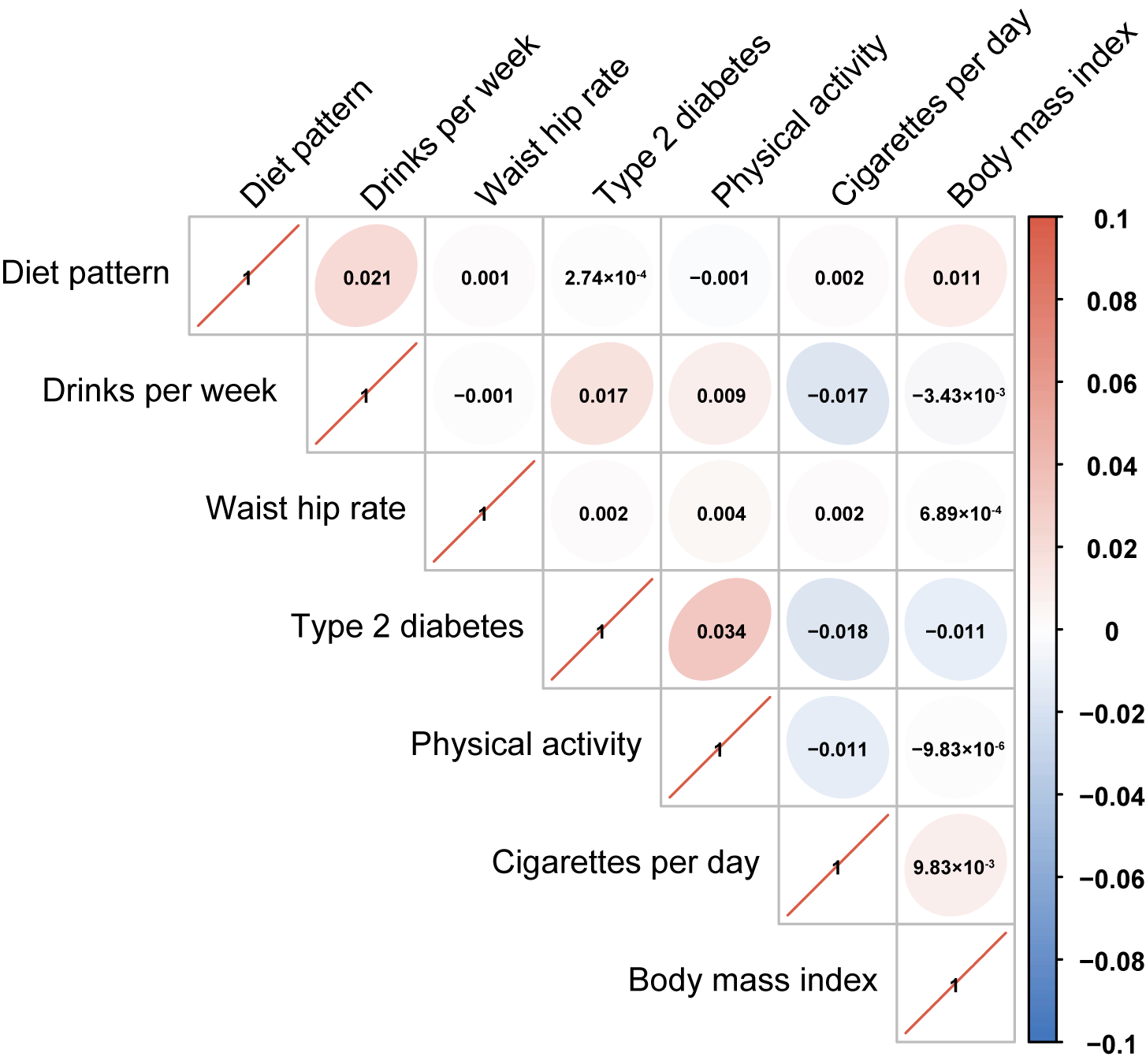


**Figure S6**. The correlation heat map of covariates.

**[Figure S7.](#F7)** [Results](#F7)[of](#F7)single-cell expression analysis.

**Figure S8**. Results of pathway enrichment analysis.


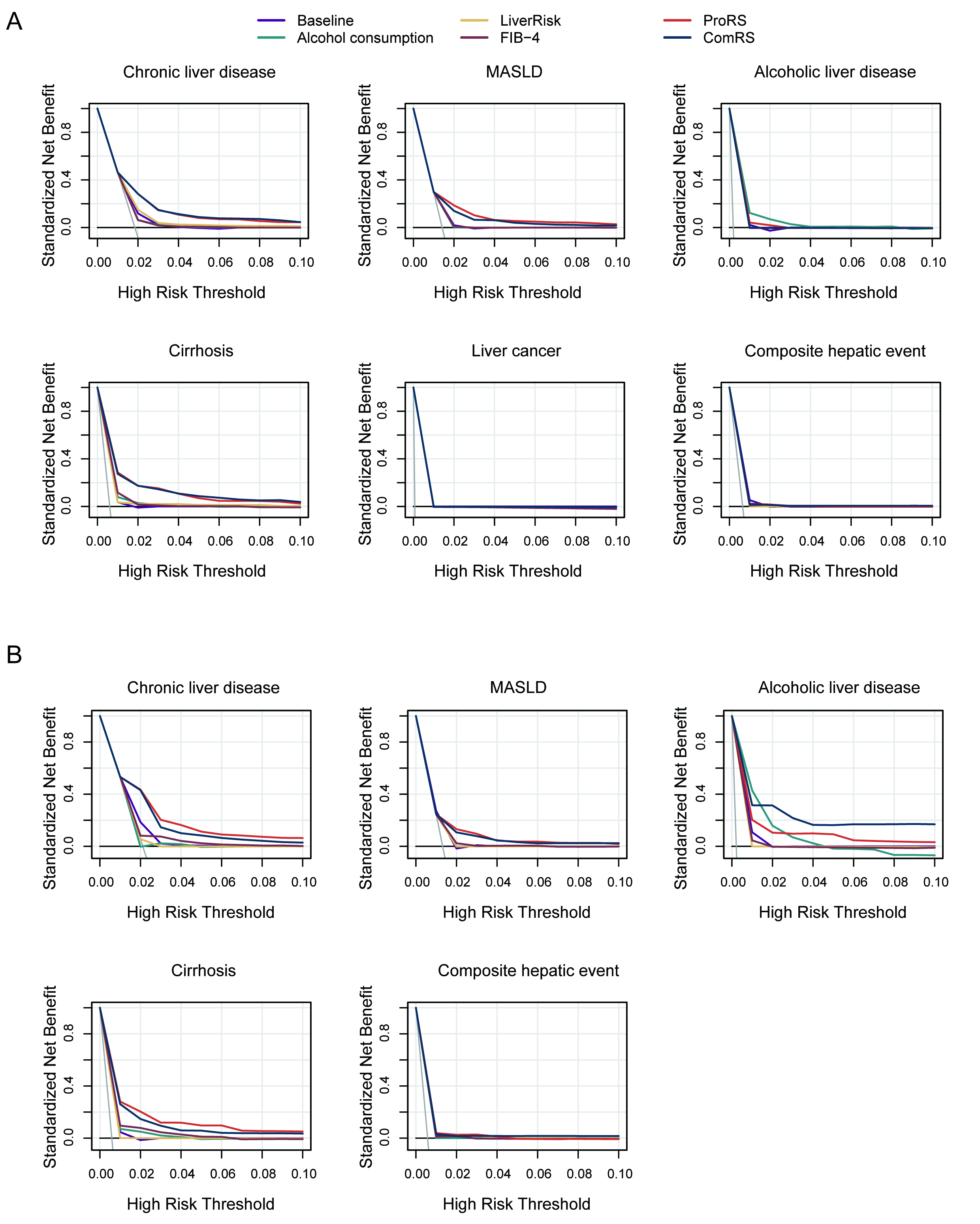


**Figure S9**. Decision curve analysis in the (A) training and (B) validation datasets. Abbreviations: MASLD, metabolic dysfunction-associated steatotic liver disease; ProSC, proteomic risk score; ComRS, combined risk score; FIB-4 index, fibrosis-4 index.


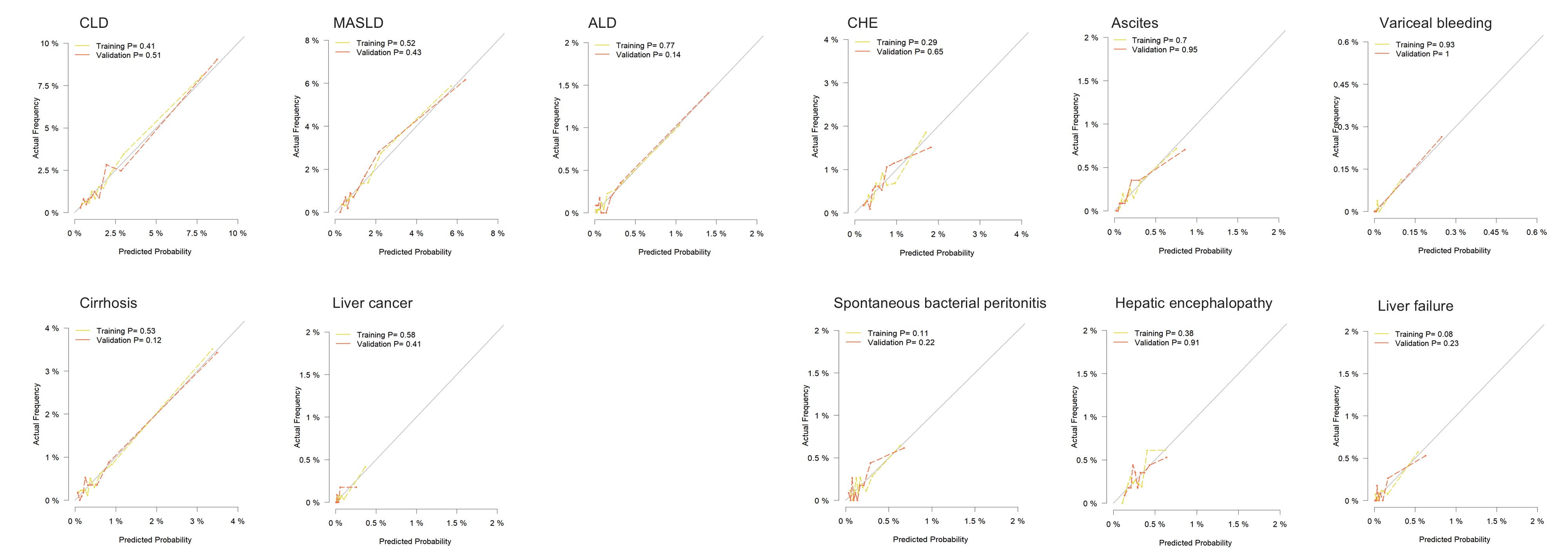


**Figure S10**. Calibration plots of proteomic risk score for chronic liver disease and composite hepatic event. Abbreviations: CLD, chronic liver disease; MASLD, metabolic dysfunction-associated steatotic liver disease; ALD, alcoholic liver disease; CHE, composite hepatic event.


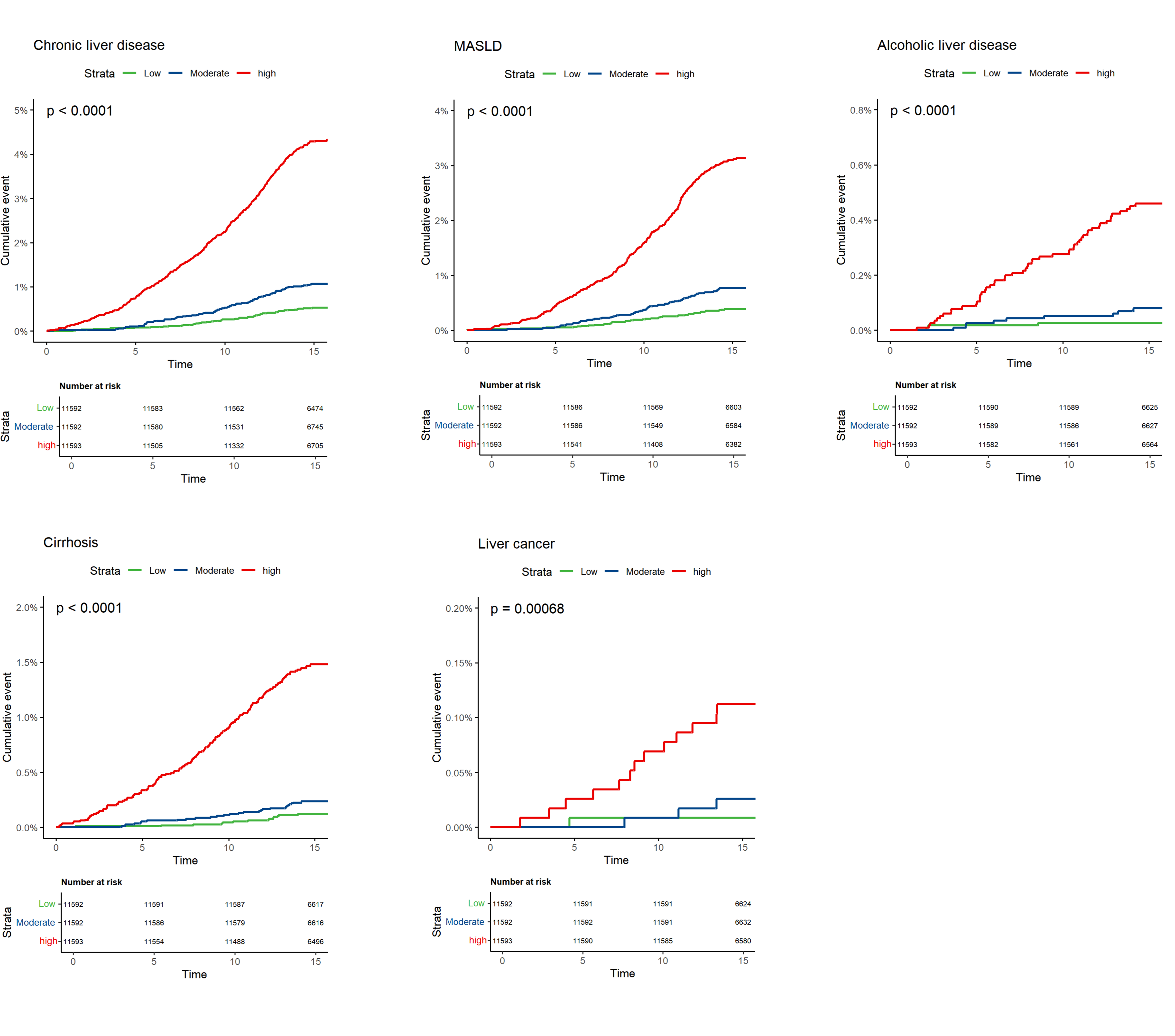


**Figure S11**. The cumulative incidence curves for chronic liver disease stratified by proteomic risk score tertiles. Abbreviations: MASLD, metabolic dysfunction-associated steatotic liver disease.


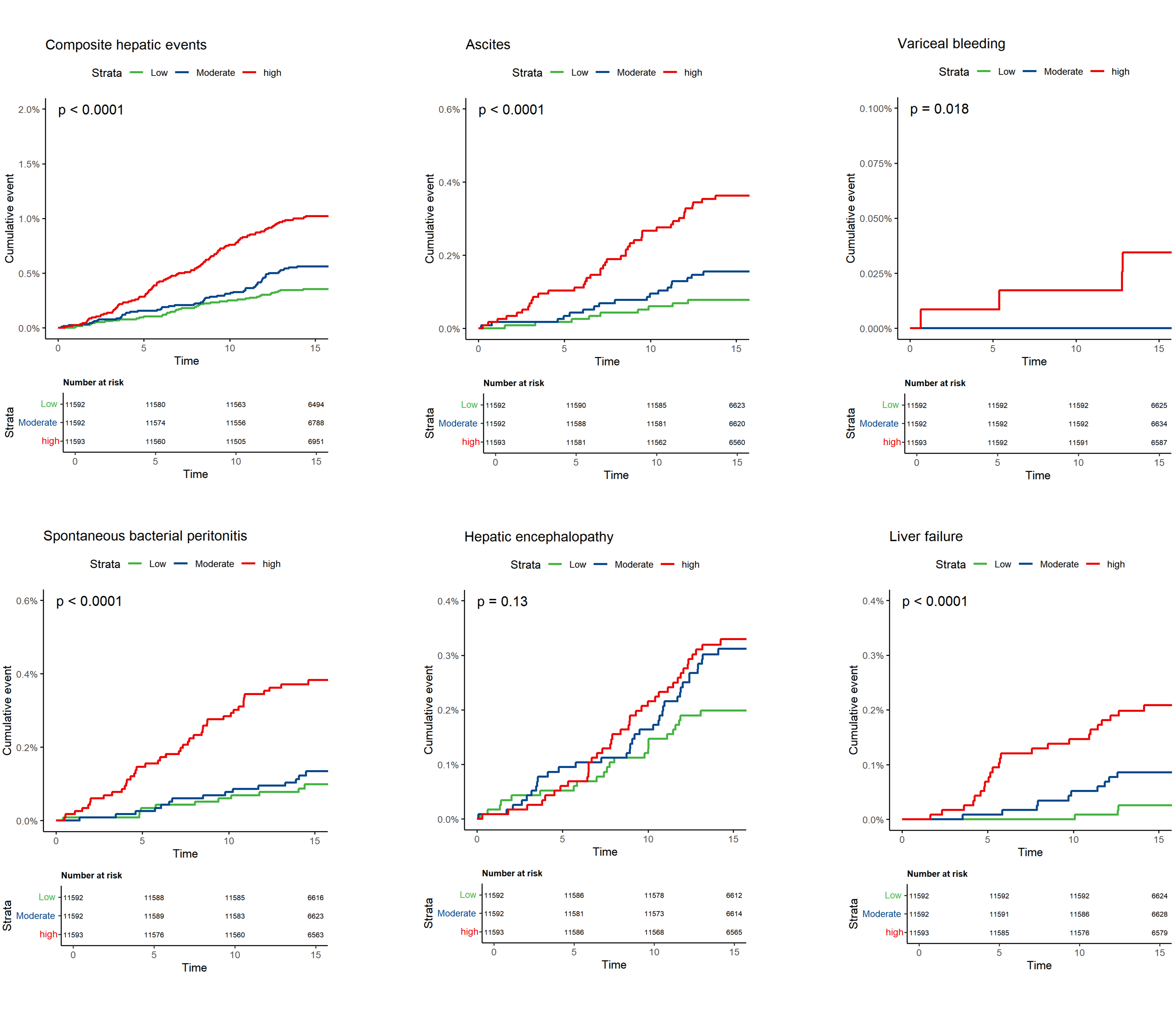
**Figure S12**. The cumulative incidence curves for composite hepatic event stratified by proteomic risk score tertiles.
